# Supplementary material for: Pre-COVID-19 pandemic health-related behaviours in children (2018–2020) and association with being tested for SARS-CoV-2 and testing positive for SARS-CoV-2 (2020–2021): a retrospective cohort study using survey data linked with routine health data in Wales, UK
Source: BMJ Open. 2022 Sep 7;12(9):e061344. doi: 10.1136/bmjopen-2022-061344 (PMC9453425; doi:10.1136/bmjopen-2022-061344)
Supplement: Supplementary data [file bmjopen-2022-061344supp002.pdf]

Online supplemental appendix 2: RECORD statement

The RECORD statement – checklist of items, extended from the STROBE statement, that should be reported in observational studies using routinely collected health data.

	Item No.	STROBE items	Location in manuscript where items are reported	RECORD items	Location in manuscript where items are reported
**Title and abstract**					
	1	(a) Indicate the study's design with a commonly used term in the title or the abstract (b) Provide in the abstract an informative and balanced summary of what was done and what was found		RECORD 1.1: The type of data used should be specified in the title or abstract. When possible, the name of the databases used should be included.<br><br>RECORD 1.2: If applicable, the geographic region and timeframe within which the study took place should be reported in the title or abstract.<br><br>RECORD 1.3: If linkage between databases was conducted for the study, this should be clearly stated in the title or abstract.	1.1: Title and abstract<br><br>1.2: Title and abstract
**Introduction**					
Background rationale	2	Explain the scientific background and rationale for the investigation being reported			Background
Objectives	3	State specific objectives, including any prespecified hypotheses			Background
**Methods**					
Study Design	4	Present key elements of study design early in the paper			Methods - Study design
Setting	5	Describe the setting, locations, and relevant dates, including			Methods - Study design

Online supplemental appendix 2: RECORD statement

		periods of recruitment, exposure, follow-up, and data collection			
Participants	6	*(a) Cohort study* - Give the eligibility criteria, and the sources and methods of selection of participants. Describe methods of follow-up<br>*Case-control study* - Give the eligibility criteria, and the sources and methods of case ascertainment and control selection. Give the rationale for the choice of cases and controls<br>*Cross-sectional study* - Give the eligibility criteria, and the sources and methods of selection of participants<br><br>*(b) Cohort study* - For matched studies, give matching criteria and number of exposed and unexposed<br>*Case-control study* - For matched studies, give matching criteria and the number of controls per case		RECORD 6.1: The methods of study population selection (such as codes or algorithms used to identify subjects) should be listed in detail. If this is not possible, an explanation should be provided.<br><br>RECORD 6.2: Any validation studies of the codes or algorithms used to select the population should be referenced. If validation was conducted for this study and not published elsewhere, detailed methods and results should be provided.<br><br>RECORD 6.3: If the study involved linkage of databases, consider use of a flow diagram or other graphical display to demonstrate the data linkage process, including the number of individuals with linked data at each stage.	6.1: Figure 1: Cohort Flow Diagram<br><br>6.3: Figure 1: Cohort Flow Diagram
Variables	7	Clearly define all outcomes, exposures, predictors, potential confounders, and effect modifiers. Give diagnostic criteria, if applicable.		RECORD 7.1: A complete list of codes and algorithms used to classify exposures, outcomes, confounders, and effect modifiers should be provided. If these cannot be reported, an explanation should be provided.	7.1: Supplemental appendix 4: HAPPEN survey variable codebook
Data sources/ measurement	8	For each variable of interest, give sources of data and details of methods of assessment (measurement).			Methods - The HAPPEN survey and linked SAIL data

Online supplemental appendix 2: RECORD statement

		Describe comparability of assessment methods if there is more than one group			
Bias	9	Describe any efforts to address potential sources of bias			Methods - Quantitative analysis
Study size	10	Explain how the study size was arrived at			Figure 1: Cohort flow diagram
Quantitative variables	11	Explain how quantitative variables were handled in the analyses. If applicable, describe which groupings were chosen, and why			Methods - Quantitative analysis
Statistical methods	12	(a) Describe all statistical methods, including those used to control for confounding<br>(b) Describe any methods used to examine subgroups and interactions<br>(c) Explain how missing data were addressed<br>(d) *Cohort study* - If applicable, explain how loss to follow-up was addressed<br>*Case-control study* - If applicable, explain how matching of cases and controls was addressed<br>*Cross-sectional study* - If applicable, describe analytical methods taking account of sampling strategy<br>(e) Describe any sensitivity analyses			Methods - Quantitative analysis
Data access and cleaning methods		..		RECORD 12.1: Authors should describe the extent to which the	12.1: Methods - The HAPPEN

Online supplemental appendix 2: RECORD statement

				investigators had access to the database population used to create the study population.<br><br>RECORD 12.2: Authors should provide information on the data cleaning methods used in the study.	survey and linked SAIL data<br><br>12.2: Figure 1 – Cohort flow diagram
Linkage		..		RECORD 12.3: State whether the study included person-level, institutional-level, or other data linkage across two or more databases. The methods of linkage and methods of linkage quality evaluation should be provided.	12.3: Methods - Study design<br><br>The HAPPEN survey and linked SAIL data
**Results**					
Participants	13	(a) Report the numbers of individuals at each stage of the study (*e.g.*, numbers potentially eligible, examined for eligibility, confirmed eligible, included in the study, completing follow-up, and analysed)<br>(b) Give reasons for non-participation at each stage.<br>(c) Consider use of a flow diagram		RECORD 13.1: Describe in detail the selection of the persons included in the study (*i.e.,* study population selection) including filtering based on data quality, data availability and linkage. The selection of included persons can be described in the text and/or by means of the study flow diagram.	13.1: Methods - Quantitative analysis<br><br>Figure 1: Cohort flow diagram
Descriptive data	14	(a) Give characteristics of study participants (*e.g.*, demographic, clinical, social) and information on exposures and potential confounders<br>(b) Indicate the number of participants with missing data for each variable of interest<br>(c) *Cohort study* - summarise follow-up time (*e.g.*, average and total amount)			Results - Table 1 Descriptive statistics<br>Full descriptive statistics table: Online supplemental appendix 5

Online supplemental appendix 2: RECORD statement

Outcome data	15	*Cohort study* - Report numbers of outcome events or summary measures over time<br>*Case-control study* - Report numbers in each exposure category, or summary measures of exposure<br>*Cross-sectional study* - Report numbers of outcome events or summary measures			Results - Table 1 Descriptive statistics<br>Full descriptive statistics table: Online supplemental appendix 6
Main results	16	(a) Give unadjusted estimates and, if applicable, confounder-adjusted estimates and their precision (e.g., 95% confidence interval). Make clear which confounders were adjusted for and why they were included<br>(b) Report category boundaries when continuous variables were categorized<br>(c) If relevant, consider translating estimates of relative risk into absolute risk for a meaningful time period			Results – Table 3 and table 4<br><br>Online supplemental appendix 6: Unadjusted multivariable logistic regression analyses
Other analyses	17	Report other analyses done—e.g., analyses of subgroups and interactions, and sensitivity analyses			Results
**Discussion**					
Key results	18	Summarise key results with reference to study objectives			Results
Limitations	19	Discuss limitations of the study, taking into account sources of potential bias or imprecision. Discuss both direction and magnitude of any potential bias		RECORD 19.1: Discuss the implications of using data that were not created or collected to answer the specific research question(s). Include discussion of misclassification bias,	Strengths and limitations

Online supplemental appendix 2: RECORD statement

				unmeasured confounding, missing data, and changing eligibility over time, as they pertain to the study being reported.	
Interpretation	20	Give a cautious overall interpretation of results considering objectives, limitations, multiplicity of analyses, results from similar studies, and other relevant evidence			Discussion<br>Conclusion
Generalisability	21	Discuss the generalisability (external validity) of the study results			Discussion<br>Conclusion<br>Strengths and limitations
**Other Information**					
Funding	22	Give the source of funding and the role of the funders for the present study and, if applicable, for the original study on which the present article is based			Funding
Accessibility of protocol, raw data, and programming code		..		RECORD 22.1: Authors should provide information on how to access any supplemental information such as the study protocol, raw data, or programming code.	Availability for data and materials

*Reference: Benchimol EI, Smeeth L, Guttmann A, Harron K, Moher D, Petersen I, Sørensen HT, von Elm E, Langan SM, the RECORD Working Committee. The REporting of studies Conducted using Observational Routinely-collected health Data (RECORD) Statement. *PLoS Medicine* 2015; in press.

*Checklist is protected under Creative Commons Attribution (CC BY) license.
