# Supplementary material for: Pre-COVID-19 pandemic health-related behaviours in children (2018–2020) and association with being tested for SARS-CoV-2 and testing positive for SARS-CoV-2 (2020–2021): a retrospective cohort study using survey data linked with routine health data in Wales, UK
Source: BMJ Open. 2022 Sep 7;12(9):e061344. doi: 10.1136/bmjopen-2022-061344 (PMC9453425; doi:10.1136/bmjopen-2022-061344)
Supplement: Supplementary data [file bmjopen-2022-061344supp004.pdf]

Online supplemental appendix 4: HAPPEN survey variable codebook

Exposures	HAPPEN Survey item	Responses	Analyses coding
**Ate breakfast**	*13. What did you eat for breakfast yesterday?*	*Nothing*<br>*Cereal*<br>*Snacks*<br>*Fruit*<br>*Toast*<br>*Cooked breakfast*<br>*Yoghurt*	Binary:<br>*1 = Cereal; Snacks; Fruit; Toast; Cooked breakfast; Yoghurt*<br>*0 = Nothing*
**Active travel to school**	*14. How did you get to school yesterday morning?*	*On the bus*<br>*In the car/taxi*<br>*Walked*<br>*On bike*<br>*Ran/jogged*<br>*Scooter*<br>*Skateboarded/rollerbladed*	Binary:<br>*1 = Walked; On bike; Ran/jogged; Scooter; Skateboarded/rollerbladed*<br>*0 = On the bus; In the car/taxi*
**Active travel from school**	*18. How did you get home yesterday?*	*On the bus*<br>*In the car/taxi*<br>*Walked*<br>*On bike*<br>*Ran/jogged*<br>*Scooter*<br>*Skateboarded/rollerbladed*	Binary:<br>*1 = Walked; On bike; Ran/jogged; Scooter; Skateboarded/rollerbladed*<br>*0 = On the bus; In the car/taxi*
**Toothbrush 2+ per day**	*20. How many times did you brush your teeth yesterday?*	*0 – 3*	Continuous:<br>*0 – 3*
**5+ fruit and veg**	*19. How many portions of fruit and vegetables did you eat yesterday?*	*0 – 8*	Continuous:<br>*0 – 8*

Online supplemental appendix 4: HAPPEN survey variable codebook

**Sleep 9+ hours**	*21. What time did you fall asleep last night*	*(30 min intervals)*<br>*7:00pm – 4:00am*	Continuous:<br>*Sleep hours calculated from item 21 and 22*
	22. What time did you wake up this morning?	*(30 min intervals)*<br>*5:00am – 9:00am*	
**Physically active 60+ mins every day previous 7 days**	*23. In the last 7 days, how many days did you do sports or exercise for at least 1 hour in total (This includes doing any activities or playing sports where your heart beat faster, you breathed faster and you felt warmer*	*0 days*<br>*1 – 2 days*<br>*3 – 4 days*<br>*5 – 6 days*<br>*7 days*	Ordinal:<br>*0 days*<br>*1 – 2 days*<br>*3 – 4 days*<br>*5 – 6 days*<br>*7 days*
**Sedentary/screen time 2 hours every day previous 7 days**	*24. In the last 7 days, how many days did you watch TV/play online games/use the internet etc. for 2 or more hours a day (in total)?*	*0 days*<br>*1 – 2 days*<br>*3 – 4 days*<br>*5 – 6 days*<br>*7 days*	Ordinal:<br>*0 days*<br>*1 – 2 days*<br>*3 – 4 days*<br>*5 – 6 days*<br>*7 days*
**Tired 7 days**	*25. In the last 7 days, how many days did you feel tired?*	*0 days*<br>*1 – 2 days*<br>*3 – 4 days*<br>*5 – 6 days*<br>*7 days*	Ordinal:<br>*0 days*<br>*1 – 2 days*<br>*3 – 4 days*<br>*5 – 6 days*<br>*7 days*

Online supplemental appendix 4: HAPPEN survey variable codebook

**Sugary snack 7 days**	*28. In the last 7 days, how many days did you eat at least one sugary snack (e.g. chocolate bar, sweets)*	*0 days*<br>*1 – 2 days*<br>*3 – 4 days*<br>*5 – 6 days*<br>*7 days*	Ordinal:<br>*0 days*<br>*1 – 2 days*<br>*3 – 4 days*<br>*5 – 6 days*<br>*7 days*
Participate in at least 3 out of school clubs	*31. How many times do you take part in a sports club OUTSIDE OF SCHOOL each week?*	*0 - 10*	Continuous:<br>*0 - 10*
Can ride a bike	*35. Can you ride a bike without stabilisers?*	*No*<br>*Yes*	Binary:<br>*1 = Yes*<br>*0 = No*
Can swim 25m	*36. Can you swim 25 metres without a float or armbands (This is 1 length of a standard swimming pool)*	*No*<br>*Yes*	Binary:<br>*1 = Yes*<br>*0 = No*
Age on 01/03/2020	*Decimal age on 1 March 2020*	Continuous	Continuous
Sex	*Sex*	*Girl*<br>*Boy*	Binary:<br>*0 = Girl*<br>*1 = Boy*
WIMD	*Welsh Index of Multiple Deprivation 2019*		Coding framework from WIMD 2019[34]
