# Supplementary material for: Pre-COVID-19 pandemic health-related behaviours in children (2018–2020) and association with being tested for SARS-CoV-2 and testing positive for SARS-CoV-2 (2020–2021): a retrospective cohort study using survey data linked with routine health data in Wales, UK
Source: BMJ Open. 2022 Sep 7;12(9):e061344. doi: 10.1136/bmjopen-2022-061344 (PMC9453425; doi:10.1136/bmjopen-2022-061344)
Supplement: Supplementary data [file bmjopen-2022-061344supp005.pdf]

Online supplemental appendix 5: Full descriptive statistics table by tested for SARS-CoV-2 and tested positive for SARS-CoV-2.

		Tested for SARS-CoV-2 % (n)	Not tested for SARS-CoV-2 % (n)	Tested positive for SARS-CoV-2 % (n)	Tested negative/not tested (unknown) for SARS-CoV-2 % (n)
		**LINKED DATA**			
**Sample**		39.1% (2,764)	60.9% (4,298)	8.1% (569)	91.9% (6,493)
**Age at time of HAPPEN survey**		10.1 ± 0.8	9.9 ± 0.9	10.1 ± 0.8	9.9 ± 0.8
**Age on 01/03/2020 (start of period of interest)**		10.6 ± 0.9	10.3 ± 1.1	10.6 ± 1.0	10.4 ± 1.0
**Sex**	**Boy**	49.3% (1,363)	46.7% (2,005)	44.3% (252)	48.0% (3,116)
	**Girl**	48.9% (1,352)	51.8% (2,226)	54.5% (310)	50.3% (3,268)
	Missing	*1.8% (49)*	*1.5% (67)*	1.2% (7)	1.7% (109)
**WIMD 2019 quintiles**	**1 (most deprived)**	24.3% (672)	23.9% (1,025)	28.5% (162)	23.6% (1,535)
	**2**	19.9% (551)	19.02% (826)	19.7% (112)	19.5% (1,265)
	**3**	16.5% (455)	17.4% (748)	17.6% (100)	17.0% (1,103)
	**4**	15.6% (431)	15.8% (678)	14.1% (80)	15.9% (1,029)
	**5 (least deprived)**	18.0% (497)	16.8% (771)	16.5% (94)	17.3% (1,124)
	Missing	*5.7% (158)*	*7.0% (300)*	*3.7% (21)*	*6.7% (437)*
		**HAPPEN SURVEY**			

		**Previous day**			
**Ate breakfast**	**Yes**	93.0% (2,571)	92.1% (3,797)	93.4% (538)	92% (6,012)
	**No**	7% (193)	7.3% (319)	5.6% (31)	7.3% (481)
	Missing	*0%*	*0%*	*0%*	*0%*
**Active travel to school**	**Yes**	38.5% (1,065)	39.8% (1,710)	37.6% (214)	39.4% (2,561)
	**No**	61.5% (1,699)	60.2% (2,588)	62.4% (355)	60.6% (3,932)
	Missing	*0%*	*0%*	*0%*	*0%*
**Active travel from school**	**Yes**	43.0% (1,187)	43.0% (1,846)	42.4% (241)	43.0% (2,792)
	**No**	57.0% (1,577)	57.0% (2,452)	57.6% (328)	57.0% (3,701)
	Missing	*0%*	*0%*	*0%*	*0%*
**Toothbrush continuous**	**0**	3.3% (91)	3.4% (146)	1.9% (11)	3.5% (227)
	**1**	20.0% (552)	21.0% (903)	18.6% (106)	20.6% (1,358)
	**2**	67.1% (1,854)	65.2% (2,802)	69.6% (396)	65.2% (4,294)
	**3**	9.6% (265)	10.3% (446)	9.5% (54)	10.0% (659)
	Missing	*0.1% (<5)*	*<0.1% (<5)*	*0.4% (<5)*	*<0.1%% (<5)*
**Fruit/veg portions (continuous)**	**0**	14.3% (395)	15.3% (657)	12.5% (71)	15.1% (981)
	**1**	16.1% (445)	17.4% (749)	15.8% (90)	17.0% (1,104)
	**2**	17.7% (489)	17.5% (754)	19.5% (111)	17.4% (1,132)
	**3**	17.5% (484)	16.5% (711)	16.7% (95)	16.9% (1,110)
	**4**	12.7% (351)	11.9% (510)	13.5% (77)	12.1% (784)
	**5**	10.5% (291)	10.6% (455)	11.8% (67)	10.4% (679)
	**6**	4.5% (123)	4.3% (186)	2.8% (16)	4.5% (293)
	**7**	2.3% (63)	2.1% (92)	4.2% (24)	2.0% (131)
	**8**	4.5% (123)	4.3% (184)	3.2% (18)	4.5% (289)
	Missing	0%	0%	*0%*	*0%*

**Sleep hours**		9.4 ± 1.6	9.4 ± 1.6	9.4 ± 1.6	9.4 ± 1.6
**Number of days physically active ≥ 60 minutes**	**0**	6.5% (179)	7.9% (339)	4.0% (23)	7.6% (495)
	**1-2 days**	27.9% (772)	29.0% (1,246)	27.8% (158)	28.7% (1,860)
	**3-4 days**	27.5% (761)	26.2% (1,128)	30.9% (176)	26.4% (1,712)
	**5-6 days**	18.3% (505)	17.0% (731)	18.1% (103)	17.5% (1,133)
	**7 days**	19.8% (557)	19.9% (854)	19.2% (109)	19.9% (1,292)
	Missing	*0%*	*0%*	*0%*	*0%*
**Number of days sedentary/screen time ≥ two hours**	**0**	5.2% (144)	6.1% (262)	5.5% (31)	5.8% (375)
	**1-2 days**	24.2% (674)	23.5% (1,011)	24.8% (141)	23.8% (1,544)
	**3-4 days**	21.7% (599)	20.6% (886)	21.1% (120)	21.0% (1,365)
	**5-6 days**	14.0% (386)	13.8% (593)	13.9% (79)	13.9% (900)
	**7 days**	34.8% (961)	36.0% (1,546)	34.8% (198)	35.6% (2,309)
	Missing	*0%*	*0%*	*0%*	*0%*
**Number of days tired**	**0**	21.0% (582)	21.0% (903)	19.2% (109)	21.2% (1,376)
	**1-2 days**	32.4% (895)	32.0% (1,377)	35.7% (203)	31.9% (2,069)
	**3-4 days**	17.6% (487)	17.5% (754)	18.8% (107)	17.5% (1,134)
	**5-6 days**	10.0% (276)	9.3% (399)	10.5% (60)	9.5% (615)
	**7 days**	19.0% (524)	20.1% (865)	15.8% (90)	20.0% (1,299)
	Missing	*0%*	*0%*	*0%*	*0%*
**Number of days sugary snack**	**0**	6.5% (179)	7.7% (332)	6.3% (36)	7.3% (475)
	**1-2 days**	34.9% (964)	32.7% (1,407)	35.0% (199)	33.5% (2,172)
	**3-4 days**	25.3% (698)	26.7% (1,146)	25.1% (143)	26.2% (1,701)
	**5-6 days**	13.4% (371)	12.0% (515)	15.3% (87)	12.3% (799)
	**7 days**	20.0% (552)	20.9% (898)	18.3% (104)	20.7% (1,346)

	Missing	*0%*	*0%*	*0%*	*0%*
		**General**			
**Number of out of school clubs**	**0**	27.7% (766)	32.3% (1,387)	25.1% (143)	31.0% (2,010)
	**1**	17.9% (495)	16.9% (726)	16.0% (91)	17.4% (1,130)
	**2**	16.0% (443)	15.1% (650)	14.9% (85)	15.5% (1,008)
	**3**	11.1% (308)	10.4% (446)	13.3% (76)	10.4% (678)
	**4**	7.4% (204)	7.3% (313)	7.6% (43)	7.3% (474)
	**5**	6.2% (171)	5.8% (251)	5.8% (33)	6.0% (389)
	**6**	3.4% (95)	2.5% (109)	5.1% (29)	2.7% (175)
	**7**	3.3% (91)	2.5% (107)	5.1% (29)	2.6% (169)
	**8**	1.1% (29)	0.8% (33)	1.8% (10)	0.8% (52)
	**9**	0.9% (24)	0.7% (32)	1.2% (7)	0.8% (49)
	**10**	3.9% (107)	4.0% (174)	3.3% (19)	4.0% (262)
	Missing	*1.1% (31)*	*1.6% (70)*	*0.7% (<5)*	*1.5% (97)*
**Can ride a bike**	**Yes**	88.8% (2,444)	86.0% (3,696)	91.4% (520)	86.7% (5,641)
	**No**	11.2% (309)	14.0% (602)	8.6% (49)	13.3% (862)
	Missing	*0%*	*0%*	*0%*	*0%*
**Can swim 25m**	**Yes**	78.9% (2,180)	72.9% (3,134)	80.3% (457)	74.8% (4,857)
	**No**	21.1% (584)	27.1% (1,164)	19.7% (112)	25.2% (1,636)
	Missing	*0%*	*0%*	*0%*	*0%*
