# Supplementary material for: Pre-COVID-19 pandemic health-related behaviours in children (2018–2020) and association with being tested for SARS-CoV-2 and testing positive for SARS-CoV-2 (2020–2021): a retrospective cohort study using survey data linked with routine health data in Wales, UK
Source: BMJ Open. 2022 Sep 7;12(9):e061344. doi: 10.1136/bmjopen-2022-061344 (PMC9453425; doi:10.1136/bmjopen-2022-061344)
Supplement: Supplementary data [file bmjopen-2022-061344supp006.pdf]

Online supplemental appendix 6:
Multivariable logistic regression model of health behaviour markers and probability of PCR-test without confounders.

PCR tested for SARS-CoV-2 (n=6,958, R^2=0.01)	OR	*p* value	95% CI
**Ate breakfast**	1.05	0.632	0.87 – 1.27
Reference: did not eat breakfast	1.00		
**Active travel to school**	0.92	0.238	0.80 – 1.06
Reference: did not active travel to school	1.00		
**Active travel from school**	1.08	0.273	0.94 – 1.24
Reference: did not active travel from school	1.00		
**Number of fruit/vegetable portions**	1.00	0.941	0.98 – 1.03
**Number of times teeth brushed**	0.97	0.474	0.90 – 1.05
**Sleep hours**	0.99	0.654	0.96 – 1.02
Reference: 0 days physically active ≥60 mins (previous seven days)	1.00		
**1-2 days physically active ≥ 60 mins**	1.12	0.276	0.91 – 1.38
**3-4 days physically active ≥ 60 mins**	1.14	0.221	0.92 – 1.42
**5-6 days physically active ≥ 60 mins**	1.17	0.177	0.93 – 1.47
**7 days physically active ≥ 60 mins**	1.09	0.475	0.87 – 1.37
Reference: 0 days sedentary ≥two hours (previous seven days)	1.00		
**1-2 days sedentary ≥ two hours**	1.16	0.209	0.92 – 1.46
**3-4 days sedentary ≥ two hours**	1.18	0.166	0.93 – 1.49
**5-6 days sedentary ≥ two hours**	1.15	0.275	0.90 – 1.47
**7 days sedentary ≥ two hours**	1.14	0.256	0.91 – 1.44
Reference: 0 days felt tired (previous seven days)	1.00		
**1-2 days felt tired**	0.98	0.791	0.86 – 1.13
**3-4 days felt tired**	0.99	0.881	0.84 – 1.16
**5-6 days felt tired**	1.04	0.667	0.86 – 1.26
**7 days felt tired**	0.97	0.730	0.83 – 1.14
Reference: 0 days consumed sugary snack (previous seven days)			
**1-2 days consumed sugary snack**	1.21*	0.062	0.99 – 1.49
**3-4 days consumed sugary snack**	1.08	0.489	0.87 – 1.33
**5-6 days consumed sugary snack**	1.29**	0.034	1.02 – 1.63
**7 days consumed sugary snack**	1.12	0.314	0.90 – 1.39
**Number of out of school clubs participation**	1.02	0.121	1.00 to 1.04
**Can ride a bike**	1.16*	0.064	0.99 – 1.35
Reference: cannot ride a bike	1.00		
**Can swim 25m**	1.30**	< 0.001	1.15 – 1.46

Reference: cannot swim 25m	1.00

OR: Odds Ratio; 95% CI: 95% confidence intervals; p<0.05**, p<0.1*. See online supplemental appendix 4 for variable codebook.

Multivariable logistic regression model of health behaviour markers and probability of PCR-test positive without confounders.

**PCR test positive for SARS-CoV-2 (n=6,958, R^2=0.01)**	**OR**	***p* value**	**95% CI**
**Ate breakfast**	1.30	0.170	0.89 – 1.91
Reference: did not eat breakfast	1.00		
**Active travel to school**	0.91	0.451	0.71 – 1.17
Reference: did not active travel to school	1.00		
**Active travel from school**	1.07	0.614	0.83 – 1.36
Reference: did not active travel from school	1.00		
**Number of fruit/vegetable portions**	0.99	0.574	0.94 – 1.03
Reference: 0 fruit/vegetable portions	1.00		
**Number of times teeth brushed**	1.07	0.385	0.92 – 1.24
Reference: did not brush teeth	1.00		
**Sleep hours**	0.97	0.266	0.92 – 1.02
Reference: 0 days physically active ≥60 mins (previous seven days)	1.00		
**1-2 days physically active ≥ 60 mins**	1.71	0.023	1.08 – 2.73
**3-4 days physically active ≥ 60 mins**	1.87	0.009	1.17 – 2.99
**5-6 days physically active ≥ 60 mins**	1.61	0.059	0.98 – 2.63
**7 days physically active ≥ 60 mins**	1.49	0.117	0.91 – 2.43
Reference: 0 days sedentary ≥two hours (previous seven days)	1.00		
**1-2 days sedentary ≥ two hours**	1.03	0.877	0.68 – 1.57
**3-4 days sedentary ≥ two hours**	1.00	0.983	0.66 – 1.54
**5-6 days sedentary ≥ two hours**	1.01	0.958	0.65 – 1.59
**7 days sedentary ≥ two hours**	1.10	0.660	0.72 – 1.66
Reference: 0 days felt tired (previous seven days)	1.00		
**1-2 days felt tired**	1.21	0.125	0.95 – 1.55
**3-4 days felt tired**	1.17	0.278	0.88 – 1.55
**5-6 days felt tired**	1.21	0.273	0.86 – 1.69
**7 days felt tired**	0.92	0.600	0.69 – 1.24
Reference: 0 days consumed sugary snack (previous seven days)	1.00		
**1-2 days consumed sugary snack**	1.14	0.499	0.78 – 1.67
**3-4 days consumed sugary snack**	1.03	0.873	0.70 – 1.53
**5-6 days consumed sugary snack**	1.38	0.131	0.91 – 2.11
**7 days consumed sugary snack**	1.04	0.867	0.69 – 1.56
**Number of out of school clubs participation**	1.05	0.007	1.01 – 1.09
**Can ride a bike**	1.40	0.032	1.03 – 1.92

Reference: cannot ride a bike	1.00		
**Can swim 25m**	1.16	0.207	0.92 – 1.45
Reference: cannot swim 25m	1.00		
