# Supplementary material for: Pre-COVID-19 pandemic health-related behaviours in children (2018–2020) and association with being tested for SARS-CoV-2 and testing positive for SARS-CoV-2 (2020–2021): a retrospective cohort study using survey data linked with routine health data in Wales, UK
Source: BMJ Open. 2022 Sep 7;12(9):e061344. doi: 10.1136/bmjopen-2022-061344 (PMC9453425; doi:10.1136/bmjopen-2022-061344)
Supplement: Author's manuscript [file bmjopen-2022-061344.draft_revisions.pdf]

BMJ Open is committed to open peer review. As part of this commitment we make the peer review history of every article we publish publicly available.

When an article is published we post the peer reviewers' comments and the authors' responses online. We also post the versions of the paper that were used during peer review. These are the versions that the peer review comments apply to.

The versions of the paper that follow are the versions that were submitted during the peer review process. They are not the versions of record or the final published versions. They should not be cited or distributed as the published version of this manuscript.

BMJ Open is an open access journal and the full, final, typeset and author-corrected version of record of the manuscript is available on our site with no access controls, subscription charges or pay-per-view fees (http://bmjopen.bmj.com).

If you have any questions on BMJ Open's open peer review process please email info.bmjopen@bmj.com

BMJ Open

Prior health-related behaviours in children (2014-2020) and association with a positive SARS-CoV-2 test during adolescence (2020-2021): a retrospective cohort study using survey data linked with routine health data in Wales, UK

Journal:	*BMJ Open*
Manuscript ID	bmjopen-2022-061344
Article Type:	Original research
Date Submitted by the Author:	25-Jan-2022

Complete List of Authors:	Marchant, Emily ; Swansea University, Population Data Science, Medical School; Swansea University, National Centre for Population Health and Wellbeing Research<br>Lowthian, Emily; Swansea University, Population Data Science, Medical School<br>Crick, Tom; Swansea University, Department of Education & Childhood Studies<br>Griffiths, Lucy ; Swansea University, Population Data Science, Medical School<br>Fry, Richard; Swansea University, Medical School; Swansea University, National Centre for Population Health and Wellbeing Research<br>Dadaczynski, Kevin; Fulda University of Applied Sciences, Department of Nursing and Health Science; Leuphana University Luneburg Center of Applied Health Sciences<br>Okan, Orkan; Technical University of Munich<br>James, M; Swansea University, Population Data Science, Medical School; Swansea University, National Centre for Population Health and Wellbeing Research<br>Cowley, Laura; Public Health Wales, Research and Evaluation Division<br>Torabi, Fatemeh; Swansea University Medical School, Population Data Science<br>Kennedy, Jonathan; Swansea University, Population Data Science, Medical School; Swansea University, National Centre for Population Health and Wellbeing Research<br>Akbari, Ashley; Swansea University, Population Data Science, Medical School<br>Lyons, Ronan; Swansea University, Population Data Science, Medical School<br>Brophy, Sinead; Swansea University, Population Data Science, Medical School; Swansea University, National Centre for Population Health and Wellbeing Research

Keywords:	Community child health < PAEDIATRICS, Epidemiology < INFECTIOUS DISEASES, PUBLIC HEALTH, Public health < INFECTIOUS DISEASES

SCHOLARONE™
Manuscripts

I, the Submitting Author has the right to grant and does grant on behalf of all authors of the Work (as defined in the below author licence), an exclusive licence and/or a non-exclusive licence for contributions from authors who are: i) UK Crown employees; ii) where BMJ has agreed a CC-BY licence shall apply, and/or iii) in accordance with the terms applicable for US Federal Government officers or employees acting as part of their official duties; on a worldwide, perpetual, irrevocable, royalty-free basis to BMJ Publishing Group Ltd ("BMJ") its licensees and where the relevant Journal is co-owned by BMJ to the co-owners of the Journal, to publish the Work in this journal and any other BMJ products and to exploit all rights, as set out in our licence.

The Submitting Author accepts and understands that any supply made under these terms is made by BMJ to the Submitting Author unless you are acting as an employee on behalf of your employer or a postgraduate student of an affiliated institution which is paying any applicable article publishing charge ("APC") for Open Access articles. Where the Submitting Author wishes to make the Work available on an Open Access basis (and intends to pay the relevant APC), the terms of reuse of such Open Access shall be governed by a Creative Commons licence – details of these licences and which Creative Commons licence will apply to this Work are set out in our licence referred to above.

Other than as permitted in any relevant BMJ Author's Self Archiving Policies, I confirm this Work has not been accepted for publication elsewhere, is not being considered for publication elsewhere and does not duplicate material already published. I confirm all authors consent to publication of this Work and authorise the granting of this licence.

For peer review only

Prior health-related behaviours in children (2014-2020) and association with a positive SARS-CoV-2 test during adolescence (2020-2021): a retrospective cohort study using survey data linked with routine health data in Wales, UK

Emily Marchant[1,2*], Emily Lowthian[1], Tom Crick[3], Lucy Griffiths[1], Richard Fry[1,2], Kevin Dadaczynski[4,5], Orkan Okan[6], Michaela James[1,2], Laura Cowley[2,7] Fatemeh Torabi[1], Jonathan Kennedy[1,2], Ashley Akbari[1], Ronan Lyons[1], Sinead Brophy[1,2]

[1] Population Data Science, Medical School, Swansea University, Swansea, SA2 8PP, United Kingdom

[2] National Centre for Population Health and Wellbeing Research, Swansea University, Swansea, SA2 8PP, United Kingdom

[3] Department of Education & Childhood Studies, Swansea University, Swansea, SA2 8PP, United Kingdom

[4] Department of Nursing and Health Science, Fulda University of Applied Sciences, Fulda, 36037, Germany

[5] Center for Applied Health Science, Leuphana University Lueneburg, Lueneburg, 21335, Germany

[6] Chair Health Literacy, Department of Sport and Health Sciences, Technical University Munich, Uptown München-Campus D, 4. OG, OfficeL412, Georg-Brauchle-Ring 60/62, 80992 München, Germany

[7] Research and Evaluation Division, Public Health Knowledge and Research Directorate, Public Health Wales, 2 Capital Quarter, Cardiff, CF10 4BZ, United Kingdom

[*] Corresponding author: Emily Marchant [1,2], email: E.K.Marchant@swansea.ac.uk

Word count: 3,809

For peer review only

ABSTRACT

Objectives

Examine if prior health-related behaviours during primary school are associated with being tested for SARS-CoV-2 and testing positive during adolescence.

Design

Retrospective cohort study using an online cohort survey (1 April 2014 to 28 February 2020) linked to routine PCR SARS-CoV-2 test results (1 March 2020 to 31 August 2021)

Setting

Children attending primary schools in Wales (2014-2020), UK who were part of the HAPPEN schools network.

Participants

Complete linked records of eligible participants were obtained for n=6,891 individuals. 43.2% (n=3,021) were tested (baseline age 12.3±2.0, 48% boys) and 11.2% (n=774) tested positive for SARS-CoV-2 (baseline age 12.8±2.1, 43.9% boys).

Main outcome measures

Logistic regression of health-related behaviours and sex, age, deprivation, clustered by school was used to determine Odds Ratios (OR) of factors associated with being tested for or testing positive for SARS-CoV-2.

For peer review only - http://bmjopen.bmj.com/site/about/guidelines.xhtml

Results

Sleeping 9+ hours (OR=1.15, 95% CI 1.01 to 1.29), participating in 3+ out of school clubs (OR=1.15, 95% CI 1.02 to 1.31), able to swim (OR=1.29, 95% CI 1.10 to 1.52) and ride a bike (OR=1.16, 95% CI 0.98 to 1.37, p<0.1) were associated with being tested for SARS-CoV-2. Participating in 3+ out of school clubs (OR=1.12, 95% CI 1.02 to 1.56), able to ride a bike (OR=1.36, 95% CI 0.97 to 1.92, p<0.1), sex (girl; OR=1.25, 95% CI 1.06 to 1.47) and baseline age (OR=1.16, 95% CI 1.10 to 1.22) were associated with an increased likelihood of testing positive (OR=1.16, 95% CI 1.10 to 1.22).

Conclusions

Actions associated with a child being PCR-tested and identified as positive may be related to parental health literacy e.g. parents recognising symptoms, knowledge of testing services. Identification of adolescent positive cases may be highly skewed towards children whose parents have higher health literacy. As those not accessing testing services remain undetected true rates of COVID-19 are not known in adolescence.

For peer review only

STRENGTHS AND LIMITATIONS

- First study to investigate association of prior child health-related behaviour measures with subsequent SARS-CoV-2 testing and infection during adolescence.
- Reporting of multiple child health behaviours linked at an individual-level to routine records of SARS-CoV-2 testing data through the SAIL Databank.

For peer review only - http://bmjopen.bmj.com/site/about/guidelines.xhtml

- Child-reported health behaviours were measured before the COVID-19 pandemic and represent historical health behaviour which may not reflect behaviours during COVID-19.
- Health behaviours captured through the national-scale HAPPEN survey represent children attending schools that engaged with the HAPPEN Wales primary school network which may not be representative of the whole population of Wales.
- The period of study for PCR-testing for and testing positive for SARS-CoV-2 includes a time frame with varying prevalence rates, different variants, approaches to testing children, public health measures and restrictions which were not measured in this study.

For peer review only - http://bmjopen.bmj.com/site/about/guidelines.xhtml

BACKGROUND

The coronavirus disease 2019 (COVID-19) pandemic caused by severe acute respiratory syndrome coronavirus 2 (SARS-CoV-2) has resulted in widespread disruption to the lives of children across the world, defined by the *Convention on the Rights of the Child* as a person under the age of 18 years[1]. This has impacted on their education and health-related behaviours such as nutrition and physical activity[2]. While a growing body of literature suggests children display fewer clinical symptoms[3], the COVID-19 pandemic has contributed to widened inequalities in children's health, wellbeing and education[4,5]. Positive SARS-CoV-2 tests require periods of self-isolation, impacting children's physical health and wellbeing, limiting opportunities for children to engage in health-promoting behaviours such as regular physical activity[6].

Childhood is a critical developmental period during which healthy habits are formed which transcend into adolescence, recognised by the World Health Organization as those aged between 10 and 19 years (early; 10-14 years, middle; 15-17 years, late; 18+), and into adulthood[7]. It is important to minimise the risk of SARS-CoV-2 transmission in children and adolescents to prevent further exacerbation of pre-existing inequalities and safeguard their health, wellbeing and education, alongside reducing wider societal transmission.

Evidence has demonstrated the negative impact of the COVID-19 pandemic on children's health-related behaviours including reduced physical activity, increased sedentary behaviour and poorer nutrition[4,6]. However, it is unclear if this association is bidirectional, that is, whether these health behaviours may also be associated with risk of SARS-CoV-2 infection. Evidence suggests a plausible relationship between health risk behaviours such as

For peer review only - http://bmjopen.bmj.com/site/about/guidelines.xhtml

physical inactivity, poor nutrition and inadequate sleep with SARS-CoV-2 infection and severity of disease, attributed to immune system function and cardiometabolic health[8–11]. However, the focus of research to date has been adult populations, exploring single health behaviours or examining those with severe COVID-19 infection and hospitalisation[12,13].

Profiling research within the childhood population has generally centred on identifying the clinical characterisation of infection, with further attention to those with serious infection requiring hospital admission[14,15]. Whilst serious COVID-19 illness in children is relatively rare, mild or asymptomatic infection is common[16]. Furthermore, while there is a rollout of vaccination programmes throughout the adult population in the UK, the vaccination programme for 12–15-year-olds is currently in its early stages. Children below the age of 12 that are not clinically vulnerable are yet to be offered the first dose of vaccination in the UK as of January 2022.

Identifying the prior health-related behavioural characteristics of children and adolescents subsequently requiring a SARS-CoV-2 test or testing positive for SARS-CoV-2 infection could yield insight into the clustering of health behaviours during childhood and adolescence and subsequent infection risk during the current COVID-19 pandemic and future pandemic/endemic scenarios. This can also allow targeted intervention to minimise transmission risk that complements national public health measures and guidelines, and importantly, mitigate the disruption to children's lives. In Wales, one of the four nations of the UK, approaches to performing PCR tests on children include the presence of COVID-19 symptoms, if identified as a close contact to a positive case (e.g. household contacts), or following a positive lateral flow test (e.g. showing symptoms and having a positive lateral flow test performed in the home).

For peer review only - http://bmjopen.bmj.com/site/about/guidelines.xhtml

This study investigates the association of prior health-related behaviours self-reported by children aged 8-11 years during primary school before the COVID-19 pandemic between 1 April 2014 and 28 February 2020, with the odds of having a test and testing positive for SARS-CoV-2 during adolescence (aged 10-19). We aim to examine whether these self-reported markers of health-related behaviours during primary school are associated with likelihood of; i) being tested for SARS-CoV-2 (e.g. presence of symptoms) and ii) testing positive for SARS-CoV-2 during the adolescent period (aged 10-19 years), between 1 March 2020 and 31 August 2021.

METHODS

Study design

This study was conducted through the HAPPEN primary school network (*Health and Attainment of Pupils in a Primary Education Network*)[17]. HAPPEN was established in Wales, UK in 2014, following research with headteachers who advocated for increased collaboration to prioritise pupils' health and wellbeing[18,19]. The network brings together primary schools with research and runs up to the current date. School participation in HAPPEN is voluntary and is either once, annually or bi-annually (e.g. to evaluate school-based interventions). Through HAPPEN, children aged 8-11 (years 4 to 6) complete the HAPPEN survey, an online cohort survey that captures a range of validated self-reported health behaviours including physical activity, nutrition and sleep[20]. Retrospective health-related behaviour data were obtained from responses from the HAPPEN survey between 1 April 2014 and 28 February 2020. These retrospective survey responses were linked with polymerase chain reaction (PCR)

For peer review only - http://bmjopen.bmj.com/site/about/guidelines.xhtml

For peer review only

SARS-CoV-2 test results obtained from the Pathology COVID-19 Daily (PATD) routine dataset between 1 March 2020 and 31 August 2021. Linkage was performed using the SAIL (*Secure Anonymised Information Linkage*) Databank[21–23]. Data were linked at the individual level using an Anonymous Linkage Field (ALF) to identify participants and link SARS-CoV-2 test results (figure 1). The RECORD checklist[24] for this study is presented in online supplemental appendix 1.

Ethics

Ethical approval was granted by the Swansea University Medical School Research Ethics Committee (2017-0033H). Electronic data (survey responses) were stored in password-protected files only accessible to the research team. The routine data used in this study are available in the SAIL Databank and are subject to review by an independent Information Governance Review Panel (IGRP), to ensure proper and appropriate use of SAIL data. Before any data can be accessed, approval must be received from the IGRP. When access has been approved, it is accessed through a privacy-protecting safe haven and remote access system referred to as the SAIL Gateway. SAIL has established an application process to be followed by anyone who would like to access data. This study has been approved by the SAIL IGRP (project reference: 0911).

The HAPPEN survey and linked SAIL data

Primary schools in Wales, UK were invited to participate in the HAPPEN survey between 1 April 2014 and 28 February 2020 via a number of methods including email, social

For peer review only - http://bmjopen.bmj.com/site/about/guidelines.xhtml

media promotion and through stakeholders in health and education (including local authority health and wellbeing teams, regional education consortia). Schools were invited to share details of the survey with parents/guardians (including information sheets). To participate in the HAPPEN survey and link data to routine records, child assent was required in addition to parental consent (between 2014 to 2018) and opt-out parental consent (2019 onwards).

The HAPPEN survey is completed by children aged 8-11 as a self-guided activity within the school setting as a classroom activity with supervision from a teacher/teaching assistant. The survey takes approximately 30 minutes to complete and includes validated self-report measures of typical health behaviours including physical activity, screen time, nutrition, sleep and wellbeing[20]. A full copy of the survey can be found in online supplemental appendix 2 and items, response categories and the coding framework included within analyses in online supplemental appendix 3.

The process of data coding involved two researchers. The first (MJ) cleaned the raw data (including checking for duplicate entries), removed identifiable information and generated a unique participant ID number to protect participants' anonymity. The second (EM) researcher coded the anonymised raw dataset using STATA (version 16) to produce a dataset for analyses. This HAPPEN dataset was uploaded to the SAIL Databank, a trusted research environment (TRE) containing individual-level anonymised population-scale data sources about the population of Wales that enables secure data linkage and analysis for research, to be linked with SARS-CoV-2 testing data from the PATD dataset. To link the data, the person-based identifiable data are separated from the survey data and sent to a trusted third party, Digital Health and Care Wales (the national organisation that designs and builds digital services for health and social care in Wales). The survey data is sent to SAIL using a secure file upload. A unique Anonymous Linking Field (ALF) is assigned to the person-based

For peer review only - http://bmjopen.bmj.com/site/about/guidelines.xhtml

record before it is joined to clinical data via a system linking field. The ALF was used to link records at the individual level between the HAPPEN dataset and PATD dataset containing PCR testing data. This dataset was accessible to authors listed from the Population Data Science group, Swansea University.

Quantitative analysis

The primary outcomes were i) whether the child was PCR tested for the SARS-CoV-2 virus and ii) whether the child had a positive SARS-CoV-2 test between 1 March 2020 and 31 August 2021. Eligibility criteria (see cohort flow diagram, Figure 1) within final analyses models were any unique participant with complete linked survey and routine records, aged at least 10 years on 1 March 2020 (start of period of interest) (n=6,891). Inclusion dates of survey responses for analyses were between 1 April 2014 and 28 February 2020. Logistic regression analyses, adjusting for confounding variables (sex, age on 1 March 2020, area-level deprivation using the Welsh Index of Multiple Deprivation (WIMD)[25] (version 2019) and clustered by school (to account for differences between schools), determined Odds Ratios (OR) for i) being PCR tested for SARS-CoV-2 virus (1; tested at least once for SARS-CoV-2 between 1 March 2020 and 31 August 2021, 0; no evidence of SARS-CoV-2 test during period of interest) and ii) having a positive PCR SARS-CoV-2 test (1; testing positive for SARS-CoV-2 between 1 March 2020 and 31 August 2021, 0; testing negative for SARS-CoV-2, 0; no evidence of PCR test).

Independent variables as measures of typical health-related behaviours included within analyses were obtained retrospectively from the HAPPEN survey, completed between 1 April 2014 and 28 February 2020 (online supplemental appendix 3). Health-related

For peer review only - http://bmjopen.bmj.com/site/about/guidelines.xhtml

behaviour measures included in multivariable analyses related to the behaviours from the previous day (ate breakfast, travel actively to and/or from school, ate at least five portions of fruit/vegetables, twice-daily toothbrushing, slept at least nine hours), behaviours every day the previous seven days (physically active at least 60 minutes, sedentary/screen time at least two hours, felt tired, ate a sugary snack), participate in at least three out of school clubs, can ride a bike and can swim 25 metres[20]. For the purpose of analyses, survey items with multiple category responses (e.g. active travel to school response categories: walked, on bike, ran/jogged, scooter, skateboard/rollerblade assigned as binary active travel) or continuous numerical values (e.g. out of school clubs assigned as binary value indicating participation in at least three clubs) were assigned binary values. A list of variables included in analyses, coding response categories and a coding framework is presented in online supplemental appendix 3. Independent variables were first entered concurrently and examined for association with outcomes. Then a process of backward stepwise selection was manually followed to build the final regression models. This involved the inclusion of all variables within the initial model, followed by the individual removal of the least significant variables until no nonsignificant variables at the 10% level remained within the model.

Figure 1: Cohort flow diagram

Patient and public involvement

The SAIL Databank has a Consumer Panel that provides the public's perspective on data linkage research. The Panel members are involved in all elements of the SAIL Databank

For peer review only - http://bmjopen.bmj.com/site/about/guidelines.xhtml

process, from developing ideas, advising on bids through approval processes (via the independent Information Governance Review Panel), to disseminating research findings. For more information visit https://saildatabank.com/about-us/public-engagement/.

For peer review only

For peer review only - http://bmjopen.bmj.com/site/about/guidelines.xhtml

RESULTS

Survey responses were obtained from n=11,339 participants (figure 1), of which n=1,101 were duplicate participants (occasions of multiple survey entries). In the case of duplicates, the first instance of survey participation was used and later responses excluded to create a dataset consisting of unique participants (n=10,238). Inclusion dates of survey responses for analyses were between 1 April 2014 and 28 February 2020. Participants with survey responses outside the period of interest were excluded (n=248 excluded, March 2020). Age criteria within final analyses was aged at least 10 years on 1 March 2020 (n=3,099 excluded, aged <10 years). Complete linked unique records of participants meeting eligibility criteria were obtained for n=6,891 individuals. Of the total sample, 43.2% (n=3,021) were PCR-tested for SARS-CoV-2 (table 1) and 11.2% (n=774) tested positive for SARS-CoV-2 (table 2). The mean age on 1 March 2020 (start of period of interest) was 12.3 (±2.0) for those PCR-tested (table 1) and 12.8 (±2.1) for those who tested positive for SARS-CoV-2 (table 2). As the adolescence period spans a range of ages, we have presented a breakdown of distribution of developmental stage on 1 March 2020 for testing for SARS-CoV-2 and testing positive for SARS-CoV-2 in table 1 and table 2 respectively. Of the total sample, 86% (n=5,927) were aged between 10-14 (early adolescence) 14% aged 15-17 (middle adolescence) and 0% in late adolescence. Complete case analyses are presented.

Table 1: Descriptive statistics of study sample by PCR-tested for SARS-CoV-2.

	**Tested for SARS-CoV-2 n (%)**	**Not tested for SARS-CoV-2 n (%)**
Sample	43.8% (3,021)	56.2% (3,870)

For peer review only - http://bmjopen.bmj.com/site/about/guidelines.xhtml

Age at the time HAPPEN survey		10.5 ± 0.6	10.5 ± 0.6
Age on 1 March 2020 (start of period of interest)		12.3 ± 2.0	12.2 ± 1.9
Age on 1 March 2020 (start of period of interest)		12.3 ± 2.0	12.2 ± 1.9
	**Early adolescence (10-14 years)**	85.1% (2,570)	86.7% (3,357)
	**Mid adolescence (15-17 years)**	14.9% (451)	13.3% (513)
	**Late adolescence (18+ years)**	0	0
Sex	**Boy**	48.0% (1,449)	48.4% (1,871)
	**Girl**	50.9% (1,537)	50.9% (1,970)
	Missing	*1.2% (35)*	*0.8% (29)*
WIMD version 2019 quintiles	**1** (most deprived)	22.3% (675)	23.8% (919)
	**2**	16.2% (488)	15.9% (614)
	**3**	16.1% (487)	17.5% (678)
	**4**	12.5% (378)	11.6% (448)
	**5** (least deprived)	21.7% (655)	19.9% (770)
	Missing	*11.2% (338)*	*11.4% (441)*
	**Previous day**		
Ate breakfast	**Yes**	91.0% (2,750)	90.1% (3,520)
	**No**	7.5% (227)	7.6% (292)
	Missing	*1.5% (44)*	*1.5% (58)*
Active travel to school	**Yes**	39.5% (1,194)	41.8% (1,617)
	**No**	58.9% (1,779)	56.7% (2,194)
	Missing	*1.6% (48)*	*1.5% (59)*
Active travel from school	**Yes**	44.7% (1,349)	45.8% (1,771)
	**No**	53.8% (1,626)	52.7% (2,040)
	Missing	*1.5% (46)*	*1.5% (59)*
Toothbrush ≥ two times	**Yes**	79.3% (2,396)	78.2% (3,025)
	**No**	19.1% (577)	20.2% (782)
	Missing	*1.6% (48)*	*1.6% (63)*
Consumed ≥ five fruit & veg	**Yes**	23.7% (717)	24.0% (930)
	**No**	74.8% (2,261)	74.4% (1.6%)
	Missing	*1.4% (43)*	*1.6% (61)*
Sleep ≥ nine hours	**Yes**	78.0% (2,357)	76.6% (2,963)
	**No**	19.8% (599)	21.4% (829)
	Missing	*2.2% (65)*	*2.0% (78)*

For peer review only - http://bmjopen.bmj.com/site/about/guidelines.xhtml

**Previous 7 days**			
Physically active ≥60 mins every day	**Yes**	21.2% (640)	20.8% (805)
	**No**	77.3% (2,335)	77.7% (3,008)
	Missing	*1.5% (46)*	*1.5% (57)*
Sedentary/screen time ≥2 hours every day	**Yes**	30.5% (922)	32.4% (1,252)
	**No**	68.0% (2,053)	66.2% (2,561)
	Missing	*1.5% (46)*	*1.5% (57)*
Tired every day	**Yes**	15.2% (460)	15.1% (584)
	**No**	83.3% (2,515)	83.4% (3,229)
	Missing	*1.5% (46)*	*1.5% (57)*
Sugary snack every day	**Yes**	18.0% (545)	18.4% (710)
	**No**	80.4% (2,430)	80.2% (3,103)
	Missing	*1.5% (46)*	*1.5% (57)*
**General**			
Participate in ≥ three out of school clubs per week	**Yes**	35.9% (1,083)	32.0% (1,239)
	**No**	57.2% (1,729)	61.6% (2,382)
	Missing	*6.9% (209)*	*6.4% (249)*
Can ride a bike	**Yes**	89.7% (2,711)	87.7% (3,394)
	**No**	8.5% (257)	10.6% (409)
	Missing	*1.8% (53)*	*1.7% (67)*
Can swim 25m	**Yes**	81.0% (2,446)	76.4% (2,955)
	**No**	17.3% (523)	22.0% (853)
	Missing	*1.7% (52)*	*1.6% (62)*

See online supplemental appendix 3 for variable codebook.

Table 2: Descriptive statistics of study sample by PCR-tested positive for SARS-CoV-2.

		**Tested positive for SARS-CoV-2 n (%)**	**No evidence of positive SARS-CoV-2 test n (%)**
Sample		11.2% (774)	88.8% (6,117)
Age at the time HAPPEN survey		10.5 ± 0.7	10.5 ± 0.6
Age on 1 March 2020 (start of period of interest)		12.8 ± 2.1	12.2 ± 1.9
Sex	**Boy**	43.9% (340)	48.7% (2,980)
	**Girl**	55.6% (430)	50.3% (3,077)
	Missing	*0.5% (<5)*	*1.0% (60)*
WIMD version 2019 quintiles	**1** (most deprived)	25.7% (199)	22.8% (1,395)
	**2**	13.7% (106)	16.3% (996)
	**3**	18.7% (145)	16.7% (1,020)
	**4**	9.8% (76)	12.3% (750)

For peer review only - http://bmjopen.bmj.com/site/about/guidelines.xhtml

For peer review only

	**5** (least deprived)	22.0% (170)	20.5% (1,255)
	Missing	*10.1% (78)*	*11.5% (701)*
	**Previous day**		
Ate breakfast	**Yes**	91.5% (708)	90.9% (5,562)
	**No**	6.7% (52)	7.6% (467)
	Missing	*1.8% (14)*	*1.4% (88)*
Active travel to school	**Yes**	37.9% (293)	41.2% (2,518)
	**No**	60.2% (466)	57.3% (3,07)
	Missing	*1.9% (15)*	*1.5% (92)*
Active travel from school	**Yes**	43.7% (338)	45.5% (2,782)
	**No**	54.3% (420)	53.1% (3,246)
	Missing	*2.1% (16)*	*1.5% (89)*
Toothbrush ≥two times	**Yes**	80.5% (623)	78.4% (4,798)
	**No**	17.6% (136)	20.0% (1,223)
	Missing	*1.9% (15)*	*1.6% (96)*
Consumed ≥five fruit & veg	**Yes**	26.2% (203)	23.6% (1,444)
	**No**	72.0% (557)	74.9% (4,583)
	Missing	*1.8% (14)*	*1.5% (90)*
Sleep ≥nine hours	**Yes**	78.4% (607)	77.1% (4,713)
	**No**	18.6% (144)	21.0 (1,284)
	Missing	*3.0% (23)*	*2.0% (120)*
	**Previous 7 days**		
Physically active ≥60 mins every day	**Yes**	21.8% (169)	20.9% (1,276)
	**No**	76.1% (589)	77.7% (4,754)
	Missing	*2.1% (16)*	*1.4% (87)*
Sedentary/screen time ≥2 hours every day	**Yes**	30.5% (236)	31.7% (1,938)
	**No**	67.4% (522)	66.9% (4,092)
	Missing	*2.1% (16)*	*1.4% (87)*
Tired every day	**Yes**	12.4% (96)	15.5% (948)
	**No**	85.5% (662)	83.1% (5,082)
	Missing	*2.1% (16)*	*1.4% (87)*
Sugary snack every day	**Yes**	17.3% (134)	18.3% (1,121)
	**No**	80.6% (624)	80.3% (4,909)
	Missing	*2.1% (16)*	*1.4% (87)*
	**General**		
Participate in ≥ three out of school clubs per week	**Yes**	39.0% (302)	33.0% (2,020)
	**No**	53.1% (411)	60.5% (3,700)
	Missing	*7.9% (61)*	*6.5% (397)*
Can ride a bike	**Yes**	90.8% (703)	88.3% (5,402)
	**No**	6.9% (53)	10.0% (613)
	Missing	*2.3% (18)*	*1.7% (102)*
Can swim 25m	**Yes**	82.2% (636)	77.9% (4,765)
	**No**	15.5% (120)	20.5% (1,256)
	Missing	*2.3% (18)*	*1.6% (96)*

See online supplemental appendix 3 for variable codebook.

For peer review only - http://bmjopen.bmj.com/site/about/guidelines.xhtml

Children reporting to sleep at least nine hours (OR=1.15, 95% CI 1.01 to 1.29), participate in at least three out of school clubs (OR=1.15, 95% CI 1.02 to 1.31), able to ride a bike (10% significance level) and able to swim 25 metres (OR=1.29, 95% CI 1.10 to 1.52) showed an increased likelihood of being tested for SARS-CoV-2 (table 3). The model showed a low goodness-of-fit (R^2=0.006). See online supplemental appendix 4 for multivariable logistic regression model of health behaviour markers and probability of being PCR-testing for SARS-CoV-2.

Table 3: Backward stepwise logistic regression model of significant (p<0.1) health behaviour markers and probability of being PCR-tested for SARS-CoV-2 accounting for baseline age, sex and deprivation, and clustered by school.

**PCR-tested for SARS-CoV-2 (0; no, 1; yes)** ***Number of obs=5,581*** ***R^2=0.006***	**OR**	**P**	**95% CI**
Sleep ≥nine hours	1.15	0.028	1.01 to 1.29
Participate in ≥three out of school clubs per week	1.15	0.023	1.02 to 1.31
Can ride a bike	1.16	0.095	0.98 to 1.37
Can swim 25m	1.29	0.002	1.10 to 1.52
Age on 1 March 2020 (start of period of interest)	1.02	0.328	0.98 to 1.05
Sex (girl)	0.97	0.591	0.86 to 1.09
WIMD quintile 2	1.10	0.303	0.92 to 1.31
3	0.96	0.626	0.80 to 1.14
4	1.10	0.394	0.89 to 1.36
5 (least deprived)	1.04	0.577	0.90 to 1.22

OR: Odds Ratio; 95% CI: 95% confidence intervals. See online supplemental appendix 3 for variable codebook. Low correlation between variables (coefficients -0.10 to 0.21).

Children who participated in at least three out of school clubs (OR=1.12, 95% CI 1.02 to 1.56) and being able to ride a bike (OR=1.36, 95% CI 0.97 to 1.92, p<0.1) were associated with an increased likelihood of testing positive for SARS-CoV-2, whilst reporting to feel tired (OR=0.78, 95% CI 0.61 to 0.98) was associated with a reduced likelihood (table 4). Age on 1 March 2020 (OR=1.16, 95% CI 1.10 to 1.22) and sex (girl; OR=1.25, 95% CI 1.06 to 1.47) was associated

For peer review only - http://bmjopen.bmj.com/site/about/guidelines.xhtml

with an increased likelihood of testing positive (table 4). Those in WIMD quintile 2 (OR=0.80, 95% CI 0.62 to 1.03) and quintile 4 (OR=0.78, 95% C 0.58 to 1.04) were less likely ($p<0.1$) to test positive compared to quintile 1 (most deprived) (table 4). The model showed a low goodness-of-fit (R^2=0.02). There was very low correlation between independent variables in backward stepwise regression models (table 3 and table 4) with coefficient values ranging from -0.10 to 0.21 and -0.08 to 0.15 respectively. See online supplemental appendix 5 for multivariable logistic regression model of health behaviour markers and probability of PCR-testing positive for SARS-CoV-2.

Table 4: Backward stepwise logistic regression model of significant ($p<0.1$) health behaviour markers and probability PCR-testing positive for SARS-CoV-2 accounting for baseline age, sex and deprivation, and clustered by school.

**PCR-test positive for SARS-CoV-2 (0; no, 1; yes)**<br>***Number of obs=5,616***<br>***R^2=0.02***	**OR**	**P**	**95% CI**
Tired every day previous week	0.78	0.032	0.61 to 0.98
Participate in ≥ three out of school clubs per week	1.26	0.031	1.02 to 1.56
Can ride a bike	1.36	0.079	0.97 to 1.92
Age on 1 March 2020 (start of period of interest)	1.16	0.000	1.10 to 1.22
Sex (girl)	1.25	0.005	1.06 to 1.47
WIMD quintile 2	0.80	0.084	0.62 to 1.03
3	1.05	0.686	0.82 to 1.34
4	0.78	0.093	0.58 to 1.04
5 (least deprived)	0.86	0.193	0.68 to 1.08

OR: Odds Ratio; 95% CI: 95% confidence intervals. See online supplemental appendix 3 for variable codebook. Low correlation between variables (coefficients -0.08 to 0.15).

For peer review only - http://bmjopen.bmj.com/site/about/guidelines.xhtml

DISCUSSION

This study aims to examine whether markers of health-related behaviours reported by children during primary school between 1 April 2014 and 28 February 2020 is associated with the likelihood of being PCR-tested for SARS-CoV-2 (e.g. presence of symptoms) and testing positive between 1 March 2020 and 31 August 2021 during adolescence (10-19 years). This study did not find evidence that reporting positive health-related behaviours is associated with a reduced odds of being tested or testing positive for SARS-CoV-2. Findings suggest that reporting the recommended level of sleep (at least nine hours), participating in at least three out of school clubs, being able to ride a bike and being able to swim 25 metres were associated with an increased likelihood of being tested for SARS-CoV-2. Participating in at least three out of school clubs and being able to ride a were associated with an increased likelihood of testing positive for SARS-CoV-2, whilst reporting to feel tired every day was associated with a reduced likelihood of testing positive for SARS-CoV-2. Girls and older age were was associated with increased likelihood of testing positive for SARS-CoV-2. Those living in WIMD quintiles 2 and 4 were less likely to test positive compared to those living in the most deprived quintile (quintile 1) (10% significance level).

Detecting positive SARS-CoV-2 cases through testing and adhering to self-isolation is an important strategy in reducing community transmission[26]. The majority of children in this study (86%) were in the early adolescence stage (10-14) at the baseline date, with the remaining 14% in middle adolescence (15-17 years). The detection of child positive cases using routine PCR testing data in this study requires a parent/guardian to take the child for testing and thus relies on parental/caregiver influence and involvement. We find associations between child-reported health-related behaviours with both PCR-testing for SARS-CoV-2 and

For peer review only - http://bmjopen.bmj.com/site/about/guidelines.xhtml

testing positive for SARS-CoV-2. We theorise that parents who have higher levels of health literacy are more likely to take their child for a SARS-CoV-2 test and are then more likely to be detected as positive. Parenting is an important contributor to promoting positive health behaviours in children, and is represented by a constellation of attitudes, behaviours and values for the child. Indeed, monitoring behaviours occur and our study suggests that these actions associated with a parent taking a child for a SARS-CoV-2 test represent parental health literacy, for example through ensuring the child has a sleep routine[27]. The clustering of physically active behaviours represented by the association of being able to swim and ride a bike may represent underlying parental involvement and modelling behaviour, including involvement in leisure time activities, providing financial and transport provision to attend organised activities such as access to swimming lessons and the provision of equipment[28]. This may also have a socioeconomic component, building on the ideas of Bourdieu in terms of social capital, and access to classes and health enhancing material items[29].

The detection of positive child cases also relies on parents recognising symptoms, knowledge of how to access testing services, ability to access services (e.g. transport) and willingness to provide personal information for test and trace services. This is likely influenced by parental health literacy, recognised as the ability to access, understand, interpret and apply medical information and make informed decisions regarding medical advice, issues or guidelines[30]. Parental health literacy impacts the decision a parent makes relating to their child[31] and is correlated with a number of health indicators including knowledge of health and health services, and the parent and child engaging in health-promoting behaviours[30,32]. Therefore, these findings suggest that the tracking and reporting of SARS-CoV-2 in children and adolescents may be highly skewed towards children whose parents are health literate, and those not accessing testing services remain undetected.

For peer review only - http://bmjopen.bmj.com/site/about/guidelines.xhtml

In addition, it will also be important to consider the mechanism of parental involvement and health literacy in the context of child vaccination. The COVID-19 vaccine has been approved to children aged 12 and above in the UK, with trials currently underway to examine the vaccine response within children aged 5-11 (approved in for example the USA and Israel)[33]. High population-level vaccine uptake is a primary strategy for many governments globally. Thus, the findings in this study suggest future investment should be made to address parental health literacy and its influence on child COVID-19 vaccine uptake. This research has implications for informing public health practice and emerging policy by integrating the views of parents, children and young people to the design of testing services and future vaccination programmes.

Previous research examining transmission in school-aged children found SARS-CoV-2 infections within the household to be the strongest predictor for a subsequent positive SARS-CoV-2 test[34]. Findings in this study that girls are more likely to test positive for SARS-CoV-2 may suggest sex differences between household contact patterns including more repeated, extended contact with household members by girls. This is supported by a study in the United States that finds sex differences between patterns of social interaction in the home, with adolescent females (15 to 19 years) having higher mean number of household member contacts and mean total contact duration[35]. Previous research also demonstrates assortative mixing patterns by age, with the highest frequency of contacts by those aged 10-19 with individuals of the same age group, though this is not stratified by sex[36]. Further research is required in the context of COVID-19 to examine sex differences of adolescent contact patterns in areas of high frequency such as the school setting.

Regarding the association of increasing age with likelihood of testing positive for SARS-CoV-2 in this study, a systematic review and meta-analysis by Viner and colleagues

For peer review only - http://bmjopen.bmj.com/site/about/guidelines.xhtml

determined that whilst children younger than 10 to 14 years have lower susceptibility to SARS-CoV-2 transmission, susceptibility by adolescents may be similar to that of adults[3]. Our findings to do not show an area-level social gradient. Those in WIMD quintile 2 and 4 were less likely (10% significance level) to test positive for SARS-CoV-2 compared to the most deprived quintile. Whilst it is possible that children mixing in the school setting are in contact with children residing in different area-level quintiles, this finding may reflect community prevalence which was not captured in the current study.

STRENGTHS AND LIMITATIONS

Strengths of this study include the reporting of multiple child health behaviours, and the use of individual-level linked routine records of SARS-CoV-2 testing data through the SAIL Databank. This is the first study to investigate association of prior child health-related behaviour measures with subsequent SARS-CoV-2 testing and infection during adolescence in Wales and highlights the importance of targeting and improving parental health literacy to increase uptake of appropriate testing and vaccinations among children. The child-reported health behaviours captured in this study were measured before the COVID-19 pandemic and represent historical health behaviour which may not reflect the child's lifestyle behaviours during COVID-19. It is possible that these behaviours were disrupted due to public health measures in place to reduce transmission. Health behaviours captured through the national-scale HAPPEN survey represent children attending schools that engaged with the HAPPEN Wales primary school network. This may not be representative of the whole population of Wales. The period of study for PCR-testing for and testing positive for SARS-CoV-2 includes a time frame with varying approaches to testing children, public health measures and

For peer review only - http://bmjopen.bmj.com/site/about/guidelines.xhtml

restrictions such as prolonged school closures, schools reopening and other measures to minimise contacts which were not measured in this study. As a result, contacts with infectious people and risk of transmission are likely to have varied. Rates of SARS-CoV-2 transmission also varied during this study, and this study does not differentiate between variants with higher or lower transmissibility, or vaccination status.

CONCLUSION

This study did not find evidence that reporting health-promoting behaviours (e.g. fruit and vegetable consumption, regular physical activity or meeting sleep guidelines) prior to the COVID-19 pandemic reduced the likelihood of having symptoms of COVID-19 (measured as being PCR-tested for SARS-CoV-2) or having a positive test. Instead, this study suggests that actions associated with a child being tested for SARS-CoV-2 and being identified as positive related to health-promoting behaviours may be a proxy of parental health literacy and monitoring behaviours. Further research is required to examine parental health literacy and monitoring behaviours in the context of testing for SARS-CoV-2. Adhering to public health guidance, social distancing, reducing number of contacts and having the vaccine remain the primary means of minimising infection risk. The first vaccine doses are currently being offered to children aged 12-15. Children below the age of 12 are not currently offered first doses. In order to minimise the widespread disruption to children's lives through COVID-19 infection, expanding the vaccination programme to primary school-aged children, community testing, accompanied by following public health guidance reflective of current community transmission rates is important. Based on the proposed theory in this study of the influence parental health literacy on uptake of SARS-CoV-2 testing and the detection of positive cases,

For peer review only - http://bmjopen.bmj.com/site/about/guidelines.xhtml

For peer review only

it is also important to consider this in the context of vaccinating children. This study suggests that we do not know the true rates of COVID-19 in children and adolescents as they are dependent on their parent taking them for testing and this may be determined by the parent's health literacy and understanding.

Acknowledgements

The authors would like to thank all participating primary schools and pupils that took part in this study. This work was supported by the National Centre for Population Health and Wellbeing Research through the HAPPEN network. This study makes use of anonymised data held in the Secure Anonymised Information Linkage (SAIL) Databank. We would like to acknowledge all the data providers who make anonymised data available for research. We would also like to thank Dr Annemarie Docherty and Dr Olivia Swann from The University of Edinburgh for providing informal peer review input to the final draft.

Availability for data and materials

The routine data used in this study are available in the SAIL Databank at Swansea University, Swansea, UK. All proposals to use SAIL data are subject to review by an IGRP. Before any data can be accessed, approval must be given by the IGRP. The IGRP gives careful consideration to each project to ensure proper and appropriate use of SAIL data. When access has been approved, it is gained through a privacy-protecting safe haven and remote access system referred to as the SAIL Gateway. SAIL has established an application process to be followed

For peer review only

by anyone who would like to access data via SAIL https://www.saildatabank.com/application-process. This study has been approved by the IGRP as project 0911.

Funding

The Economic and Social Research Council (ESRC) funded the development of the HAPPEN network (grant number: ES/J500197/1) which this research was conducted through. The National Centre for Population Health and Wellbeing Research (NCPHWR) funded by Health and Care Research Wales provided infrastructural support for this work. This work was supported by the Con-COV team funded by the Medical Research Council (grant number: MR/V028367/1). This work was supported by Health Data Research UK, which receives its funding from HDR UK Ltd (HDR-9006) funded by the UK Medical Research Council, Engineering and Physical Sciences Research Council, Economic and Social Research Council, Department of Health and Social Care (England), Chief Scientist Office of the Scottish Government Health and Social Care Directorates, Health and Social Care Research and Development Division (Welsh Government), Public Health Agency (Northern Ireland), British Heart Foundation (BHF) and the Wellcome Trust. This work was a collaboration with the ADR Wales programme of work. ADR Wales is part of the Economic and Social Research Council (part of UK Research and Innovation) funded ADR UK (grant ES/S007393/1). This work was supported by the Wales COVID-19 Evidence Centre, funded by Health and Care Research Wales and by the COVID-19 Longitudinal Health and Wellbeing National Core Study funded by the Medical Research Council (MC_PC_20030).

For peer review only - http://bmjopen.bmj.com/site/about/guidelines.xhtml

Transparency statement

The lead author affirms that the manuscript is an honest, accurate, and transparent account of the study being reported; that no important aspects of the study have been omitted; and that any discrepancies from the study as originally planned have been explained.

Competing interests

The authors declare that they have no competing interests.

Contributorship statement

EM and SB conceptualised the study design. EM and JK curated the data. EM performed the statistical analysis, undertook the initial interpretation of the data and was responsible for the original draft. EL and SB contributed to the writing of the manuscript. EL, LC, JK, RL and SB provided critical interpretation of the data. The manuscript was critically reviewed and edited by all authors. EM is the guarantor. AD, RL and OS critically reviewed the final manuscript.

References

1 UNICEF. The United Nations Convention on the Rights of the Child. 2004. doi:10.1093/tropej/fmp024

2 OECD. Combatting COVID-19's effect on children - OECD. 2020.https://www.oecd.org/coronavirus/policy-responses/combatting-covid-19-s-

For peer review only - http://bmjopen.bmj.com/site/about/guidelines.xhtml

effect-on-children-2e1f3b2f/ (accessed 1 Oct 2021).

3 Viner RM, Mytton OT, Bonell C, *et al.* Susceptibility to SARS-CoV-2 Infection among Children and Adolescents Compared with Adults: A Systematic Review and Meta-analysis. JAMA Pediatr. 2020. doi:10.1001/jamapediatrics.2020.4573

4 James M, Marchant E, Defeyter MA, *et al.* Impact of school closures on the health and well-being of primary school children in Wales UK: a routine data linkage study using the HAPPEN Survey (2018-2020). *BMJ Open* 2021;**11**. doi:10.1136/BMJOPEN-2021-051574

5 Marchant E, Todd C, James M, *et al.* Primary school staff perspectives of school closures due to COVID-19, experiences of schools reopening and recommendations for the future: A qualitative survey in Wales. *PLoS One* 2021;**16**:e0260396. doi:10.1371/JOURNAL.PONE.0260396

6 Moore SA, Faulkner G, Rhodes RE, *et al.* Impact of the COVID-19 virus outbreak on movement and play behaviours of Canadian children and youth: A national survey. *Int J Behav Nutr Phys Act* 2020;**17**. doi:10.1186/s12966-020-00987-8

7 Craigie AM, Lake AA, Kelly SA, *et al.* Tracking of obesity-related behaviours from childhood to adulthood: A systematic review. *Maturitas* 2011;**70**:266–84. doi:10.1016/j.maturitas.2011.08.005

8 Chowdhury R, Van Daalen KR, Franco OH. Cardiometabolic Health: Key in Reducing Adverse COVID-19 Outcomes. *Glob Heart* 2020;**15**:58. doi:10.5334/gh.879

9 Koch R, Jordan S, Starker A, *et al.* Health behaviour and COVID-19: Initial findings on the pandemic Journal of Health Monitoring 2. *J Heal Monit* 2020;**2020**:1–14. doi:10.25646/7055

10 Childs CE, Calder PC, Miles EA. Diet and Immune Function. *Nutrients* 2019;**11**.

For peer review only - http://bmjopen.bmj.com/site/about/guidelines.xhtml

doi:10.3390/nu11081933

11 Lange KW, Nakamura Y. Lifestyle factors in the prevention of COVID-19. *Glob Heal J* 2020;**4**:146–52. doi:10.1016/J.GLOHJ.2020.11.002

12 Hamer M, Kivimäki M, Gale C, *et al.* Lifestyle risk factors, inflammatory mechanisms, and COVID-19 hospitalization: A community-based cohort study of 387,109 adults in UK. *Brain Behav Immun* 2020;**87**:184–7. doi:10.1016/J.BBI.2020.05.059

13 Lee SW, Lee J, Moon SY, *et al.* Physical activity and the risk of SARS-CoV-2 infection, severe COVID-19 illness and COVID-19 related mortality in South Korea: a nationwide cohort study. *Br J Sports Med* 2021;**0**:18. doi:10.1136/BJSPORTS-2021-104203

14 Zhou B, Yuan Y, Wang S, *et al.* Risk profiles of severe illness in children with COVID-19: a meta-analysis of individual patients. *Pediatr Res 2021 902* 2021;**90**:347–52. doi:10.1038/s41390-021-01429-2

15 Shekerdemian LS, Mahmood NR, Wolfe KK, *et al.* Characteristics and Outcomes of Children With Coronavirus Disease 2019 (COVID-19) Infection Admitted to US and Canadian Pediatric Intensive Care Units. *JAMA Pediatr* 2020;**174**:868–73. doi:10.1001/JAMAPEDIATRICS.2020.1948

16 Castagnoli R, Votto M, Licari A, *et al.* Severe Acute Respiratory Syndrome Coronavirus 2 (SARS-CoV-2) Infection in Children and Adolescents: A Systematic Review. *JAMA Pediatr* 2020;**174**:882–9. doi:10.1001/JAMAPEDIATRICS.2020.1467

17 Todd, C., Christian, D., Tyler, R., Stratton, G., Brophy S. Developing HAPPEN (Health and Attainment of Pupils involved in a Primary Education Network): working in partnership to improve child health and education. *Perspect Public Health* 2016;**136**:115–6.

18 Christian D, Todd C, Davies H, *et al.* Community led active schools programme (CLASP) exploring the implementation of health interventions in primary schools:

For peer review only - http://bmjopen.bmj.com/site/about/guidelines.xhtml

headteachers' perspectives. *BMC Public Health* 2015;**15**:238. doi:10.1186/s12889-015-1557-0

19 Todd C, Christian D, Davies H, *et al.* Headteachers' prior beliefs on child health and their engagement in school based health interventions: a qualitative study. *BMC Res Notes* 2015;**18**:161. doi:10.1186/s13104-015-1091-2

20 Everson B, Mackintosh KA, McNarry MA, *et al.* Can Wearable Cameras be Used to Validate School-Aged Children's Lifestyle Behaviours? *Children* 2019;**6**:20. doi:10.3390/children6020020

21 Ford D V., Jones KH, Verplancke JP, *et al.* The SAIL Databank: Building a national architecture for e-health research and evaluation. *BMC Health Serv Res* 2009;**9**. doi:10.1186/1472-6963-9-157

22 Jones KH, Ford DV, Thompson S, *et al.* A Profile of the SAIL Databank on the UK Secure Research Platform. *Int J Popul Data Sci* 2019;**4**. doi:10.23889/ijpds.v4i2.1134

23 Lyons J, Akbari A, Torabi F, *et al.* Understanding and responding to COVID-19 in Wales: protocol for a privacy-protecting data platform for enhanced epidemiology and evaluation of interventions. *BMJ Open* 2020;**10**:43010. doi:10.1136/bmjopen-2020-043010

24 Benchimol EI, Smeeth L, Guttmann A, *et al.* The REporting of studies Conducted using Observational Routinely-collected health Data (RECORD) Statement. *PLOS Med* 2015;**12**:e1001885. doi:10.1371/JOURNAL.PMED.1001885

25 Welsh Government. Welsh Index of Multiple Deprivation. 2019.https://gov.wales/welsh-index-multiple-deprivation-full-index-update-ranks-2019

26 Mercer TR, Salit M. Testing at scale during the COVID-19 pandemic. *Nat Rev Genet 2021*

For peer review only - http://bmjopen.bmj.com/site/about/guidelines.xhtml

227 2021;**22**:415–26. doi:10.1038/s41576-021-00360-w

27 Pyper E, Harrington D, Manson H. Do parents' support behaviours predict whether or not their children get sufficient sleep? A cross-sectional study. *BMC Public Health* 2017;**17**. doi:10.1186/s12889-017-4334-4

28 Edwardson CL, Gorely T. Parental influences on different types and intensities of physical activity in youth: A systematic review. *Psychol Sport Exerc* 2010;**11**:522–35. doi:10.1016/j.psychsport.2010.05.001

29 Bourdieu P. The Forms of Capital. In: *Handbook of Theory and Research for the Sociology of Education*. New York: : Greenwood 1986. 241–58.

30 Sørensen K, Van den Broucke S, Fullam J, *et al.* Health literacy and public health: A systematic review and integration of definitions and models. Published Online First: 2012. doi:10.1186/1471-2458-12-80

31 Morrison AK, Glick A, Yin HS. Health Literacy: Implications for Child Health. *Pediatr Rev* 2019;**40**:263–77. doi:10.1542/PIR.2018-0027

32 Pawellek M, Kopf FM, Egger N, *et al.* Pathways linking parental health literacy with health behaviours directed at the child: a scoping review. *Health Promot Int* 2021;**2021**:1–29. doi:10.1093/HEAPRO/DAAB154

33 Pfizer. Pfizer and BioNTech Announce Positive Topline Results From Pivotal Trial of COVID-19 Vaccine in Children 5 to 11 Years | Pfizer. 2021.https://www.pfizer.com/news/press-release/press-release-detail/pfizer-and-biontech-announce-positive-topline-results (accessed 10 Dec 2021).

34 Thompson DA, Abbasizanjani H, Fry R, *et al.* Staff-pupil SARS-CoV-2 infection pathways in schools in Wales: A population-level linked data approach. *BMJ Paediatr Open* 2021;**5**:1049. doi:10.1136/bmjpo-2021-001049

For peer review only - http://bmjopen.bmj.com/site/about/guidelines.xhtml

35 Dorélien AM, Ramen A, Swanson I, *et al.* Analyzing the demographic, spatial, and temporal factors influencing social contact patterns in U.S. and implications for infectious disease spread. *BMC Infect Dis* 2021;**21**:1–19. doi:10.1186/S12879-021-06610-W/FIGURES/10

36 Mossong J, Hens N, Jit M, *et al.* Social contacts and mixing patterns relevant to the spread of infectious diseases. *PLoS Med* Published Online First: 2008. doi:10.1371/journal.pmed.0050074

Figure 1: Cohort flow diagram

For peer review only

For peer review only - http://bmjopen.bmj.com/site/about/guidelines.xhtml

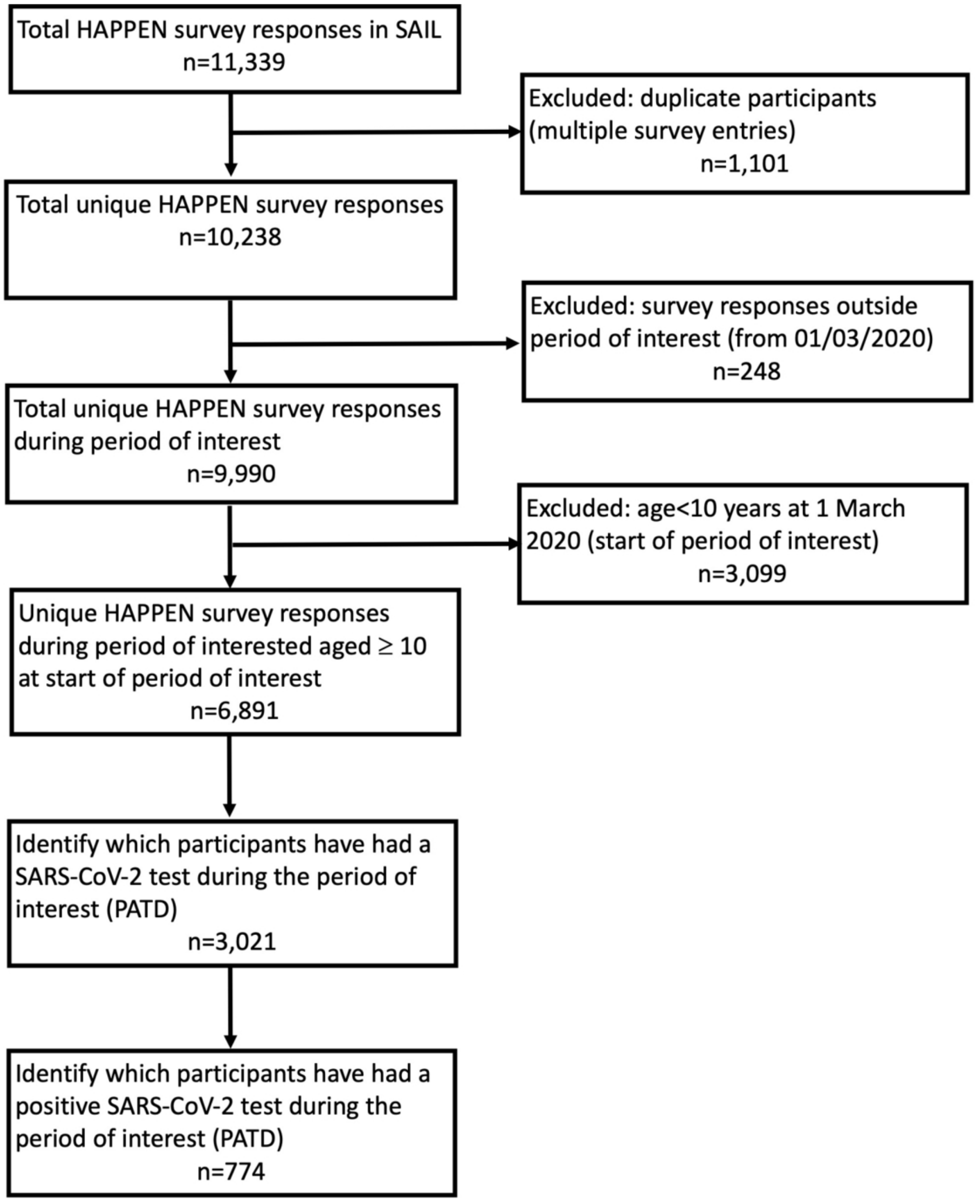

Figure 1: Cohort flow diagram

153x187mm (300 x 300 DPI)

Online supplemental appendix 1: RECORD statement

The RECORD statement – checklist of items, extended from the STROBE statement, that should be reported in observational studies using routinely collected health data.

	Item No.	STROBE items	Location in manuscript where items are reported	RECORD items	Location in manuscript where items are reported
**Title and abstract**					
	1	(a) Indicate the study's design with a commonly used term in the title or the abstract (b) Provide in the abstract an informative and balanced summary of what was done and what was found		RECORD 1.1: The type of data used should be specified in the title or abstract. When possible, the name of the databases used should be included.<br><br>RECORD 1.2: If applicable, the geographic region and timeframe within which the study took place should be reported in the title or abstract.<br><br>RECORD 1.3: If linkage between databases was conducted for the study, this should be clearly stated in the title or abstract.	1.1: Title and abstract (page 1-2)<br><br>1.2: Title and abstract (page 1-2)
**Introduction**					
Background rationale	2	Explain the scientific background and rationale for the investigation being reported			Background (page 5-7)
Objectives	3	State specific objectives, including any prespecified hypotheses			Background (page 5-7)
**Methods**					
Study Design	4	Present key elements of study design early in the paper			Methods - Study design (page 7)
Setting	5	Describe the setting, locations, and relevant dates, including			Methods - Study design (page 7)

For peer review only

Online supplemental appendix 1: RECORD statement

		periods of recruitment, exposure, follow-up, and data collection			
Participants	6	*(a) Cohort study* - Give the eligibility criteria, and the sources and methods of selection of participants. Describe methods of follow-up<br>*Case-control study* - Give the eligibility criteria, and the sources and methods of case ascertainment and control selection. Give the rationale for the choice of cases and controls<br>*Cross-sectional study* - Give the eligibility criteria, and the sources and methods of selection of participants<br><br>*(b) Cohort study* - For matched studies, give matching criteria and number of exposed and unexposed<br>*Case-control study* - For matched studies, give matching criteria and the number of controls per case		RECORD 6.1: The methods of study population selection (such as codes or algorithms used to identify subjects) should be listed in detail. If this is not possible, an explanation should be provided.<br><br>RECORD 6.2: Any validation studies of the codes or algorithms used to select the population should be referenced. If validation was conducted for this study and not published elsewhere, detailed methods and results should be provided.<br><br>RECORD 6.3: If the study involved linkage of databases, consider use of a flow diagram or other graphical display to demonstrate the data linkage process, including the number of individuals with linked data at each stage.	6.1: Figure 1: Cohort Flow Diagram (page 11)<br><br>6.3: Figure 1: Cohort Flow Diagram (page 11)
Variables	7	Clearly define all outcomes, exposures, predictors, potential confounders, and effect modifiers. Give diagnostic criteria, if applicable.		RECORD 7.1: A complete list of codes and algorithms used to classify exposures, outcomes, confounders, and effect modifiers should be provided. If these cannot be reported, an explanation should be provided.	7.1: Supplemental appendix 3: HAPPEN survey variable codebook
Data sources/ measurement	8	For each variable of interest, give sources of data and details of methods of assessment (measurement).			Methods - The HAPPEN survey and linked SAIL data (Page 8-9)

For peer review only

Online supplemental appendix 1: RECORD statement

		Describe comparability of assessment methods if there is more than one group			
Bias	9	Describe any efforts to address potential sources of bias			Methods - Quantitative analysis (page 9)
Study size	10	Explain how the study size was arrived at			Figure 1: Cohort flow diagram (page 11)
Quantitative variables	11	Explain how quantitative variables were handled in the analyses. If applicable, describe which groupings were chosen, and why			Methods - Quantitative analysis (page 10)
Statistical methods	12	(a) Describe all statistical methods, including those used to control for confounding<br>(b) Describe any methods used to examine subgroups and interactions<br>(c) Explain how missing data were addressed<br>(d) *Cohort study* - If applicable, explain how loss to follow-up was addressed<br>*Case-control study* - If applicable, explain how matching of cases and controls was addressed<br>*Cross-sectional study* - If applicable, describe analytical methods taking account of sampling strategy<br>(e) Describe any sensitivity analyses			Methods - Quantitative analysis (page 10)

For peer review only

Online supplemental appendix 1: RECORD statement

Data access and cleaning methods		..		RECORD 12.1: Authors should describe the extent to which the investigators had access to the database population used to create the study population.<br><br>RECORD 12.2: Authors should provide information on the data cleaning methods used in the study.	12.1: Methods - The HAPPEN survey and linked SAIL data (page 8-9)<br><br>12.2: Figure 1 – Cohort flow diagram (page 11)
Linkage		..		RECORD 12.3: State whether the study included person-level, institutional-level, or other data linkage across two or more databases. The methods of linkage and methods of linkage quality evaluation should be provided.	12.3: Methods - Study design (page 7)<br><br>The HAPPEN survey and linked SAIL data (page 8-9)
**Results**					
Participants	13	(a) Report the numbers of individuals at each stage of the study (*e.g.*, numbers potentially eligible, examined for eligibility, confirmed eligible, included in the study, completing follow-up, and analysed)<br>(b) Give reasons for non-participation at each stage.<br>(c) Consider use of a flow diagram		RECORD 13.1: Describe in detail the selection of the persons included in the study (*i.e.*, study population selection) including filtering based on data quality, data availability and linkage. The selection of included persons can be described in the text and/or by means of the study flow diagram.	13.1: Methods - Quantitative analysis (page 10-11)<br><br>Figure 1: Cohort flow diagram (page 11)
Descriptive data	14	(a) Give characteristics of study participants (*e.g.*, demographic, clinical, social) and information on exposures and potential confounders			Results - Table 1 and table 2 Descriptive statistics (page 12-15)

For peer review only

Online supplemental appendix 1: RECORD statement

		(b) Indicate the number of participants with missing data for each variable of interest<br>(c) *Cohort study* - summarise follow-up time (*e.g.*, average and total amount)			
Outcome data	15	*Cohort study* - Report numbers of outcome events or summary measures over time<br>*Case-control study* - Report numbers in each exposure category, or summary measures of exposure<br>*Cross-sectional study* - Report numbers of outcome events or summary measures			Results - Table 1 and table 2, Descriptive statistics (page 12-15)
Main results	16	(a) Give unadjusted estimates and, if applicable, confounder-adjusted estimates and their precision (e.g., 95% confidence interval). Make clear which confounders were adjusted for and why they were included<br>(b) Report category boundaries when continuous variables were categorized<br>(c) If relevant, consider translating estimates of relative risk into absolute risk for a meaningful time period			Results – Table 3 and table 4 (page 16-17)
Other analyses	17	Report other analyses done—e.g., analyses of subgroups and interactions, and sensitivity analyses			Results (page 12)
**Discussion**					

For peer review only

Online supplemental appendix 1: RECORD statement

Key results	18	Summarise key results with reference to study objectives			Results (page 12-18)
Limitations	19	Discuss limitations of the study, taking into account sources of potential bias or imprecision. Discuss both direction and magnitude of any potential bias		RECORD 19.1: Discuss the implications of using data that were not created or collected to answer the specific research question(s). Include discussion of misclassification bias, unmeasured confounding, missing data, and changing eligibility over time, as they pertain to the study being reported.	Strengths and limitations (page 21-22)
Interpretation	20	Give a cautious overall interpretation of results considering objectives, limitations, multiplicity of analyses, results from similar studies, and other relevant evidence			Discussion (page 18-21), Conclusion (page 22-23)
Generalisability	21	Discuss the generalisability (external validity) of the study results			Discussion (page 18-21), Conclusion (page 22-23), Strengths and limitations (page 21-22)
**Other Information**					
Funding	22	Give the source of funding and the role of the funders for the present study and, if applicable, for the original study on which the present article is based			Funding (page 24)
Accessibility of protocol, raw data, and programming code		..		RECORD 22.1: Authors should provide information on how to access any supplemental information such as the study protocol, raw data, or programming code.	Availability for data and materials (page 23)

For peer review only

Online supplemental appendix 1: RECORD statement

*Reference: Benchimol EI, Smeeth L, Guttmann A, Harron K, Moher D, Petersen I, Sørensen HT, von Elm E, Langan SM, the RECORD Working Committee. The REporting of studies Conducted using Observational Routinely-collected health Data (RECORD) Statement. *PLoS Medicine* 2015; in press.

*Checklist is protected under Creative Commons Attribution (CC BY) license.

For peer review only

THE HAPPEN SURVEY

* Required

Consent Form

Before you start please click this link to read the information sheet -> https://happen-wales.co.uk/wp-content/uploads/2019/02/Child-Consent-2019.pdf

1. I have read the child information sheet -> https://happen-wales.co.uk/wp-content/uploads/2019/02/Child-Consent-2019.pdf (click the link if you haven't read it) and understand that if I take part I can change my mind at any time, and this will not be a problem at all. *

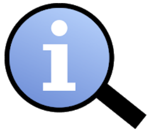

Mark only one oval.

- ◯ Yes
- ◯ No

2. I am happy for you to use my questionnaire for research. Only the researchers in the team will know my name and will not tell anyone else my answers *

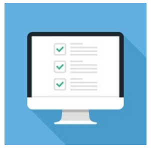

Mark only one oval.

- ◯ Yes
- ◯ No do not use my questionnaire

3. I am happy for you to look at my school and health records to see how my school is doing (as a group). This is anonymous which means I cannot be identified *

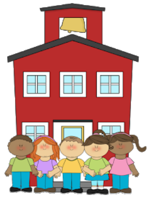

Mark only one oval.

- ◯ Yes
- ◯ No

If you do not wish to take part in the questionnaire please do not continue.

Please click next to start the questionnaire!

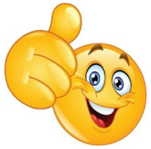

About You

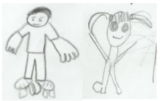

4. First Name *

5. Last Name *

6. Home Post Code *

7. What school do you go to? *

8. What year are you in? *

Mark only one oval.

- ◯ Year 4
- ◯ Year 5
- ◯ Year 6
- ◯ Year 7

9. Gender *

Mark only one oval.

- ◯ Boy
- ◯ Girl
- ◯ Prefer not to say

Date of Birth

10. Year *

Mark only one oval.

- ◯ 2007
- ◯ 2008
- ◯ 2009
- ◯ 2010
- ◯ 2011
- ◯ 2012

For peer review only - http://bmjopen.bmj.com/site/about/guidelines.xhtml

11. Month *

Mark only one oval.

- January
- February
- March
- April
- May
- June
- July
- August
- September
- October
- November
- December

12. Day *

Mark only one oval.

- 1
- 2
- 3
- 4
- 5
- 6
- 7
- 8
- 9
- 10
- 11
- 12
- 13
- 14
- 15
- 16
- 17
- 18
- 19
- 20
- 21
- 22
- 23
- 24
- 25
- 26
- 27
- 28
- 29
- 30
- 31

YESTERDAY

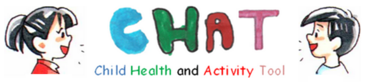

Firstly, think carefully about what you did YESTERDAY and then answer the following questions....

13. 1. What did you eat for breakfast YESTERDAY? *

Check all that apply.

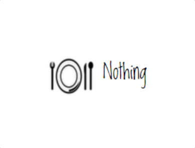

- Nothing

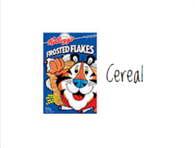

- Sugary cereal e.g. cocopops, frosties, sugar puffs, chocolate cereals

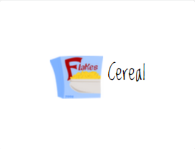

- Healthy cereal e.g. porridge, weetabix, readybrek, muesli, branflakes, cornflakes

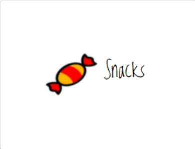

- Snacks

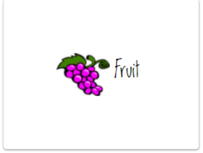

- Fruit

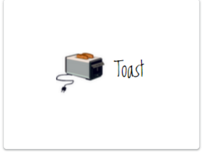

- Toast

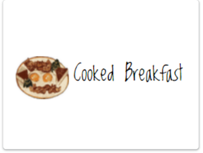

- Cooked breakfast

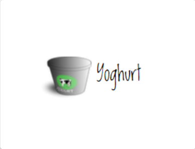

- Yoghurt

Other: ______

For peer review only

14. 2. How did you get to school YESTERDAY morning? *

Mark only one oval.

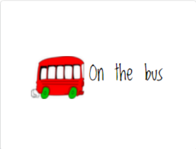

On the bus

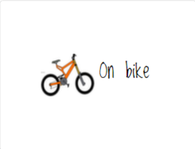

On bike

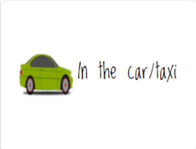

In the car/taxi

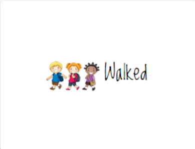

Walked

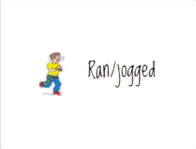

Ran/jogged

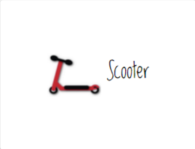

Scooter

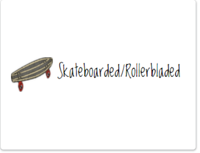

Skateboarded/Rollerbladed

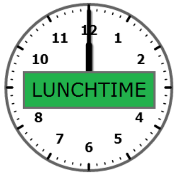

15. 3. What did you have to eat for lunch YESTERDAY? *

Mark only one oval.

- School dinner
- Packed lunch
- Nothing

16. 4. What did you do for MOST of your break-times YESTERDAY? (This includes lunchtime) *

Mark only one oval.

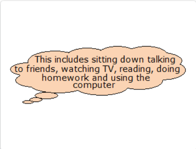

Sat around inside or outside

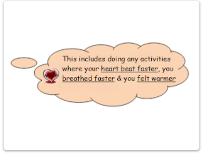

Ran around

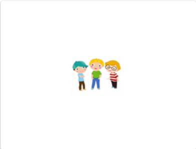

Stood around

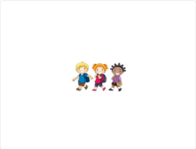

Walked around

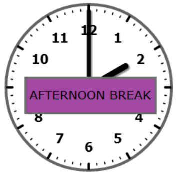

17. 5. Do you have an afternoon break at school? *

Mark only one oval.

- YES
- NO

For peer review only

18. 6. How did you get home YESTERDAY? *

Mark only one oval.

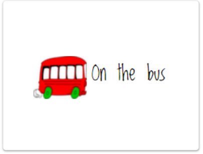

( ) On the bus

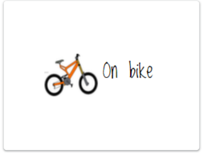

( ) On bike

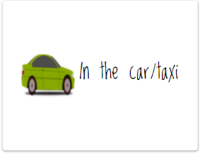

( ) In the car/taxi

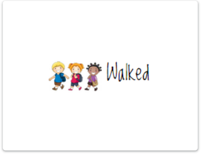

( ) Walked

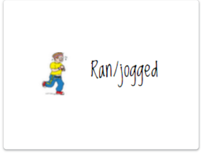

( ) Ran/jogged

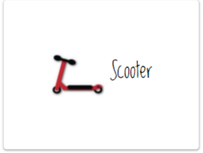

( ) Scooter

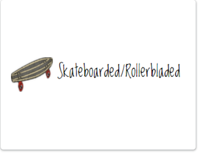

( ) Skateboarded/Rollerbladed

AFTER SCHOOL

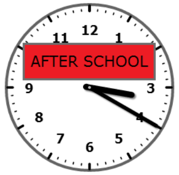

19. 7. How many portions of fruit and vegetables did you eat YESTERDAY? *

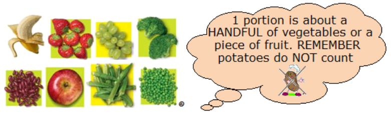

Mark only one oval.

( ) 0
( ) 1
( ) 2
( ) 3
( ) 4
( ) 5
( ) 6
( ) 7
( ) 8

20. 8. How many times did you brush your teeth YESTERDAY? *

Mark only one oval.

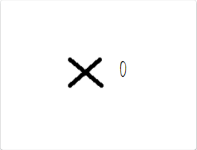

( ) 0

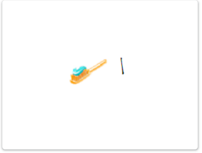

( ) 1

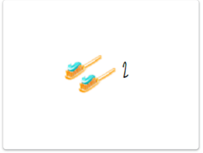

( ) 2

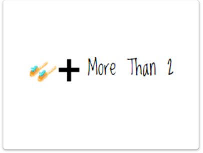

( ) 3

21. 9. What time did you fall asleep YESTERDAY (to the nearest half hour)? *

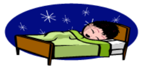

Mark only one oval.

( ) 7:00pm
( ) 7:30pm
( ) 8:00pm
( ) 8:30pm
( ) 9:00pm
( ) 9:30pm
( ) 10:00pm
( ) 10:30pm
( ) 11:00pm
( ) 11:30pm
( ) 12:00am
( ) 12:30am
( ) 1:00am
( ) 1:30am
( ) 2:00am
( ) 3:00am
( ) 3:30am
( ) 4:00am

For peer review only - http://bmjopen.bmj.com/site/about/guidelines.xhtml

22. 10. What time did you wake up TODAY (to the nearest half hour)? *

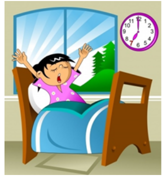

Mark only one oval.

- 5:00am
- 5:30am
- 6:00am
- 6:30am
- 7:00am
- 7:30am
- 8:00am
- 8:30am
- 9:00am

THE LAST WEEK

NOW think about what you did in the last 7 days...

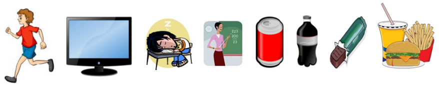

23. 11a. In the last 7 days, how many days did you do sports or exercise for at least 1 hour in total (This includes doing any activities or playing sports where your heart beat faster, you breathed faster and you felt warmer? *

Mark only one oval.

- 0 days
- 1-2 days
- 3-4 days
- 5-6 days
- 7 days

24. 11b. In the last 7 days, how many days did you watch TV/play online games/use the internet etc. for 2 or more hours a day (in total)? *

Mark only one oval.

- 0 days
- 1-2 days
- 3-4 days
- 5-6 days
- 7 days

25. 11c. In the last 7 days, how many days did you feel tired? *

Mark only one oval.

- 0 days
- 1-2 days
- 3-4 days
- 5-6 days
- 7 days

26. 11d. In the last 7 days, how many days did you feel like you could concentrate/pay attention well in class? *

Mark only one oval.

- 0 days
- 1-2 days
- 3-4 days
- 5-6 days
- 7 days

27. 11e. In the last 7 days, how many days did you drink at least one fizzy drink (e.g. coke, fanta, sprite) *

Mark only one oval.

- 0 days
- 1-2 days
- 3-4 days
- 5-6 days
- 7 days

28. 11f. In the last 7 days, how many days did you eat at least one sugary snack (e.g. chocolate bar, sweets) *

Mark only one oval.

- 0 days
- 1-2 days
- 3-4 days
- 5-6 days
- 7 days

29. 11g. In the last 7 days, how many days did you eat take away foods (e.g. McDonalds, KFC, chinese) *

Mark only one oval.

- 0 days
- 1-2 days
- 3-4 days
- 5-6 days
- 7 days

Sport and Activity

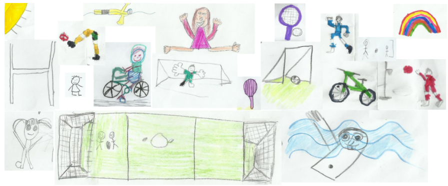

30. 12. These questions are going to ask you how you feel about physical activity (This includes any activity where your heart beats faster, you breathe faster and you feel warmer) *

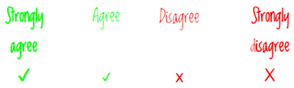

Mark only one oval per row.

	Strongly agree	Agree	Disagree	Strongly disagree
I want to take part in physical activity	○	○	○	○
I feel confident to take part in lots of different physical activities	○	○	○	○
I am good at lots of different physical activities	○	○	○	○
I understand why taking part in physical activity is good for me	○	○	○	○

31. 13a. How many times do you take part in a sports club OUTSIDE OF SCHOOL each week?

Mark only one oval.

0	1	2	3	4	5	6	7	8	9	10
◯	◯	◯	◯	◯	◯	◯	◯	◯	◯	◯

32. 13b. If you take part in a sports club OUTSIDE of school, what is the name of the sports club? (For example Swansea Rugby Club Under 11's)

____

33. 14. Are you a member of cubs, brownies, scouts or guides? *

Mark only one oval.

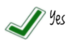

◯ Yes

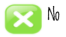

◯ No

34. 15. Which of these sports or physical activities would you MOST like to try? (That you haven't tried before) *

Mark only one oval.

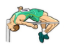

◯ Athletics

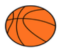

◯ Basketball

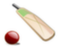

◯ Cricket

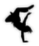

◯ Dance

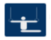

◯ Gymnastics

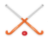

◯ Hockey

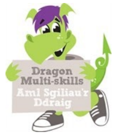

◯ Multi Skills

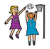

◯ Netball

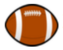

◯ Rugby

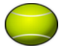

◯ Tennis

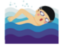

◯ Swimming

◯ I do not want to try anything-I don't like sport or activity

◯ Other: _______________

peer revi

For peer review only - http://bmjopen.bmj.com/site/about/guidelines.xhtml

35. 16. Can you ride a bike WITHOUT STABILISERS? *

Mark only one oval.

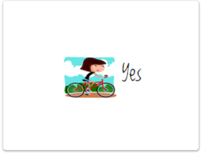

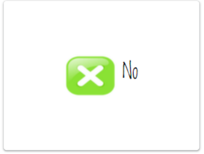

- ( ) Yes
- ( ) No

36. 17. Can you swim 25 metres WITHOUT A FLOAT OR ARMBANDS? (This is 1 length of a standard swimming pool) *

Mark only one oval.

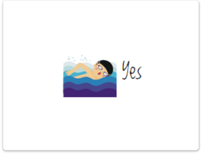

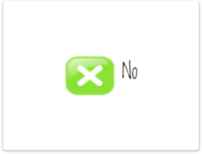

- ( ) Yes
- ( ) No

You and your feelings

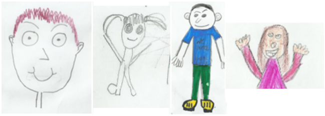

This part of the survey is going to ask you how you feel. There are no right or wrong answers. You should just pick the answer which is best for you.

37. 18. Tell us if you agree or disagree with the following: *

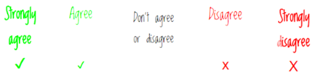

Mark only one oval per row.

	Strongly agree	Agree	Don't agree or disagree	Disagree	Strongly disagree
I am doing well at school	( )	( )	( )	( )	( )
I have lots of choice over things that are important to me	( )	( )	( )	( )	( )
There are lots of things I'm good at	( )	( )	( )	( )	( )

19. On a scale of 0 to 10 (0 being very unhappy and 10 being very happy, how do you feel about:

*Based on the Good Childhood Index by the Children's Society

38. Your Health *

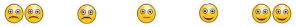

Mark only one oval.

	0	1	2	3	4	5	6	7	8	9	10	
Very unhappy	( )	( )	( )	( )	( )	( )	( )	( )	( )	( )	( )	Very happy

39. Your School *

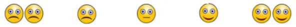

Mark only one oval.

	0	1	2	3	4	5	6	7	8	9	10	
Very unhappy	( )	( )	( )	( )	( )	( )	( )	( )	( )	( )	( )	Very happy

40. Your Family *

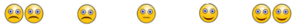

Mark only one oval.

	0	1	2	3	4	5	6	7	8	9	10	
Very unhappy	( )	( )	( )	( )	( )	( )	( )	( )	( )	( )	( )	Very happy

41. Your Friends *

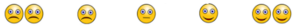

Mark only one oval.

	0	1	2	3	4	5	6	7	8	9	10	
Very unhappy	( )	( )	( )	( )	( )	( )	( )	( )	( )	( )	( )	Very happy

42. Your Appearance (how you look) *

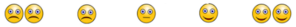

Mark only one oval.

	0	1	2	3	4	5	6	7	8	9	10	
Very unhappy	( )	( )	( )	( )	( )	( )	( )	( )	( )	( )	( )	Very happy

43. Your Life *

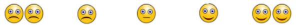

Mark only one oval.

	0	1	2	3	4	5	6	7	8	9	10	
Very unhappy	( )	( )	( )	( )	( )	( )	( )	( )	( )	( )	( )	Very happy

You and your Feelings

Based on the Me and My Feelings Questionnaire (Deighton, Tymms, Vostanis, Belsky, Fonagy, Brown, Martin, Patalay, & Wolpert, 2012)

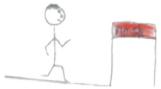

44. 20. Remember, there are no right or wrong answers, just pick which is right for you. *

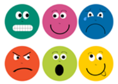

Mark only one oval per row.

	Never	Sometimes	Always
I feel lonely	◯	◯	◯
I cry a lot	◯	◯	◯
I am unhappy	◯	◯	◯
I feel nobody likes me	◯	◯	◯
I worry a lot	◯	◯	◯
I have problems sleeping	◯	◯	◯
I wake up in the night	◯	◯	◯
I am shy	◯	◯	◯
I feel scared	◯	◯	◯
I worry when I am at school	◯	◯	◯
I get very angry	◯	◯	◯
I lose my temper	◯	◯	◯
I hit out when I am angry	◯	◯	◯
I do things to hurt people	◯	◯	◯
I am calm	◯	◯	◯
I break things on purpose	◯	◯	◯

Your Local Area

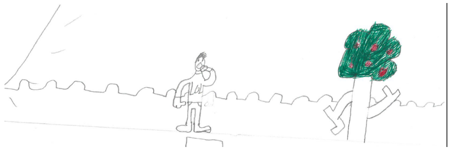

45. 21. On a scale of 0 to 10 (0 being not very safe and 10 being very safe), how safe do you feel playing in your area? *

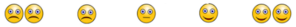

Mark only one oval.

	0	1	2	3	4	5	6	7	8	9	10	
Not very safe	◯	◯	◯	◯	◯	◯	◯	◯	◯	◯	◯	Very safe

46. 22a. From your house, can you walk to school?

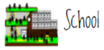

Mark only one oval.

◯ Yes
◯ No

47. 22b. From your house, can you easily walk to a park?

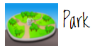

Mark only one oval.

◯ Yes
◯ No

48. 22c. From your house, can you easily walk to a leisure centre/sports centre?

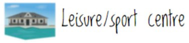

Mark only one oval.

◯ Yes
◯ No

49. 23. Are you happy with the area that you live in?

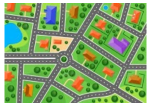

Mark only one oval.

◯ Yes
◯ No

24. If you could change something to make you and your friends healthier and happier, what would you change...

50. IN SCHOOL? *

51. OUT OF SCHOOL? *

Well done, you've completed the questionnaire.
Thank you!

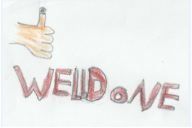

Don't forget to press submit below!

This content is neither created nor endorsed by Google.

Google Forms

For peer review only

Online supplemental appendix 3: HAPPEN survey variable codebook

Exposures	HAPPEN Survey item	Responses	Analyses coding
**Ate breakfast**	*13. What did you eat for breakfast yesterday?*	*Categorical:*<br>*Nothing*<br>*Cereal*<br>*Snacks*<br>*Fruit*<br>*Toast*<br>*Cooked breakfast*<br>*Yoghurt*	Binary:<br>*0 = Nothing*<br>*1 = Cereal; Snacks;*<br>*Fruit; Toast; Cooked breakfast; Yoghurt*
**Active travel to school**	*14. How did you get to school yesterday morning?*	*Categorical:*<br>*On the bus*<br>*In the car/taxi*<br>*Walked*<br>*On bike*<br>*Ran/jogged*<br>*Scooter*<br>*Skateboarded/rollerbladed*	Binary:<br>*0 = On the bus; In the car/taxi*<br>*1 = Walked; On bike; Ran/jogged;*<br>*Scooter; Skateboarded/rollerbladed*
**Active travel from school**	*18. How did you get home yesterday?*	Categorical:<br>*On the bus*<br>*In the car/taxi*<br>*Walked*<br>*On bike*<br>*Ran/jogged*<br>*Scooter*<br>*Skateboarded/rollerbladed*	Binary:<br>*0 = On the bus; In the car/taxi*<br>*1 = Walked; On bike; Ran/jogged;*<br>*Scooter; Skateboarded/rollerbladed*
**Toothbrush 2+ per day**	*20. How many times did you brush your teeth yesterday?*	Continuous:<br>*0 – 3*	Binary:<br>*0 = 0, 1*<br>*1= 2, 3*

For peer review only

Online supplemental appendix 3: HAPPEN survey variable codebook

**5+ fruit and veg**	*19. How many portions of fruit and vegetables did you eat yesterday?*	Continuous:<br>*0 – 8*	Binary:<br>*0 = 0 – 4*<br>*1 = ≥5*
**Sleep 9+ hours**	*21. What time did you fall asleep last night*	Categorical:<br>*(30 min intervals)*<br>*7:00pm – 4:00am*	Binary:<br>*Sleep hours calculated from 21 and 22*<br><br>*0 = < 9 hours*<br>*1 = ≥9 hours*
	22. What time did you wake up this morning?	Categorical:<br>*(30 min intervals)*<br>*5:00am – 9:00am*	
**Physically active 60+ mins every day previous 7 days**	*23. In the last 7 days, how many days did you do sports or exercise for at least 1 hour in total (This includes doing any activities or playing sports where your heart beat faster, you breathed faster and you felt warmer*	Categorical:<br>*0 days*<br>*1 – 2 days*<br>*3 – 4 days*<br>*5 – 6 days*<br>*7 days*	Binary:<br>*0 = 0 days; 1 – 2 days; 3 – 4 days, 5 – 6 days*<br>*1 = 7 days*
**Sedentary/screen time 2 hours every day previous 7 days**	*24. In the last 7 days, how many days did you watch TV/play online games/use the internet etc. for 2 or more hours a day (in total)?*	Categorical:<br>*0 days*<br>*1 – 2 days*<br>*3 – 4 days*<br>*5 – 6 days*<br>*7 days*	Binary:<br>*0 = 0 days; 1 – 2 days; 3 – 4 days, 5 – 6 days*<br>*1 = 7 days*

For peer review only

Online supplemental appendix 3: HAPPEN survey variable codebook

**Tired 7 days**	*25. In the last 7 days, how many days did you feel tired?*	Categorical:<br>*0 days*<br>*1 – 2 days*<br>*3 – 4 days*<br>*5 – 6 days*<br>*7 days*	Binary:<br>*0 = 0 days; 1 – 2 days; 3 – 4 days, 5 – 6 days*<br>*1 = 7 days*
**Sugary snack 7 days**	*28. In the last 7 days, how many days did you eat at least one sugary snack (e.g. chocolate bar, sweets)*	Categorical:<br>*0 days*<br>*1 – 2 days*<br>*3 – 4 days*<br>*5 – 6 days*<br>*7 days*	Binary:<br>*0 = 0 days; 1 – 2 days; 3 – 4 days, 5 – 6 days*<br>*1 = 7 days*
Participate in at least 3 out of school clubs	*31. How many times do you take part in a sports club OUTSIDE OF SCHOOL each week?*	Continuous:<br>*0 - 10*	Binary:<br>*0 = < 3*<br>*1 = ≥ 3*
Can ride a bike	*35. Can you ride a bike without stabilisers?*	Binary:<br>*No*<br>*Yes*	Binary:<br>*0 = No*<br>*1 = Yes*
Can swim 25m	*36. Can you swim 25 metres without a float or armbands (This is 1 length of a standard swimming pool)*	Binary<br>*No*<br>*Yes*	Binary:<br>*0 = No*<br>*1 = Yes*
Age on 01/03/2020	*Decimal age on 1 March 2020*	Continuous	Continuous
Sex	*Sex*	Categorical<br>*Girl*<br>*Boy*	Binary:<br>*0 = Girl*<br>*1 = Boy*
WIMD	*Welsh Index of Multiple Deprivation 2019*		Coding framework from WIMD 2019[25]

Online supplemental appendix 4: Multivariable logistic regression model of health behaviour markers and probability of being PCR-tested for SARS-CoV-2 accounting for baseline age, sex and deprivation, and clustered by school

**PCR-tested for SARS-CoV-2 (0; no, 1; yes)** ***Number of obs=5,555*** ***R^2=0.006***	**OR**	***P***	**95% CI**
Ate breakfast	0.93	0.504	0.76 to 1.14
Active travel to school	0.94	0.479	0.78 to 1.12
Active travel from school	0.99	0.938	0.85 to 1.16
Consumed ≥ five fruit & veg	0.90*	0.088	0.78 to 1.02
Toothbrush ≥ two times	1.00	0.962	0.89 to 1.13
Sleep ≥ nine hours	1.14**	0.044	1.00 to 1.30
Physically active ≥ 60 mins every day previous seven days	1.02	0.831	0.88 to 1.18
Sedentary/screen time ≥ 2 hours every day previous seven days	0.96	0.494	0.86 to 1.08
Tired every day previous seven days	1.08	0.285	0.93 to 1.26
Sugary snack every day previous seven days	1.03	0.713	0.87 to 1.22
Participate in ≥ three out of school clubs per week	1.17**	0.020	1.03 to 1.34
Can ride a bike	1.15*	0.095	0.98 to 1.36
Can swim 25m	1.30**	0.001	1.11 to 1.52
Age on 1 March 2020 (start of period of interest)	1.02	0.300	0.98 to 1.05
Sex (girl)	0.98	0.692	0.87 to 1.10
WIMD quintile 2	1.09	0.328	0.91 to 1.31
3	0.96	0.617	0.80 to 1.14
4	1.09	0.448	0.88 to 1.35
5 (least deprived)	1.05	0.576	0.89 to 1.23

OR: Odds Ratio; 95% CI: 95% confidence intervals; p<0.05**, p<0.1*. See online supplemental appendix 3 for variable codebook.

For peer review only

For peer review only

Online supplemental appendix 5: Multivariable logistic regression model of health behaviour markers and probability of PCR-testing positive for SARS-CoV-2 accounting for baseline age, sex and deprivation, and clustered by school.

**PCR-test positive for SARS-CoV-2 (0; not tested, negative test, 1; test positive)** ***Number of obs=5,555*** ***R^2=0.02***	**OR**	**P**	**95% CI**
Ate breakfast	1.06	0.761	0.75 to 1.49
Active travel to school	0.92	0.494	0.73 to 1.17
Active travel from school	0.97	0.798	0.77 to 1.22
Consumed ≥five fruit & veg	1.05	0.689	0.84 to 1.31
Toothbrush ≥two times	1.03	0.802	0.83 to 1.28
Sleep ≥nine hours	1.01	0.927	0.77 to 1.32
Physically active ≥60 mins every day previous seven days	0.98	0.870	0.8 to 1.21
Sedentary/screen time ≥2 hours every day previous seven days	1.08	0.427	0.90 to 1.30
Tired every day previous seven days	0.78**	0.035	0.62 to 0.98
Sugary snack every day previous seven days	1.03	0.827	0.81 to 1.31
Participate in ≥three out of school clubs per week	1.23*	0.080	0.98 to 1.54
Can ride a bike	1.30	0.132	0.92 to 1.84
Can swim 25m	1.21	0.165	0.92 to 1.59
Age on 1 March 2020 (start of period of interest)	1.16**	0.000	1.10 to 1.22
Sex (girl)	1.23**	0.012	1.04 to 1.45
WIMD quintile 2	0.77*	0.053	0.60 to 1.00
3	0.99	0.945	0.78 to 1.27
4	0.72**	0.032	0.53 to 0.97
5 (least deprived)	0.80	0.075	0.62 to 1.02

OR: Odds Ratio; 95% CI: 95% confidence intervals; p<0.05**, p<0.1*. See online supplemental appendix 3 for variable codebook.

For peer review only

BMJ Open

Pre-COVID-19 pandemic health-related behaviours in children (2018-2020) and association with being tested for SARS-CoV-2 and testing positive for SARS-CoV-2 (2020-2021): a retrospective cohort study using survey data linked with routine health data in Wales, UK

Journal:	*BMJ Open*
Manuscript ID	bmjopen-2022-061344.R1
Article Type:	Original research
Date Submitted by the Author:	28-Apr-2022
Complete List of Authors:	Marchant, Emily ; Swansea University, Population Data Science, Medical School; Swansea University, National Centre for Population Health and Wellbeing Research<br>Lowthian, Emily; Swansea University, Population Data Science, Medical School; Swansea University, Department of Education and Childhood Studies<br>Crick, Tom; Swansea University, Department of Education & Childhood Studies<br>Griffiths, Lucy ; Swansea University, Population Data Science, Medical School<br>Fry, Richard; Swansea University, Medical School; Swansea University, National Centre for Population Health and Wellbeing Research<br>Dadaczynski, Kevin; Fulda University of Applied Sciences, Department of Nursing and Health Science; Leuphana University Luneburg Center of Applied Health Sciences<br>Okan, Orkan; Technical University of Munich<br>James, M; Swansea University, Population Data Science, Medical School; Swansea University, National Centre for Population Health and Wellbeing Research<br>Cowley, Laura; Swansea University, Population Data Science, Medical School<br>Torabi, Fatemeh; Swansea University Medical School, Population Data Science<br>Kennedy, Jonathan; Swansea University, Population Data Science, Medical School; Swansea University, National Centre for Population Health and Wellbeing Research<br>Akbari, Ashley; Swansea University, Population Data Science, Medical School<br>Lyons, Ronan; Swansea University, Population Data Science, Medical School<br>Brophy, Sinead; Swansea University, Population Data Science, Medical School; Swansea University, National Centre for Population Health and Wellbeing Research
**Primary Subject Heading**:	Public health

Secondary Subject Heading:	Epidemiology, Infectious diseases, Paediatrics
Keywords:	Epidemiology < INFECTIOUS DISEASES, PUBLIC HEALTH, Public health < INFECTIOUS DISEASES, COVID-19, Community child health < PAEDIATRICS

SCHOLARONE™ Manuscripts

I, the Submitting Author has the right to grant and does grant on behalf of all authors of the Work (as defined in the below author licence), an exclusive licence and/or a non-exclusive licence for contributions from authors who are: i) UK Crown employees; ii) where BMJ has agreed a CC-BY licence shall apply, and/or iii) in accordance with the terms applicable for US Federal Government officers or employees acting as part of their official duties; on a worldwide, perpetual, irrevocable, royalty-free basis to BMJ Publishing Group Ltd ("BMJ") its licensees and where the relevant Journal is co-owned by BMJ to the co-owners of the Journal, to publish the Work in this journal and any other BMJ products and to exploit all rights, as set out in our licence.

The Submitting Author accepts and understands that any supply made under these terms is made by BMJ to the Submitting Author unless you are acting as an employee on behalf of your employer or a postgraduate student of an affiliated institution which is paying any applicable article publishing charge ("APC") for Open Access articles. Where the Submitting Author wishes to make the Work available on an Open Access basis (and intends to pay the relevant APC), the terms of reuse of such Open Access shall be governed by a Creative Commons licence – details of these licences and which Creative Commons licence will apply to this Work are set out in our licence referred to above.

Other than as permitted in any relevant BMJ Author's Self Archiving Policies, I confirm this Work has not been accepted for publication elsewhere, is not being considered for publication elsewhere and does not duplicate material already published. I confirm all authors consent to publication of this Work and authorise the granting of this licence.

For peer review only

Pre-COVID-19 pandemic health-related behaviours in children (2018-2020) and association with being tested for SARS-CoV-2 and testing positive for SARS-CoV-2 (2020-2021): a retrospective cohort study using survey data linked with routine health data in Wales, UK

Emily Marchant[1,2*], Emily Lowthian[1,3], Tom Crick[3], Lucy Griffiths[1], Richard Fry[1,2], Kevin Dadaczynski[4,5], Orkan Okan[6], Michaela James[1,2], Laura Cowley[2,7] Fatemeh Torabi[1], Jonathan Kennedy[1,2], Ashley Akbari[1], Ronan Lyons[1], Sinead Brophy[1,2]

[1] Population Data Science, Medical School, Swansea University, Swansea, SA2 8PP, United Kingdom

[2] National Centre for Population Health and Wellbeing Research, Swansea University, Swansea, SA2 8PP, United Kingdom

[3] Department of Education & Childhood Studies, Swansea University, Swansea, SA2 8PP, United Kingdom

[4] Department of Nursing and Health Science, Fulda University of Applied Sciences, Fulda, 36037, Germany

[5] Center for Applied Health Science, Leuphana University Lueneburg, Lueneburg, 21335, Germany

[6] Health Literacy, Department of Sport and Health Sciences, Technical University Munich, Uptown München-Campus D, 4. OG, OfficeL412, Georg-Brauchle-Ring 60/62, 80992 München, Germany

[7] Research and Evaluation Division, Public Health Knowledge and Research Directorate, Public Health Wales, 2 Capital Quarter, Cardiff, CF10 4BZ, United Kingdom

[*] Corresponding author: Emily Marchant [1,2], email: E.K.Marchant@swansea.ac.uk

For peer review only - http://bmjopen.bmj.com/site/about/guidelines.xhtml

ABSTRACT

Objectives

Examine if pre-COVID-19 pandemic (prior March 2020) health-related behaviours during primary school are associated with i) being tested for SARS-CoV-2 and ii) testing positive between 1 March 2020 to 31 August 2021.

Design

Retrospective cohort study using an online cohort survey (January 2018 to February 2020) linked to routine PCR SARS-CoV-2 test results.

Setting

Children attending primary schools in Wales (2018-2020), UK who were part of the HAPPEN school network.

Participants

Complete linked records of eligible participants were obtained for n=7,062 individuals. 39.1% (n=2,764) were tested (age 10.6±0.9, 48.9% girls) and 8.1% (n=569) tested positive for SARS-CoV-2 (age 10.6±1.0, 54.5% girls).

Main outcome measures

Logistic regression of health-related behaviours and demographics were used to determine Odds Ratios (OR) of factors associated with i) being tested for SARS-CoV-2 and ii) testing positive for SARS-CoV-2.

For peer review only - http://bmjopen.bmj.com/site/about/guidelines.xhtml

Results

Consuming sugary snacks (1-2 days/week OR=1.24, 95% CI 1.04 – 1.49; 5-6 days/week 1.31, 1.07 – 1.61; reference 0 days) can swim 25m (1.21, 1.06 – 1.39) and age (1.25, 1.16 – 1.35) were associated with an increased likelihood of being tested for SARS-CoV-2. Eating breakfast (1.52, 1.01 – 2.27), weekly physical activity ≥ 60 mins (1-2 days 1.69, 1.04 – 2.74; 3-4 days 1.76, 1.10 – 2.82, reference 0 days), out of school club participation (1.06, 1.02 – 1.10), can ride a bike (1.39, 1.00 – 1.93), age (1.16, 1.05 – 1.28) and girls (1.21, 1.00 – 1.46) were associated with an increased likelihood of testing positive for SARS-CoV-2 (1.16, 1.10 – 1.22).

Conclusions

Associations may be related to parental health literacy and monitoring behaviours. Physically active behaviours may include co-participation with others, and exposure to SARS-CoV-2. A risk versus benefit approach must be considered given the importance of health-related behaviours for development.

STRENGTHS AND LIMITATIONS

- Investigation of the association of pre-pandemic child health-related behaviour measures with subsequent SARS-CoV-2 testing and infection.
- Reporting of multiple child health behaviours linked at an individual-level to routine records of SARS-CoV-2 testing data through the SAIL Databank.

For peer review only - http://bmjopen.bmj.com/site/about/guidelines.xhtml

- Child-reported health behaviours were measured before the COVID-19 pandemic (1 January 2018 to 28 February 2020) which may not reflect behaviours during COVID-19.
- Health behaviours captured through the national-scale HAPPEN survey represent children attending schools that engaged with the HAPPEN Wales primary school network and may not be representative of the whole population of Wales.
- The period of study for PCR-testing for and testing positive for SARS-CoV-2 includes a time frame with varying prevalence rates, approaches to testing children (targeted and mass testing) and restrictions which were not measured in this study.

For peer review only

BACKGROUND

The coronavirus disease 2019 (COVID-19) pandemic caused by severe acute respiratory syndrome coronavirus 2 (SARS-CoV-2) has resulted in widespread disruption to the lives of children across the world, and has contributed to widened inequalities in children's health, wellbeing and education[1,2]. Childhood is a critical developmental period during which health behaviours are established which transcend into adolescence and adulthood[3]. The Organisation for Economic Co-operation and Development (OECD) recognised current trends in children's health, highlighting typical health behaviours of school-aged children that warrant further research in order to better design policies that improve children's health outcomes[4,5]. These include nutrition-related behaviours such as fruit and vegetable intake, consumption of sugary foods and breakfast consumption, physical activity and sedentary behaviours and sleep. The establishment of these health behaviours during childhood are highly influenced by parental mechanisms and monitoring behaviours, particularly in children aged under 12 [6–8].

Whilst evidence has demonstrated the negative impact of the COVID-19 pandemic on children's health-related behaviours including reduced physical activity, increased sedentary behaviour and poorer nutrition[1,9], it is unclear if this association is bidirectional. That is, whether these health behaviours are associated with risk of SARS-CoV-2 infection. Within the adult population, emerging evidence suggests a plausible relationship between pre-pandemic health risk behaviours such as physical inactivity and poor nutrition with SARS-CoV-2 infection and severity of disease[10–13], and increased risk of other infectious diseases[14]. This is attributed to the important role health behaviours play in shaping cardiometabolic health and immune system function. Indeed, research shows links to the early years including critical

For peer review only - http://bmjopen.bmj.com/site/about/guidelines.xhtml

early developmental stages with subsequent risk of developing chronic inflammation, which is associated with non-communicable disease risk and mortality during adulthood [15]. Health behaviours such as adequate nutrient intake[16] and physical activity[17] are required for the regulation and function of the immune system.

As a result, academics have advocated for consideration to be placed on the role of these health behaviours in future endemic/pandemic scenarios[17]. However, research to date has concentrated on adults, explored single health behaviours or examined those with severe COVID-19 infection and hospitalisation[18,19]. The focus of research within the childhood population has principally been placed on clinical outcomes as opposed to lifestyle outcomes, including identifying the clinical characteristics of severe infection, the presence of comorbidities, common symptoms such as cough and clinical biomarkers[20,21]. Whilst serious COVID-19 illness in children is relatively rare, mild or asymptomatic infection is common[22]. Positive SARS-CoV-2 tests require periods of self-isolation, impacting children's physical health and wellbeing, limiting opportunities for children to engage in health-promoting behaviours essential for optimal development such as regular physical activity[9,23]. Therefore, research examining the role of these health behaviours in a childhood population within the context of the COVID-19 pandemic is warranted.

Identifying the pre-pandemic health-related behavioural characteristics of children requiring a SARS-CoV-2 test or testing positive for SARS-CoV-2 infection and hypothesising potential mechanisms through which these may operate, including exposures, socio-demographic and parental influences could yield insight to inform the current COVID-19 pandemic and future pandemic/endemic scenarios. This can also allow targeted intervention to minimise transmission risk that complements national public health measures and

For peer review only

For peer review only - http://bmjopen.bmj.com/site/about/guidelines.xhtml

guidelines, and importantly, mitigates the disruption to children's lives and prevent further exacerbation of pre-existing inequalities, safeguarding their health, wellbeing and education.

In Wales, one of the four nations of the UK, approaches to performing Polymerase Chain Reaction (PCR) tests on children during the period of study included the presence of COVID-19 symptoms, if identified as a close contact to a positive case (e.g. household contacts), or as a follow-up PCR test as encouraged in guidance at the time following a positive Lateral Flow Test (LFT) (e.g. showing symptoms or a close contact and having a positive LFT performed in the home)[24]. Uptake of testing within the childhood population requires parental monitoring behaviours, for example, providing transport to testing facilities and parental health literacy through identification of symptoms.

This study investigates the association of pre-pandemic (prior to 1 March 2020) health-related behaviours self-reported by children aged 8-11 years during primary school before the COVID-19 pandemic between 1 January 2018 and 28 February 2020, with two outcomes; the odds of ever having a SARS-CoV-2 PCR test and the odds of testing positive for SARS-CoV-2 during the period of study. We aim to examine whether these self-reported markers of health-related behaviours reported pre-pandemic are associated with the likelihood of; i) ever being tested for SARS-CoV-2 and ii) ever testing positive for SARS-CoV-2 between 1 March 2020 and 31 August 2021.

For peer review only - http://bmjopen.bmj.com/site/about/guidelines.xhtml

METHODS

Study design

This retrospective cohort study was conducted through the HAPPEN primary school network (*Health and Attainment of Pupils in a Primary Education Network*)[25]. HAPPEN was established in Wales, UK in 2014, following research with headteachers who advocated for increased collaboration to prioritise pupils' health and wellbeing[26,27]. The network brings together primary schools with research and runs up to the current date. School participation in HAPPEN is voluntary and is either once, annually or bi-annually (e.g. to evaluate school-based interventions). Through HAPPEN, children aged 8-11 (years 4 to 6) complete the HAPPEN survey, an online cohort survey that captures a range of validated self-reported health behaviours including physical activity, nutrition and sleep[28]. Retrospective health-related behaviour data were obtained from responses from the HAPPEN survey completed pre-pandemic between 1 January 2018 and 28 February 2020.

These retrospective survey responses were linked with PCR SARS-CoV-2 test results obtained from the Pathology COVID-19 Daily (PATD) routine dataset between 1 March 2020 and 31 August 2021. The PATD dataset contains pillar 1 (swab testing in Public Health England (PHE) labs, NHS Wales labs and NHS hospitals for those with a clinical need, and health and care workers) and pillar 2 (swab testing for the wider population, as set out in government guidance) individual results from PCR tests (negative (suspected), positive (confirmed) for SARS-CoV-2[29]. The period of interest (1 March 2020 to 31 August 2021) includes a time frame of varying approaches to testing children, documented in timeline format in online supplemental appendix 1[29]. This includes targeted (i.e. symptomatic and suspected positive

For peer review only - http://bmjopen.bmj.com/site/about/guidelines.xhtml

case, identified as a close contact of a positive case) and mass testing (i.e. between February 2021 and April 2021 the use of LFTs in the school setting for pupils aged 11 and above (secondary school age) to identify asymptomatic positive cases, with guidance for positive LFTs encouraging follow up PCR tests).

Linkage was performed using the SAIL (*Secure Anonymised Information Linkage*) Databank[30–32]. Data were linked at the individual level using an Anonymous Linkage Field (ALF) to identify participants and link SARS-CoV-2 test results (figure 1). The RECORD checklist[33] for this study is presented in online supplemental appendix 2.

Ethics

Ethical approval was granted by the Swansea University Medical School Research Ethics Committee (2017-0033H). Electronic data (survey responses) were stored in password-protected files only accessible to the research team. The routine data used in this study are available in the SAIL Databank and are subject to review by an independent Information Governance Review Panel (IGRP), to ensure proper and appropriate use of SAIL data. Before any data can be accessed, approval must be received from the IGRP. When access has been approved, it is accessed through a privacy-protecting safe haven and remote access system referred to as the SAIL Gateway. SAIL has established an application process to be followed by anyone who would like to access data. This study has been approved by the SAIL IGRP (project reference: 0911).

For peer review only - http://bmjopen.bmj.com/site/about/guidelines.xhtml

The HAPPEN survey and linked SAIL data

All primary schools (n=1,203) in Wales, UK were invited to participate in the HAPPEN survey between 1 April 2014 and 28 February 2020 via a number of methods including email, social media promotion and through stakeholders in health and education (including local authority health and wellbeing teams, regional education consortia). Prior to 2018, HAPPEN was established in three of the local authorities (total n=22) in Wales. From 2018 to the period of interest, HAPPEN began its expansion to primary schools across Wales. Participating in HAPPEN is voluntary and this study comprises of a convenience sample of children attending n=129 primary schools from 16 local authorities that participated in the HAPPEN survey during the period of interest (1 January 2018 and 28 February 2020). Schools were invited to share details of the survey with parents/guardians (including information sheets). To participate in the HAPPEN survey and link data to routine records, child assent was required in addition to parental consent (between 2014 to 2018) and opt-out parental consent (2019 onwards).

The HAPPEN survey is completed by children aged 8-11 as a self-guided activity within the school setting as a classroom activity with supervision from a teacher/teaching assistant. The survey takes approximately 30 minutes to complete and includes validated self-report measures of typical health behaviours including physical activity, screen time, nutrition, sleep and wellbeing[28]. A full copy of the survey can be found in online supplemental appendix 3 and items, response categories and the coding framework included within analyses in online supplemental appendix 4.

The process of data coding involved two researchers. The first (MJ) cleaned the raw data (including checking for duplicate entries), removed identifiable information and

For peer review only - http://bmjopen.bmj.com/site/about/guidelines.xhtml

generated a unique participant ID number to protect participants' anonymity. The second (EM) researcher coded the anonymised raw dataset using STATA (version 16) to produce a dataset for analyses. This HAPPEN dataset was uploaded to the SAIL Databank, a trusted research environment (TRE) containing individual-level anonymised population-scale data sources about the population of Wales that enables secure data linkage and analysis for research, to be linked with SARS-CoV-2 testing data from the PATD dataset. To link the data, the person-based identifiable data are separated from the survey data and sent to a trusted third party, Digital Health and Care Wales (the national organisation that designs and builds digital services for health and social care in Wales). The survey data is sent to SAIL using a secure file upload. A unique Anonymous Linking Field (ALF) is assigned to the person-based record before it is joined to clinical data via a system linking field. The ALF was used to link records at the individual level between the HAPPEN dataset and PATD dataset containing PCR testing data. This dataset was accessible to authors listed from the Population Data Science group, Swansea University.

Quantitative analysis

The primary outcomes were i) whether the child was PCR tested for the SARS-CoV-2 virus and ii) whether the child had a positive SARS-CoV-2 test between 1 March 2020 and 31 August 2021. Participants were assigned a binary code for SARS-CoV-2 test during period of interest (1: tested at least once for SARS-CoV-2 between 1 March 2020 and 31 August 2021, 0: no evidence of PCR SARS-CoV-2 test) and again for a positive SARS-CoV-2 test during period of interest (1; testing positive for SARS-CoV-2 between 1 March 2020 and 31 August 2021, 0; testing negative, 0; no evidence of PCR test). Participants were assumed to have remained in

For peer review only - http://bmjopen.bmj.com/site/about/guidelines.xhtml

Wales during the period of interest. Eligibility criteria (see cohort flow diagram, Figure 1) within final analyses models were any unique participant with complete linked survey and routine records. Inclusion dates of survey responses for analyses were between 1 January 2018 and 28 February 2020. Multivariable logistic regression analyses, adjusting for confounding variables (sex, age on 1 March 2020, area-level deprivation using the Welsh Index of Multiple Deprivation (WIMD)[34] (version 2019) and clustered by school (to account for differences between schools), determined Odds Ratios (OR) for i) ever being PCR-tested for SARS-CoV-2 virus and ii) ever having a positive PCR SARS-CoV-2 test during the period of interest.

Independent variables as measures of typical pre-pandemic health-related behaviours included within analyses were obtained retrospectively from the HAPPEN survey, completed between 1 January 2018 and 28 February 2020 (online supplemental appendix 4). Health-related behaviour measures included in multivariable analyses are recognised by the OECD as typical health behaviour trends during childhood that warrant research[4,5]. These related to the behaviours from the previous day (ate breakfast, travel actively to and/or from school, number of fruit/vegetables portions consumed, number of times teeth brushed, hours of sleep), frequency of behaviours every day the previous seven days (physically active $\geq$ 60 minutes, sedentary/screen time $\geq$ two hours, felt tired, ate a sugary snack), and general items including participation in number of out of school clubs, can ride a bike and can swim 25 metres. A list of variables included in analyses, coding response categories and coding framework is presented in online supplemental appendix 4. Independent variables were entered concurrently and examined for association with the outcomes i) ever PCR-tested for SARS-CoV-2 and ii) ever tested positive for SARS-CoV-2 between 1 March 2020 and 31 August 2021.

For peer review only - http://bmjopen.bmj.com/site/about/guidelines.xhtml

Figure 1: Cohort flow diagram

Patient and public involvement

The SAIL Databank has a Consumer Panel that provides the public's perspective on data linkage research. The Panel members are involved in all elements of the SAIL Databank process, from developing ideas, advising on bids through approval processes (via the independent Information Governance Review Panel), to disseminating research findings. For more information visit https://saildatabank.com/about-us/public-engagement/.

RESULTS

Survey responses were obtained from n=11,339 participants (figure 1). Survey responses outside the period of interest (before 01 January 2018 and after 28 February 2020) were excluded (n=3,698), followed by duplicate participants (occasions of multiple survey entries, n=579). In the case of duplicates, the most recent instance of survey participation was used. Complete linked unique records of participants meeting eligibility criteria were obtained for n=7,062 individuals. Table 1 presents the descriptive statistics of the study sample by ever PCR-tested for SARS-CoV-2 and ever tested positive for SARS-CoV-2 between 1 March 2020 and 31 August 2021. Of the total sample, 39.1% (n=2,764) were PCR-tested for SARS-CoV-2 and 8.1% (n=569) tested positive for SARS-CoV-2. The mean age on 1 March 2020 (start of period of interest) was 10.6 (± 0.9) for those PCR-tested (table 1) and 10.6 (± 1.0) for those tested positive for SARS-CoV-2 (table 2). Complete case analyses are presented.

For peer review only

For peer review only - http://bmjopen.bmj.com/site/about/guidelines.xhtml

Unadjusted multivariable logistic regression analyses are presented in online supplemental appendix 5.

Table 1: Descriptive statistics of study sample by PCR-tested for SARS-CoV-2 and PCR test positive for SARS-CoV-2 between 1 March 2020 and 31 August 2021.

		**Tested for SARS-CoV-2 n (%)**	**Not tested for SARS-CoV-2 n (%)**	**Tested positive for SARS-CoV-2 n (%)**	**No evidence of positive SARS-CoV-2 test n (%)**
**Sample**		39.1% (2,764)	60.9% (4,298)	8.1% (569)	91.9% (6,498)
**Age at time of HAPPEN survey**		10.1 ± 0.8	9.9 ± 0.9	10.1 ± 0.8	9.9 ± 0.8
**Age on 01/03/2020 (start of period of interest)**		10.6 ± 0.9	10.3 ± 1.1	10.6 ± 1.0	10.4 ± 1.0
**Sex**	**Boy**	49.3% (1,363)	46.7% (2,005)	44.3% (252)	48.0% (3,116)
	**Girl**	48.9% (1,352)	51.8% (2,226)	54.5% (310)	50.3% (3,268)
	Missing	*1.8% (49)*	*1.5% (67)*	1.2% (7)	1.7% (109)
**WIMD 2019 quintiles**	**1 (most deprived)**	24.3% (672)	23.9% (1,025)	28.5% (162)	23.6% (1,535)
	**2**	19.9% (551)	19.02% (826)	19.7% (112)	19.5% (1,265)
	**3**	16.5% (455)	17.4% (748)	17.6% (100)	17.0% (1,103)
	**4**	15.6% (431)	15.8% (678)	14.1% (80)	15.9% (1,029)
	**5 (least deprived)**	18.0% (497)	16.8% (771)	16.5% (94)	17.3% (1,124)
	Missing	*5.7% (158)*	*7.0% (300)*	*3.7% (21)*	*6.7% (5437)*

See online supplemental appendix 4 for variable codebook. Full descriptive statistics table presented in online supplemental appendix 5.

Table 2 presents the multivariable logistic regression for children ever PCR-tested for SARS-CoV-2 between 1 March 2020 and 31 August 2021. The model showed a low goodness-of-fit (R^2=0.02). Children that reported to eat breakfast (OR=1.16, 95% CI 0.99 – 1.36, reference: did not eat breakfast, p<0.1), consume sugary snacks on 1-2 days (1.24, 1.04 – 1.49) and 5-6 days (1.31, 1.07 – 1.61) compared to 0 days, participate in more out of school clubs (1.02,

For peer review only - http://bmjopen.bmj.com/site/about/guidelines.xhtml

For peer review only

212 1.00 – 1.04), able to ride a bike (1.15, 0.98 – 11.35, reference: cannot ride a bike, p<0.1) and
213 able to swim 25m (1.21, 1.06 – 1.39, reference: cannot swim 25m) were more likely to be
214 PCR-tested for SARS-CoV-2. Older children (1.25, 1.16 – 1.35) were also more likely to be PCR-
215 tested for SARS-CoV-2, and compared to quintile 1 (most deprived) those in WIMD quintiles
216 3 (0.85, 0.70 – 1.03, p<0.1) and 5 (0.85, 0.72 – 1.02, p<0.1) were less likely to be PCR-tested
217 for SARS-CoV-2. Unadjusted multivariable logistic regression analyses are presented in online
218 supplemental appendix 6.

219

220 Table 2: Multivariable logistic regression model of significant health behaviour markers and
221 probability of ever being PCR-tested for SARS-CoV-2 between 1 March 2020 and 31 August
222 2021, accounting for baseline age, sex and deprivation, and clustered by school.

**PCR-tested for SARS-CoV-2 (n=6,403, R^2=0.02)**	**OR**	*p* value	**95% CI**
**Ate breakfast**	1.16*	0.067	0.99 – 1.36
Reference: did not eat breakfast	1.00		
**Active travel to school**	0.93	0.339	0.80 – 1.08
Reference: did not active travel to school	1.00		
**Active travel from school**	1.01	0.901	0.86 – 1.19
Reference: did not active travel from school	1.00		
**Number of fruit/vegetable portions**	1.00	0.959	0.97 – 1.03
Reference: 0 fruit/vegetable portions	1.00		
**Number of times teeth brushed**	0.94	0.229	0.86 – 1.04
Reference: did not brush teeth	1.00		
**Sleep hours**	1.01	0.682	0.97 – 1.04
Reference: 0 days physically active ≥ 60 mins (previous seven days)			
**1-2 days physically active ≥ 60 mins**	1.14	0.250	0.91 – 1.41
**3-4 days physically active ≥ 60 mins**	1.13	0.257	0.91 – 1.39
**5-6 days physically active ≥ 60 mins**	1.16	0.217	0.92 – 1.45
**7 days physically active ≥ 60 mins**	1.10	0.451	0.86 – 1.39
Reference: 0 days sedentary ≥ two hours (previous seven days)	1.00		
**1-2 days sedentary ≥ two hours**	1.20	0.141	0.94 – 1.54
**3-4 days sedentary ≥ two hours**	1.18	0.198	0.92 – 1.52
**5-6 days sedentary ≥ two hours**	1.16	0.333	0.86 – 1.56
**7 days sedentary ≥ two hours**	1.16	0.243	0.90 – 1.48

For peer review only - http://bmjopen.bmj.com/site/about/guidelines.xhtml

Reference: 0 days felt tired (previous seven days)	1.00		
**1-2 days felt tired**	0.97	0.686	0.85 – 1.12
**3-4 days felt tired**	1.00	0.963	0.85 – 1.16
**5-6 days felt tired**	1.07	0.528	0.86 – 1.33
**7 days felt tired**	0.97	0.728	0.93 – 1.14
Reference: 0 days consumed sugary snack (previous seven days)	1.00		
**1-2 days consumed sugary snack**	1.24**	0.018	1.04 – 1.49
**3-4 days consumed sugary snack**	1.12	0.301	0.91 – 1.37
**5-6 days consumed sugary snack**	1.31**	0.008	1.07 – 1.61
**7 days consumed sugary snack**	1.16	0.170	0.94 – 1.43
**Number of out of school clubs participation**	1.02*	0.099	1.00 – 1.04
**Can ride a bike**	1.15*	0.086	0.98 – 1.35
Reference: cannot ride a bike	1.00		
**Can swim 25m**	1.21**	0.006	1.06 – 1.39
Reference: cannot swim 25m	1.00		
**Age 01/03/2020**	1.25**	0.000	1.16 – 1.35
**Sex (girl)**	0.92	0.161	0.81 – 1.04
Reference: sex (boy)	1.00		
Reference: WIMD 2019 quintile 1 (most deprived)	1.00		
**WIMD 2019 quintile 2**	0.95	0.600	0.80 – 1.14
**WIMD 2019 quintile 3**	0.85*	0.090	0.70 – 1.03
**WIMD 2019 quintile 4**	0.87	0.131	0.73 – 1.04
**WIMD 2019 quintile 5 (least deprived)**	0.85*	0.078	0.72 – 1.02

OR: Odds Ratio; 95% CI: 95% confidence intervals; **p<0.05, *p<0.1. See online supplemental appendix 4 for variable codebook. Low to moderate correlation between variables (coefficients -0.19 to 0.71).

Table 3 presents the multivariable logistic regression for children ever PCR-tested positive for SARS-CoV-2 between 1 March 2020 and 31 August 2021. Children were more likely to test positive for SARS-CoV-2 if reporting to eat breakfast (OR=1.52, 95% CI 1.01 – 2.27, reference: did not eat breakfast), be physically active for ≥ 60 mins on 1-2 days (1.69, 1.04 – 2.74), 3-4 days (1.76, 1.10 – 2.82) and 5-6 days (1.59, 0.93 – 2.73, p<0.1) compared to 0 days, participate in more out of school clubs (1.06, 1.02 – 1.10) and able to ride a bike (1.39, 1.00 – 1.93, reference: cannot ride a bike). Older children (1.16, 1.05 – 1.28) were more likely to test positive for SARS-CoV-2. Compared to boys, girls were more likely to test positive (1.21, 1.00

For peer review only - http://bmjopen.bmj.com/site/about/guidelines.xhtml

– 1.46), and compared to the most deprived quintile 1, those living in the least deprived quintiles 4 (0.64, 0.46 – 0.90) and 5 (0.64, 0.46 – 0.89) were less likely to test positive for SARS-CoV-2. The model showed a low goodness-of-fit (R^2=0.02). Unadjusted multivariable logistic regression analyses are presented in online supplemental appendix 6.

Table 3: Multivariable logistic regression model of significant health behaviour markers and probability of ever PCR-testing positive for SARS-CoV-2 between 1 March 2020 and 31 August 2021, accounting for baseline age, sex and deprivation, and clustered by school.

**PCR test positive for SARS-CoV-2 (n=6,403, R^2=0.02)**	**OR**	***p* value**	**95% CI**
**Ate breakfast**	1.52**	0.043	1.01 – 2.27
Reference: did not eat breakfast	1.00		
**Active travel to school**	0.91	0.481	0.70 – 1.18
Reference: did not active travel to school	1.00		
**Active travel from school**	0.98	0.910	0.72 – 1.33
Reference: did not active travel from school	1.00		
**Number of fruit/vegetable portions**	0.98	0.461	0.94 – 1.03
Reference: 0 fruit/vegetable portions	1.00		
**Number of times teeth brushed**	1.05	0.542	0.90 – 1.21
Reference: did not brush teeth	1.00		
**Sleep hours**	0.97	0.345	0.92 – 1.03
Reference: 0 days physically active ≥ 60 mins (previous seven days)	1.00		
**1-2 days physically active ≥ 60 mins**	1.69**	0.035	1.04 – 2.74
**3-4 days physically active ≥ 60 mins**	1.76**	0.018	1.10 – 2.82
**5-6 days physically active ≥ 60 mins**	1.59*	0.091	0.93 – 2.73
**7 days physically active ≥ 60 mins**	1.50	0.158	0.85 – 2.65
Reference: 0 days sedentary ≥ two hours (previous seven days)	1.00		
**1-2 days sedentary ≥ two hours**	0.96	0.847	0.63 – 1.47
**3-4 days sedentary ≥ two hours**	0.94	0.789	0.59 – 1.50
**5-6 days sedentary ≥ two hours**	0.93	0.803	0.51 – 1.68
**7 days sedentary ≥ two hours**	1.02	0.946	0.63 – 1.65
Reference: 0 days felt tired (previous seven days)	1.00		
**1-2 days felt tired**	1.17	0.207	0.91 – 1.51
**3-4 days felt tired**	1.17	0.232	0.91 – 1.50
**5-6 days felt tired**	1.19	0.243	0.89 – 1.60
**7 days felt tired**	0.89	0.390	0.68 – 1.16

For peer review only

For peer review only - http://bmjopen.bmj.com/site/about/guidelines.xhtml

Reference: 0 days consumed sugary snack (previous seven days)	1.00		
**1-2 days consumed sugary snack**	1.13	0.523	0.77 – 1.65
**3-4 days consumed sugary snack**	1.06	0.783	0.70 – 1.61
**5-6 days consumed sugary snack**	1.36	0.159	0.89 – 2.08
**7 days consumed sugary snack**	1.08	0.727	0.71 – 1.63
**Number of out of school clubs participation**	1.06**	0.002	1.02 – 1.10
**Can ride a bike**	1.39**	0.049	1.00 – 1.93
Reference: cannot ride a bike	1.00		
**Can swim 25m**	1.14	0.324	0.88 – 1.48
Reference: cannot swim 25m			
**Age 01/03/2020**	1.16**	0.003	1.05 – 1.28
**Sex (girl)**	1.21**	0.046	1.00 – 1.46
Reference: sex (boy)	1.00		
Reference: WIMD 2019 quintile 1 (most deprived)	1.00		
**WIMD 2019 quintile 2**	0.79	0.113	0.59 – 1.06
**WIMD 2019 quintile 3**	0.79	0.128	0.59 – 1.07
**WIMD 2019 quintile 4**	0.64**	0.009	0.46 – 0.90
**WIMD 2019 quintile 5**	0.64**	0.007	0.46 – 0.89

OR: Odds Ratio; 95% CI: 95% confidence intervals; **p<0.05, *p<0.1. See online supplemental appendix 4 for variable codebook. Low to moderate correlation between variables (coefficients -0.19 to 0.71).

DISCUSSION

This study examines whether markers of health-related behaviours reported by primary school-aged children between January 2018 and February 2020 are associated with the likelihood of being PCR-tested for SARS-CoV-2 and testing positive between 1 March 2020 and 31 August 2021. Findings suggest that reporting to eat breakfast, weekly sugary snack consumption (both low and high), participating in more out of school clubs, being able to ride a bike and being able to swim 25 metres were associated with an increased likelihood of being tested for SARS-CoV-2. Health behaviours associated with an increased likelihood of testing positive for SARS-CoV-2 were eating breakfast, engaging in higher weekly physical activity,

For peer review only - http://bmjopen.bmj.com/site/about/guidelines.xhtml

participating in more out of school clubs and riding a bike. Boys were more likely to test positive for SARS-CoV-2 than girls, and those living in a less deprived area less likely to test positive that those residing in the most deprived area.

This study encompasses a period of both targeted and mass PCR testing, and detecting child positive cases using routine PCR testing data in this study requires a parent/guardian to take the child for testing. We find associations between child-reported health-related behaviours with both PCR-testing for SARS-CoV-2 and testing positive for SARS-CoV-2. Through this, we theorise that because health behaviours are largely guided and facilitated by parents, our associations are likely to be reflecting health literacy among parents, along with monitoring behaviours. In the case of symptomatic testing, the detection of positive child cases relies on parents recognising symptoms and communication with their child. For asymptomatic testing through the use of LFT (e.g. asymptomatic school testing between February and April 2021), guidance encouraged positive LFTs to be followed up with PCR-testing, requiring knowledge of how to access testing services and ability to access services (e.g. transport). These behaviours form a level of health literacy, recognised as the ability to access, understand, interpret and apply medical information and make informed decisions regarding medical advice, issues or guidelines[35]. Parental health literacy impacts the decision a parent makes relating to their child[36] and is correlated with a number of health indicators including knowledge of health and health services, and the parent and child engaging in health-promoting behaviours[8,35].

Parenting is an important contributor to promoting positive health behaviours in children, and is represented by a constellation of attitudes, behaviours and values for the child. The presence of multiple physically active behaviours represented by the association of being able to swim, ride a bike and participation in more out of school clubs may represent

For peer review only

For peer review only - http://bmjopen.bmj.com/site/about/guidelines.xhtml

underlying parental involvement and modelling behaviour, including involvement in leisure time activities, providing financial and transport provision to attend organised activities such as access to swimming lessons and the provision of equipment[7]. This may also have a socioeconomic component, building on the ideas of Bourdieu in terms of social capital, and access health enhancing material items[37].

Diet-related findings of eating breakfast and restrictive weekly sugary snack consumption (1-2 days per week) may indicate higher parental monitoring, supporting our theory. In comparison, higher weekly sugary snack consumption (5-6 days per week) may represent less restrictive parental monitoring and more autonomy and choice for the child. We posit that as parental behaviours are often driven by underlying styles of parenting[38], the associations could be depicting varying levels of control; for instance, those snacking 1-2 times perhaps have parents with greater control, vs. those snacking 5-6 times with parents with less controlling styles. This theory may well transcend into other behaviours, including limits and freedom in socialising with others, placing a greater risk for infection of illness – including COVID-19.

While evidence recognises the importance of adequate nutrition[16] and physical activity[17] for cardiometabolic health and immune system function, the findings in the current study draw attention to another potential mechanism of increased contacts and exposure to SARS-CoV-2. Engagement in physically active behaviours such as out of school clubs, higher frequency of physically active days in a week and riding a bike may increase the number of social contacts of the child. Indeed, there is a wealth of evidence demonstrating that childhood physical activity participation is highly influenced by their social environment and co-participation with peers[39]. It is therefore possible that physically active children had

For peer review only

For peer review only - http://bmjopen.bmj.com/site/about/guidelines.xhtml

increased social contacts and exposure to SARS-CoV-2 through co-participation of activity and play opportunities.

However, it is important to note that physical activity is an essential health behaviour required for optimal development and a range of health and wellbeing outcomes. These findings must be considered in balance with the importance of encouraging these behaviours and providing physically active opportunities during childhood. This viewpoint was also reflected in Government guidance and risk assessments during the COVID-19 pandemic through the reopening of children's playgrounds and outdoor play spaces, with explicit reference to outdoor play and physical activity as fundamental for children's development and wellbeing[40].

Contact patterns may also explain sex differences observed in this study, as we found girls are more likely to test positive for SARS-CoV-2. In addition to age assortative mixing patterns of children, there is a developmental tendency by children to socially interact with members of the same sex and engage in gender-typed activity[41]. For girls, the location of play preferences are more likely to be indoors and in contact with supervising adults, where exposure to SARS-CoV-2 is possibly greater[42]. The findings of association between increasing age and likelihood of testing positive for SARS-CoV-2 in this study are supported by wider literature which suggests increasing susceptibility of infection in the adolescent age group compared to younger than 10 to 14 years[43].

Our findings also show an area-level social gradient. Those living in the least deprived WIMD quintiles 4 and 5 were less likely to test positive for SARS-CoV-2 compared to the most deprived quintile, which may reflect deprivation-related exposure patterns to SARS-CoV-2. Indeed, research conducted using the WIMD and English area-level deprivation indictors found adults living in the most deprived areas demonstrated differential exposures to SARS-

For peer review only

For peer review only - http://bmjopen.bmj.com/site/about/guidelines.xhtml

CoV-2[44]. This included patterns of public activities such as attending work or education outside of the household, using public transport and car sharing with non-household members. This, and considerations of the deprivation-related disparities in the built environment including access to open spaces highlight the inequalities that persist in risk of SARS-CoV-2 infection. Furthermore, whilst it is likely that children mix with others from similar demographic areas, the finding in our study may also reflect community prevalence which was not captured.

For peer review only

CONCLUSION

We theorise that health-promoting behaviours associated with a child being tested for SARS-CoV-2 and being identified as positive may be a proxy of higher parental health literacy and monitoring behaviours. Furthermore, co-participation in physically active behaviours with peers may increase exposure to SARS-CoV-2. This must be considered from a risk versus benefit approach in relation to the importance of physically active behaviours for children's development and wellbeing.

Acknowledgements

The authors would like to thank all participating primary schools and pupils that took part in this study. This work was supported by the National Centre for Population Health and Wellbeing Research through the HAPPEN network. This study makes use of anonymised data

held in the Secure Anonymised Information Linkage (SAIL) Databank. We would like to acknowledge all the data providers who make anonymised data available for research. We would also like to thank Dr Annemarie Docherty and Dr Olivia Swann from The University of Edinburgh for providing informal peer review input to the final draft.

Availability for data and materials

The routine data used in this study are available in the SAIL Databank at Swansea University, Swansea, UK. All proposals to use SAIL data are subject to review by an IGRP. Before any data can be accessed, approval must be given by the IGRP. The IGRP gives careful consideration to each project to ensure proper and appropriate use of SAIL data. When access has been approved, it is gained through a privacy-protecting safe haven and remote access system referred to as the SAIL Gateway. SAIL has established an application process to be followed by anyone who would like to access data via SAIL https://www.saildatabank.com/application-process. This study has been approved by the IGRP as project 0911.

For peer review only

Funding

The Economic and Social Research Council (ESRC) funded the development of the HAPPEN network (grant number: ES/J500197/1) which this research was conducted through. The National Centre for Population Health and Wellbeing Research (NCPHWR) funded by Health and Care Research Wales provided infrastructural support for this work. This work was supported by the Con-COV team funded by the Medical Research Council (grant number: MR/V028367/1). This work was supported by Health Data Research UK, which receives its

For peer review only - http://bmjopen.bmj.com/site/about/guidelines.xhtml

funding from HDR UK Ltd (HDR-9006) funded by the UK Medical Research Council, Engineering and Physical Sciences Research Council, Economic and Social Research Council, Department of Health and Social Care (England), Chief Scientist Office of the Scottish Government Health and Social Care Directorates, Health and Social Care Research and Development Division (Welsh Government), Public Health Agency (Northern Ireland), British Heart Foundation (BHF) and the Wellcome Trust. This work was a collaboration with the ADR Wales programme of work. ADR Wales is part of the Economic and Social Research Council (part of UK Research and Innovation) funded ADR UK (grant ES/S007393/1). This work was supported by the Wales COVID-19 Evidence Centre, funded by Health and Care Research Wales and by the COVID-19 Longitudinal Health and Wellbeing National Core Study funded by the Medical Research Council (MC_PC_20030).

Transparency statement

The lead author affirms that the manuscript is an honest, accurate, and transparent account of the study being reported; that no important aspects of the study have been omitted; and that any discrepancies from the study as originally planned have been explained.

Competing interests

The authors declare that they have no competing interests.

For peer review only

For peer review only - http://bmjopen.bmj.com/site/about/guidelines.xhtml

Contributorship statement

EM and SB conceptualised the study design. EM and MJ acquired the data, and EM and JK were responsible for data curation. EM performed the statistical analysis, undertook the initial interpretation of the data and wrote the initial draft. EL and SB contributed to the writing of the manuscript and provided statistical guidance. EL, JK, SB, LC and RL provided critical interpretation of the data. The manuscript was critically reviewed and edited by EL, TC, LG, RF, KD, OO, MJ, LC, FT, JK, AA, RL and SB. SB provided supervision and TC and LG provided mentorship. EM is the guarantor. AD and OS critically reviewed the initial manuscript. EM, EL, TC, LG, RF, KD, OO, MJ, LC, FT, JK, AA, RL and SB approved the final manuscript and agreed to be accountable for all aspects of the work.

References

1 James M, Marchant E, Defeyter MA, *et al.* Impact of school closures on the health and well-being of primary school children in Wales UK: a routine data linkage study using the HAPPEN Survey (2018-2020). *BMJ Open* 2021;**11**. doi:10.1136/BMJOPEN-2021-051574

2 Marchant E, Todd C, James M, *et al.* Primary school staff perspectives of school closures due to COVID-19, experiences of schools reopening and recommendations for the future: A qualitative survey in Wales. *PLoS One* 2021;**16**:e0260396. doi:10.1371/JOURNAL.PONE.0260396

3 Craigie AM, Lake AA, Kelly SA, *et al.* Tracking of obesity-related behaviours from

For peer review only

childhood to adulthood: A systematic review. *Maturitas* 2011;**70**:266–84. doi:10.1016/j.maturitas.2011.08.005

4 OECD. ‘Are children active and physically healthy?’,. In: *Measuring What Matters for Child Well-Being and Policies*. Paris: : OECD Publishing 2021. 1–295. doi:10.1787/e82fded1-en

5 OECD. Education in the Digital Age. In: Burns T, Gottschalk F, eds. *Education in the Digital Age*. OECD 2020. 1–218. doi:10.1787/1209166A-EN

6 Pyper E, Harrington D, Manson H. The impact of different types of parental support behaviours on child physical activity, healthy eating, and screen time: a cross-sectional study. Published Online First: 2016. doi:10.1186/s12889-016-3245-0

7 Edwardson CL, Gorely T. Parental influences on different types and intensities of physical activity in youth: A systematic review. *Psychol Sport Exerc* 2010;**11**:522–35. doi:10.1016/j.psychsport.2010.05.001

8 Pawellek M, Kopf FM, Egger N, *et al.* Pathways linking parental health literacy with health behaviours directed at the child: a scoping review. *Health Promot Int* 2021;**2021**:1–29. doi:10.1093/HEAPRO/DAAB154

9 Moore SA, Faulkner G, Rhodes RE, *et al.* Impact of the COVID-19 virus outbreak on movement and play behaviours of Canadian children and youth: A national survey. *Int J Behav Nutr Phys Act* 2020;**17**. doi:10.1186/s12966-020-00987-8

10 Chowdhury R, Van Daalen KR, Franco OH. Cardiometabolic Health: Key in Reducing Adverse COVID-19 Outcomes. *Glob Heart* 2020;**15**:58. doi:10.5334/gh.879

11 Koch R, Jordan S, Starker A, *et al.* Health behaviour and COVID-19: Initial findings on the pandemic Journal of Health Monitoring 2. *J Heal Monit* 2020;**2020**:1–14. doi:10.25646/7055

For peer review only

For peer review only - http://bmjopen.bmj.com/site/about/guidelines.xhtml

12 Childs CE, Calder PC, Miles EA. Diet and Immune Function. *Nutrients* 2019;**11**. doi:10.3390/nu11081933

13 Lange KW, Nakamura Y. Lifestyle factors in the prevention of COVID-19. *Glob Heal J* 2020;**4**:146–52. doi:10.1016/J.GLOHJ.2020.11.002

14 Chastin SFM, Abaraogu U, Bourgois JG, *et al.* Effects of Regular Physical Activity on the Immune System, Vaccination and Risk of Community-Acquired Infectious Disease in the General Population: Systematic Review and Meta-Analysis. *Sports Med* 2021;**51**:1673. doi:10.1007/S40279-021-01466-1

15 Furman D, Campisi J, Verdin E, *et al.* Chronic inflammation in the etiology of disease across the life span. *Nat Med 2019 2512* 2019;**25**:1822–32. doi:10.1038/s41591-019-0675-0

16 Wintergerst ES, Maggini S, Hornig DH. Contribution of selected vitamins and trace elements to immune function. *Ann Nutr Metab* 2007;**51**:301–23. doi:10.1159/000107673

17 Laddu DR, Lavie CJ, Phillips SA, *et al.* Physical activity for immunity protection: Inoculating populations with healthy living medicine in preparation for the next pandemic. *Prog Cardiovasc Dis* 2021;**64**:102–4. doi:10.1016/J.PCAD.2020.04.006

18 Hamer M, Kivimäki M, Gale C, *et al.* Lifestyle risk factors, inflammatory mechanisms, and COVID-19 hospitalization: A community-based cohort study of 387,109 adults in UK. *Brain Behav Immun* 2020;**87**:184–7. doi:10.1016/J.BBI.2020.05.059

19 Lee SW, Lee J, Moon SY, *et al.* Physical activity and the risk of SARS-CoV-2 infection, severe COVID-19 illness and COVID-19 related mortality in South Korea: a nationwide cohort study. *Br J Sports Med* 2021;**0**:18. doi:10.1136/BJSPORTS-2021-104203

20 Zhou B, Yuan Y, Wang S, *et al.* Risk profiles of severe illness in children with COVID-19:

For peer review only

For peer review only - http://bmjopen.bmj.com/site/about/guidelines.xhtml

a meta-analysis of individual patients. *Pediatr Res 2021 902* 2021;**90**:347–52. doi:10.1038/s41390-021-01429-2

21 Shekerdemian LS, Mahmood NR, Wolfe KK, *et al.* Characteristics and Outcomes of Children With Coronavirus Disease 2019 (COVID-19) Infection Admitted to US and Canadian Pediatric Intensive Care Units. *JAMA Pediatr* 2020;**174**:868–73. doi:10.1001/JAMAPEDIATRICS.2020.1948

22 Castagnoli R, Votto M, Licari A, *et al.* Severe Acute Respiratory Syndrome Coronavirus 2 (SARS-CoV-2) Infection in Children and Adolescents: A Systematic Review. *JAMA Pediatr* 2020;**174**:882–9. doi:10.1001/JAMAPEDIATRICS.2020.1467

23 OECD. Combatting COVID-19's effect on children - OECD. 2020.https://www.oecd.org/coronavirus/policy-responses/combatting-covid-19-s-effect-on-children-2e1f3b2f/ (accessed 1 Oct 2021).

24 Senedd Research. Coronavirus timeline: Welsh and UK governments' response – IN BRIEF. 2020.https://seneddresearch.blog/2020/03/19/covid-19-timeline-welsh-and-uk-governments-response/ (accessed 30 Mar 2022).

25 Todd, C., Christian, D., Tyler, R., Stratton, G., Brophy S. Developing HAPPEN (Health and Attainment of Pupils involved in a Primary Education Network): working in partnership to improve child health and education. *Perspect Public Health* 2016;**136**:115–6.

26 Christian D, Todd C, Davies H, *et al.* Community led active schools programme (CLASP) exploring the implementation of health interventions in primary schools: headteachers' perspectives. *BMC Public Health* 2015;**15**:238. doi:10.1186/s12889-015-1557-0

27 Todd C, Christian D, Davies H, *et al.* Headteachers' prior beliefs on child health and their engagement in school based health interventions: a qualitative study. *BMC Res Notes*

For peer review only

For peer review only - http://bmjopen.bmj.com/site/about/guidelines.xhtml

2015;**18**:161. doi:10.1186/s13104-015-1091-2

28 Everson B, Mackintosh KA, McNarry MA, *et al.* Can Wearable Cameras be Used to Validate School-Aged Children's Lifestyle Behaviours? *Children* 2019;**6**:20. doi:10.3390/children6020020

29 Department of Health & Social Care. COVID-19 testing data: methodology note - GOV.UK. 2020.https://www.gov.uk/government/publications/coronavirus-covid-19-testing-data-methodology/covid-19-testing-data-methodology-note (accessed 30 Mar 2022).

30 Ford D V., Jones KH, Verplancke JP, *et al.* The SAIL Databank: Building a national architecture for e-health research and evaluation. *BMC Health Serv Res* 2009;**9**. doi:10.1186/1472-6963-9-157

31 Jones KH, Ford DV, Thompson S, *et al.* A Profile of the SAIL Databank on the UK Secure Research Platform. *Int J Popul Data Sci* 2019;**4**. doi:10.23889/ijpds.v4i2.1134

32 Lyons J, Akbari A, Torabi F, *et al.* Understanding and responding to COVID-19 in Wales: protocol for a privacy-protecting data platform for enhanced epidemiology and evaluation of interventions. *BMJ Open* 2020;**10**:43010. doi:10.1136/bmjopen-2020-043010

33 Benchimol EI, Smeeth L, Guttmann A, *et al.* The REporting of studies Conducted using Observational Routinely-collected health Data (RECORD) Statement. *PLOS Med* 2015;**12**:e1001885. doi:10.1371/JOURNAL.PMED.1001885

34 Welsh Government. Welsh Index of Multiple Deprivation. 2019.https://gov.wales/welsh-index-multiple-deprivation-full-index-update-ranks-2019

35 Sørensen K, Van den Broucke S, Fullam J, *et al.* Health literacy and public health: A

For peer review only

For peer review only - http://bmjopen.bmj.com/site/about/guidelines.xhtml

systematic review and integration of definitions and models. Published Online First: 2012. doi:10.1186/1471-2458-12-80

36 Morrison AK, Glick A, Yin HS. Health Literacy: Implications for Child Health. *Pediatr Rev* 2019;**40**:263–77. doi:10.1542/PIR.2018-0027

37 Bourdieu P. The Forms of Capital. In: *Handbook of Theory and Research for the Sociology of Education*. New York: : Greenwood 1986. 241–58.

38 Darling N, Steinberg L. Parenting Style as Context: An Integrative Model. *Psychol Bull* 1993;**113**:487–96. doi:10.1037/0033-2909.113.3.487

39 Li L, Moosbrugger ME. Correlations between Physical Activity Participation and the Environment in Children and Adolescents: A Systematic Review and Meta-Analysis Using Ecological Frameworks. *Int J Environ Res Public Heal 2021, Vol 18, Page 9080* 2021;**18**:9080. doi:10.3390/IJERPH18179080

40 Welsh Government. Reopening children's playgrounds and outdoor play areas: coronavirus. Guidance for those in charge of playgrounds or outdoor play areas to keep children safe during the coronavirus pandemic. 2020. https://gov.wales/reopening-childrens-playgrounds-and-outdoor-play-areas-coronavirus-html (accessed 25 Apr 2022).

41 Mehta CM, Strough JN. Sex segregation in friendships and normative contexts across the life span. *Dev Rev* 2009;**29**:201–20. doi:10.1016/J.DR.2009.06.001

42 Edwards CP, Knoche L, Kumru A. Play Patterns and Gender. *Encycl women Gend Sex similarities Differ impact Soc Gend* 2001;:809–15.http://digitalcommons.unl.edu/psychfacpub/607/ (accessed 25 Apr 2022).

43 Viner RM, Mytton OT, Bonell C, *et al.* Susceptibility to SARS-CoV-2 Infection among Children and Adolescents Compared with Adults: A Systematic Review and Meta-

For peer review only

For peer review only - http://bmjopen.bmj.com/site/about/guidelines.xhtml

analysis. JAMA Pediatr. 2020. doi:10.1001/jamapediatrics.2020.4573

44 Beale S, Braithwaite I, Navaratnam AM, *et al.* Deprivation and exposure to public activities during the COVID-19 pandemic in England and Wales. *J Epidemiol Community Heal* 2022;**76**:319–26. doi:10.1136/jech-2021-217076

Figure 1: Cohort flow diagram

For peer review only

For peer review only - http://bmjopen.bmj.com/site/about/guidelines.xhtml

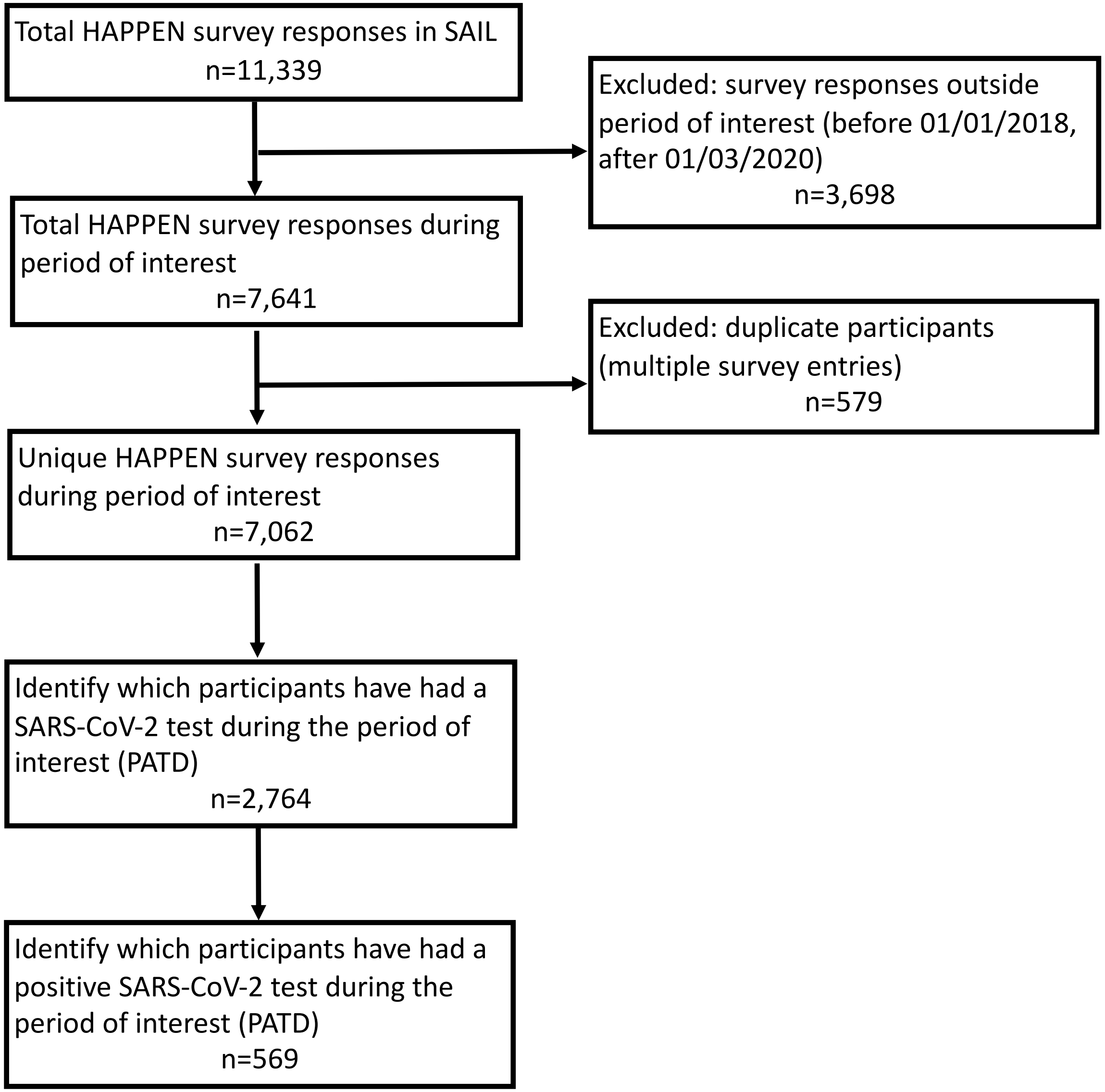
Total HAPPEN survey responses in SAIL
n=11,339
Excluded: survey responses outside period of interest (before 01/01/2018, after 01/03/2020)
n=3,698
Total HAPPEN survey responses during period of interest
n=7,641
Excluded: duplicate participants (multiple survey entries)
n=579
Unique HAPPEN survey responses during period of interest
n=7,062
Identify which participants have had a SARS-CoV-2 test during the period of interest (PATD)
n=2,764
Identify which participants have had a positive SARS-CoV-2 test during the period of interest (PATD)
n=569

Online supplemental appendix 1: see [24]

Date	Event
1 March 2020	Start of study period.
20 March 2020	All schools across Wales closed, with the exception of provision of vulnerable children and children of critical workers.
28 April 2020	More drive-through testing centres.
13 May 2020	Test, Trace, Protect published including testing strategy to expand from testing workers in hospitals and care comes to symptomatic community testing.
18 May 2020	Home testing rolled out, enabling symptomatic people to request home coronavirus test.
1 June 2020	Contract tracing began in Wales. Anyone who tested positive for coronavirus contacted by contact tracer and asked to provide details of everyone they had been in close contact with. Those close contacts contacted and asked to self-isolate for 14 days.
29 June 2020	Schools opened to pupils from all year groups for limited periods during the week, with only a third of pupils in school at any one time.
15 July 2020	New Wales coronavirus testing strategy released.
17–24 July 2020	All schools in Wales closed for the summer holidays.
18 August 2020	Further investment in testing. Welsh Government announced £32 million funding to improve coronavirus testing including speed of processing tests and ensuring robust testing and contract tracing system for anticipated second wave.
1 September 2020	Some schools operated a phased return with flexibility to priority groups.
14 September 2020	Schools opened to all pupils in Wales.
17 September 2020	Testing update. The Health Minister provided an update on Wales' response to current challenges with coronavirus testing, including a significant increase in demand for testing.
24 September 2020	NHS COVID-19 app launched for people aged 16 and over, including increased capacity for identifying contacts of those tested positive for coronavirus.
29 September 2020	Wales' Minister for Health and Social Care sets out the prioritisation for Covid-19 testing in Wales as the Welsh Government move into a new phase of its response. The Minster set out six priorities for testing, with those working in education or childcare settings the fifth priority group and all symptomatic individuals being sixth.
19–30 October 2020	Autumn half term holiday (three local authorities had 2 week half term)
2 November 2020	Pupils in year 9 and above not expected to be present in school due to firebreak in Wales.
16 November 2020	New financial support scheme launched for people who need to self-isolate due to a positive coronavirus test result or those asked to do so by NHS Wales Test, Trace, Protect.
18 November 2020	Merthyr Tydfil (one of 22 local authorities) to be first whole area testing pilot in Wales. Everyone offered Covid-19 testing, whether symptomatic or asymptomatic. The mass testing programme used LFTs.
27 November 2020	Mass testing extended (use of LFTs).
14 December 2020	All secondary schools in Wales moved to online remove learning for last week of term before Christmas (14-18 December 2020). Many primary schools also closed.
14 December 2020	Plans for serial testing in schools. The Welsh Government announces plans to roll out serial testing in schools and colleges from January 2021.
18 December 2020	Testing infrastructure developments to support mass testing of symptomatic people across the Welsh population. In addition to increasing testing infrastructure for people with symptoms, also developing approach and support for people without symptoms to access lateral flow tests (LFT).

For peer review only

Online supplemental appendix 1: see [24]

4 January 2021	All schools across Wales closed and moved to online remote learning, with the exception of provision for vulnerable children and children of critical workers.
28 January 2021	Publication of an updated coronavirus testing strategy including to continue to test symptomatic individuals.
1 February 2021	Users of NHS Covid-19 app could apply for self-isolation payment. People on low incomes who are asked to self-isolate via the NHS Covid-19 are eligible to apply for the £500 self-isolation support payment, alongside those who have been asked to self-isolate by the Test, Trace, Protect service and parents/carers whose child has been asked to self-isolate by their education setting.
5 February 2021	Twice weekly testing in schools. The Health Minister announces that daily contact testing in schools and colleges will be paused and instead, twice weekly testing using LFTs. Guidance for detection of positive LFT encourages follow up PCR test.
27 February 2021	Testing offer extended to upper secondary and college learners. Welsh Government Ministers announce that they are extending the offer of regular, twice weekly, LFTs at home to all those of upper secondary age. This will start with offering tests to years 11 to 13, and to all further education college learners and those on work-based apprenticeship and traineeship programmes.
10 March 2021	Testing for close contacts of positive cases. Wales' Health Minister, announces that people who are close contacts of someone who has tested positive for coronavirus and have been asked to isolate by contact tracers will now be offered a coronavirus test. Also extra £50 million to allow health boards to extend contact tracing over the summer.
15 March 2021	All remaining primary school pupils and secondary pupils in qualification years 11 and 13 able to return to learning on site. Other secondary years were able to return for check ins.
15 March 2021	Voluntary asymptomatic testing offer available to secondary age pupils in years 10 and above.
22 March 2021	Community asymptomatic testing programme extended to end of September 2021.
30 March 2021	Updated testing strategy published.
12 April 2021	All remaining pupils were able to return to learning on site.
13 April 2021	Voluntary asymptomatic testing offer extended to all secondary school age years (years 7 and above).
26 May 2021	Covid tests (LFTs) encouraged for people holidaying in Wales.
31 August 2021	End of study period.

Online supplemental appendix 2: RECORD statement

The RECORD statement – checklist of items, extended from the STROBE statement, that should be reported in observational studies using routinely collected health data.

	Item No.	STROBE items	Location in manuscript where items are reported	RECORD items	Location in manuscript where items are reported
**Title and abstract**					
	1	(a) Indicate the study's design with a commonly used term in the title or the abstract (b) Provide in the abstract an informative and balanced summary of what was done and what was found		RECORD 1.1: The type of data used should be specified in the title or abstract. When possible, the name of the databases used should be included.<br><br>RECORD 1.2: If applicable, the geographic region and timeframe within which the study took place should be reported in the title or abstract.<br><br>RECORD 1.3: If linkage between databases was conducted for the study, this should be clearly stated in the title or abstract.	1.1: Title and abstract (page 1-2)<br><br>1.2: Title and abstract (page 1-2)
**Introduction**					
Background rationale	2	Explain the scientific background and rationale for the investigation being reported			Background (page 5-7)
Objectives	3	State specific objectives, including any prespecified hypotheses			Background (page 5-7)
**Methods**					
Study Design	4	Present key elements of study design early in the paper			Methods - Study design (page 7)
Setting	5	Describe the setting, locations, and relevant dates, including			Methods - Study design (page 7)

For peer review only

Online supplemental appendix 2: RECORD statement

		periods of recruitment, exposure, follow-up, and data collection			
Participants	6	*(a) Cohort study* - Give the eligibility criteria, and the sources and methods of selection of participants. Describe methods of follow-up<br>*Case-control study* - Give the eligibility criteria, and the sources and methods of case ascertainment and control selection. Give the rationale for the choice of cases and controls<br>*Cross-sectional study* - Give the eligibility criteria, and the sources and methods of selection of participants<br><br>*(b) Cohort study* - For matched studies, give matching criteria and number of exposed and unexposed<br>*Case-control study* - For matched studies, give matching criteria and the number of controls per case		RECORD 6.1: The methods of study population selection (such as codes or algorithms used to identify subjects) should be listed in detail. If this is not possible, an explanation should be provided.<br><br>RECORD 6.2: Any validation studies of the codes or algorithms used to select the population should be referenced. If validation was conducted for this study and not published elsewhere, detailed methods and results should be provided.<br><br>RECORD 6.3: If the study involved linkage of databases, consider use of a flow diagram or other graphical display to demonstrate the data linkage process, including the number of individuals with linked data at each stage.	6.1: Figure 1: Cohort Flow Diagram (page 12)<br><br>6.3: Figure 1: Cohort Flow Diagram (page 12)
Variables	7	Clearly define all outcomes, exposures, predictors, potential confounders, and effect modifiers. Give diagnostic criteria, if applicable.		RECORD 7.1: A complete list of codes and algorithms used to classify exposures, outcomes, confounders, and effect modifiers should be provided. If these cannot be reported, an explanation should be provided.	7.1: Supplemental appendix 4: HAPPEN survey variable codebook
Data sources/ measurement	8	For each variable of interest, give sources of data and details of methods of assessment (measurement).			Methods - The HAPPEN survey and linked SAIL data (Page 8-9)

For peer review only

Online supplemental appendix 2: RECORD statement

		Describe comparability of assessment methods if there is more than one group			
Bias	9	Describe any efforts to address potential sources of bias			Methods - Quantitative analysis (page 9)
Study size	10	Explain how the study size was arrived at			Figure 1: Cohort flow diagram (page 12)
Quantitative variables	11	Explain how quantitative variables were handled in the analyses. If applicable, describe which groupings were chosen, and why			Methods - Quantitative analysis (page 11)
Statistical methods	12	(a) Describe all statistical methods, including those used to control for confounding<br>(b) Describe any methods used to examine subgroups and interactions<br>(c) Explain how missing data were addressed<br>(d) *Cohort study* - If applicable, explain how loss to follow-up was addressed<br>*Case-control study* - If applicable, explain how matching of cases and controls was addressed<br>*Cross-sectional study* - If applicable, describe analytical methods taking account of sampling strategy<br>(e) Describe any sensitivity analyses			Methods - Quantitative analysis (page 11)

For peer review only

Online supplemental appendix 2: RECORD statement

Data access and cleaning methods		..		RECORD 12.1: Authors should describe the extent to which the investigators had access to the database population used to create the study population.<br><br>RECORD 12.2: Authors should provide information on the data cleaning methods used in the study.	12.1: Methods - The HAPPEN survey and linked SAIL data (page 9-11)<br><br>12.2: Figure 1 – Cohort flow diagram (page 12)
Linkage		..		RECORD 12.3: State whether the study included person-level, institutional-level, or other data linkage across two or more databases. The methods of linkage and methods of linkage quality evaluation should be provided.	12.3: Methods - Study design (page 7-9)<br><br>The HAPPEN survey and linked SAIL data (page 9-11)
**Results**					
Participants	13	(a) Report the numbers of individuals at each stage of the study (*e.g.*, numbers potentially eligible, examined for eligibility, confirmed eligible, included in the study, completing follow-up, and analysed)<br>(b) Give reasons for non-participation at each stage.<br>(c) Consider use of a flow diagram		RECORD 13.1: Describe in detail the selection of the persons included in the study (*i.e.*, study population selection) including filtering based on data quality, data availability and linkage. The selection of included persons can be described in the text and/or by means of the study flow diagram.	13.1: Methods - Quantitative analysis (page 11-12)<br><br>Figure 1: Cohort flow diagram (page 12)
Descriptive data	14	(a) Give characteristics of study participants (*e.g.*, demographic, clinical, social) and information on exposures and potential confounders			Results - Table 1 Descriptive statistics (page 13-14)<br>Full descriptive statistics table: Online

For peer review only

Online supplemental appendix 2: RECORD statement

		(b) Indicate the number of participants with missing data for each variable of interest<br>(c) *Cohort study* - summarise follow-up time (*e.g.*, average and total amount)			supplemental appendix 5
Outcome data	15	*Cohort study* - Report numbers of outcome events or summary measures over time<br>*Case-control study* - Report numbers in each exposure category, or summary measures of exposure<br>*Cross-sectional study* - Report numbers of outcome events or summary measures			Results - Table 1 Descriptive statistics (page 13-14)<br>Full descriptive statistics table: Online supplemental appendix 6
Main results	16	(a) Give unadjusted estimates and, if applicable, confounder-adjusted estimates and their precision (e.g., 95% confidence interval). Make clear which confounders were adjusted for and why they were included<br>(b) Report category boundaries when continuous variables were categorized<br>(c) If relevant, consider translating estimates of relative risk into absolute risk for a meaningful time period			Results – Table 3 and table 4 (page 16-17)<br><br>Online supplemental appendix 6: Unadjusted multivariable logistic regression analyses
Other analyses	17	Report other analyses done—e.g., analyses of subgroups and interactions, and sensitivity analyses			Results (page 13-18)
**Discussion**					

For peer review only

Online supplemental appendix 2: RECORD statement

Key results	18	Summarise key results with reference to study objectives			Results (page 13-18)
Limitations	19	Discuss limitations of the study, taking into account sources of potential bias or imprecision. Discuss both direction and magnitude of any potential bias		RECORD 19.1: Discuss the implications of using data that were not created or collected to answer the specific research question(s). Include discussion of misclassification bias, unmeasured confounding, missing data, and changing eligibility over time, as they pertain to the study being reported.	Strengths and limitations (page 3-4)
Interpretation	20	Give a cautious overall interpretation of results considering objectives, limitations, multiplicity of analyses, results from similar studies, and other relevant evidence			Discussion (page 18-21), Conclusion (page 22-23)
Generalisability	21	Discuss the generalisability (external validity) of the study results			Discussion (page 18-21), Conclusion (page 21-22), Strengths and limitations (page 3-4)
**Other Information**					
Funding	22	Give the source of funding and the role of the funders for the present study and, if applicable, for the original study on which the present article is based			Funding (page 23)
Accessibility of protocol, raw data, and programming code		..		RECORD 22.1: Authors should provide information on how to access any supplemental information such as the study protocol, raw data, or programming code.	Availability for data and materials (page 22)

For peer review only

Online supplemental appendix 2: RECORD statement

*Reference: Benchimol EI, Smeeth L, Guttmann A, Harron K, Moher D, Petersen I, Sørensen HT, von Elm E, Langan SM, the RECORD Working Committee. The REporting of studies Conducted using Observational Routinely-collected health Data (RECORD) Statement. *PLoS Medicine* 2015; in press.

*Checklist is protected under Creative Commons Attribution (CC BY) license.

For peer review only

THE HAPPEN SURVEY

* Required

Consent Form

Before you start please click this link to read the information sheet -> https://happen-wales.co.uk/wp-content/uploads/2019/02/Child-Consent-2019.pdf

1. I have read the child information sheet -> https://happen-wales.co.uk/wp-content/uploads/2019/02/Child-Consent-2019.pdf (click the link if you haven't read it) and understand that if I take part I can change my mind at any time, and this will not be a problem at all. *

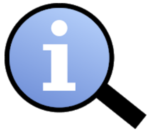

Mark only one oval.

- Yes
- No

2. I am happy for you to use my questionnaire for research. Only the researchers in the team will know my name and will not tell anyone else my answers *

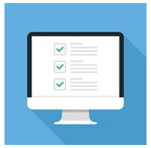

Mark only one oval.

- Yes
- No do not use my questionnaire

3. I am happy for you to look at my school and health records to see how my school is doing (as a group). This is anonymous which means I cannot be identified *

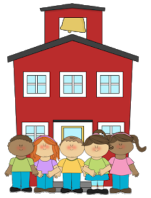

Mark only one oval.

- Yes
- No

If you do not wish to take part in the questionnaire please do not continue.

Please click next to start the questionnaire!

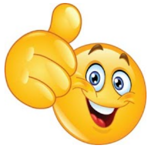

About You

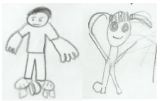

4. First Name *

5. Last Name *

6. Home Post Code *

7. What school do you go to? *

8. What year are you in? *

Mark only one oval.

- Year 4
- Year 5
- Year 6
- Year 7

9. Gender *

Mark only one oval.

- Boy
- Girl
- Prefer not to say

Date of Birth

10. Year *

Mark only one oval.

- 2007
- 2008
- 2009
- 2010
- 2011
- 2012

11. Month *

Mark only one oval.

- ( ) January
- ( ) February
- ( ) March
- ( ) April
- ( ) May
- ( ) June
- ( ) July
- ( ) August
- ( ) September
- ( ) October
- ( ) November
- ( ) December

12. Day *

Mark only one oval.

- ( ) 1
- ( ) 2
- ( ) 3
- ( ) 4
- ( ) 5
- ( ) 6
- ( ) 7
- ( ) 8
- ( ) 9
- ( ) 10
- ( ) 11
- ( ) 12
- ( ) 13
- ( ) 14
- ( ) 15
- ( ) 16
- ( ) 17
- ( ) 18
- ( ) 19
- ( ) 20
- ( ) 21
- ( ) 22
- ( ) 23
- ( ) 24
- ( ) 25
- ( ) 26
- ( ) 27
- ( ) 28
- ( ) 29
- ( ) 30
- ( ) 31

YESTERDAY

CHAT Child Health and Activity Tool

Firstly, think carefully about what you did YESTERDAY and then answer the following questions....

13. 1. What did you eat for breakfast YESTERDAY? *

Check all that apply.

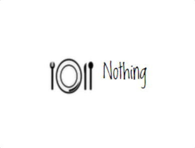

- [ ] Nothing

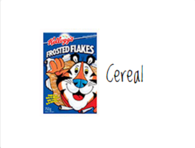

- [ ] Sugary cereal e.g. cocopops, frosties, sugar puffs, chocolate cereals

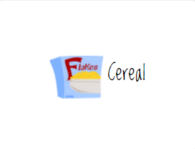

- [ ] Healthy cereal e.g. porridge, weetabix, readybrek, muesli, branflakes, cornflakes

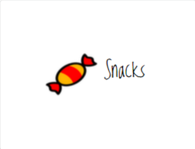

- [ ] Snacks

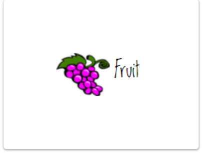

- [ ] Fruit

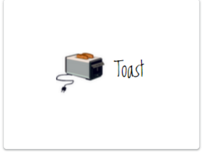

- [ ] Toast

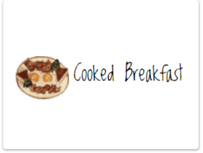

- [ ] Cooked breakfast

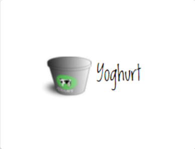

- [ ] Yoghurt

Other: [ ] ____________

14. 2. How did you get to school YESTERDAY morning? *

Mark only one oval.

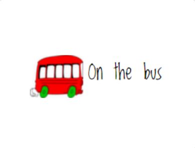

On the bus

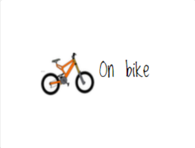

On bike

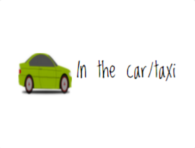

In the car/taxi

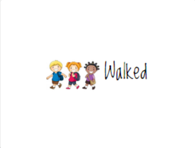

Walked

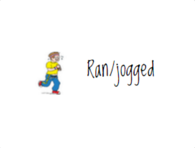

Ran/jogged

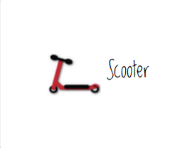

Scooter

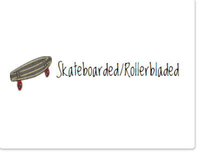

Skateboarded/Rollerbladed

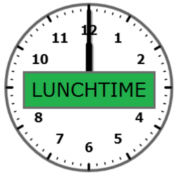

15. 3. What did you have to eat for lunch YESTERDAY? *

Mark only one oval.

School dinner
Packed lunch
Nothing

16. 4. What did you do for MOST of your break-times YESTERDAY? (This includes lunchtime) *

Mark only one oval.

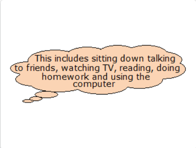

Sat around inside or outside

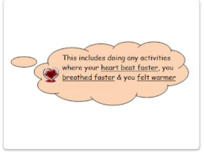

Ran around

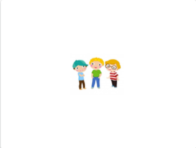

Stood around

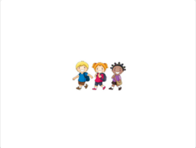

Walked around

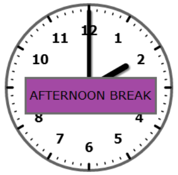

17. 5. Do you have an afternoon break at school? *

Mark only one oval.

YES
NO

For peer review only

18. 6. How did you get home YESTERDAY? *

Mark only one oval.

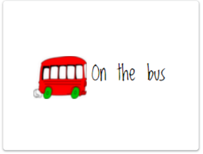

- On the bus

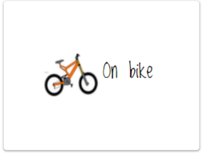

- On bike

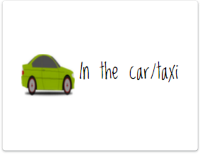

- In the car/taxi

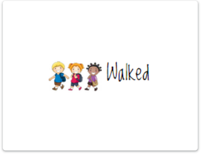

- Walked

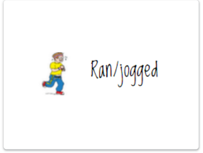

- Ran/jogged

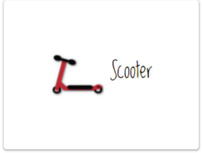

- Scooter

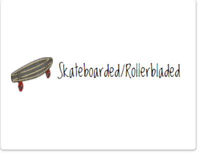

- Skateboarded/Rollerbladed

AFTER SCHOOL

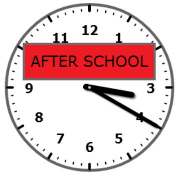

19. 7. How many portions of fruit and vegetables did you eat YESTERDAY? *

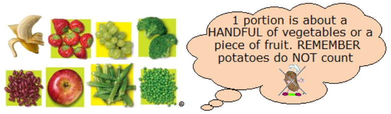

Mark only one oval.

- 0
- 1
- 2
- 3
- 4
- 5
- 6
- 7
- 8

20. 8. How many times did you brush your teeth YESTERDAY? *

Mark only one oval.

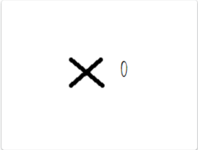

- 0

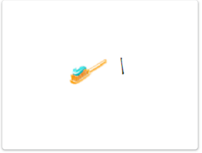

- 1

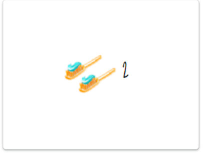

- 2

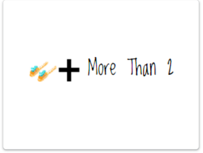

- 3

21. 9. What time did you fall asleep YESTERDAY (to the nearest half hour)? *

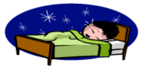

Mark only one oval.

- 7:00pm
- 7:30pm
- 8:00pm
- 8:30pm
- 9:00pm
- 9:30pm
- 10:00pm
- 10:30pm
- 11:00pm
- 11:30pm
- 12:00am
- 12:30am
- 1:00am
- 1:30am
- 2:00am
- 3:00am
- 3:30am
- 4:00am

22. 10. What time did you wake up TODAY (to the nearest half hour)? *

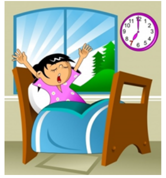

Mark only one oval.

- 5:00am
- 5:30am
- 6:00am
- 6:30am
- 7:00am
- 7:30am
- 8:00am
- 8:30am
- 9:00am

THE LAST WEEK

NOW think about what you did in the last 7 days...

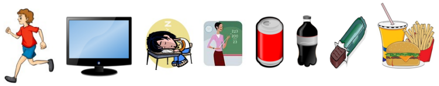

23. 11a. In the last 7 days, how many days did you do sports or exercise for at least 1 hour in total (This includes doing any activities or playing sports where your heart beat faster, you breathed faster and you felt warmer? *

Mark only one oval.

- 0 days
- 1-2 days
- 3-4 days
- 5-6 days
- 7 days

24. 11b. In the last 7 days, how many days did you watch TV/play online games/use the internet etc. for 2 or more hours a day (in total)? *

Mark only one oval.

- 0 days
- 1-2 days
- 3-4 days
- 5-6 days
- 7 days

25. 11c. In the last 7 days, how many days did you feel tired? *

Mark only one oval.

- 0 days
- 1-2 days
- 3-4 days
- 5-6 days
- 7 days

26. 11d. In the last 7 days, how many days did you feel like you could concentrate/pay attention well in class? *

Mark only one oval.

- 0 days
- 1-2 days
- 3-4 days
- 5-6 days
- 7 days

27. 11e. In the last 7 days, how many days did you drink at least one fizzy drink (e.g. coke, fanta, sprite) *

Mark only one oval.

- 0 days
- 1-2 days
- 3-4 days
- 5-6 days
- 7 days

28. 11f. In the last 7 days, how many days did you eat at least one sugary snack (e.g. chocolate bar, sweets) *

Mark only one oval.

- 0 days
- 1-2 days
- 3-4 days
- 5-6 days
- 7 days

29. 11g. In the last 7 days, how many days did you eat take away foods (e.g. McDonalds, KFC, chinese) *

Mark only one oval.

- 0 days
- 1-2 days
- 3-4 days
- 5-6 days
- 7 days

Sport and Activity

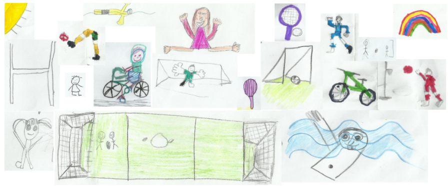

30. 12. These questions are going to ask you how you feel about physical activity (This includes any activity where your heart beats faster, you breathe faster and you feel warmer) *

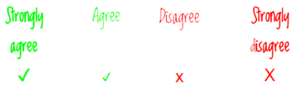

Mark only one oval per row.

	Strongly agree	Agree	Disagree	Strongly disagree
I want to take part in physical activity				
I feel confident to take part in lots of different physical activities				
I am good at lots of different physical activities				
I understand why taking part in physical activity is good for me				

31. 13a. How many times do you take part in a sports club OUTSIDE OF SCHOOL each week?

Mark only one oval.

0	1	2	3	4	5	6	7	8	9	10
◯	◯	◯	◯	◯	◯	◯	◯	◯	◯	◯

32. 13b. If you take part in a sports club OUTSIDE of school, what is the name of the sports club? (For example Swansea Rugby Club Under 11's)

33. 14. Are you a member of cubs, brownies, scouts or guides? *

Mark only one oval.

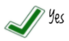

◯ Yes

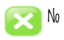

◯ No

34. 15. Which of these sports or physical activities would you MOST like to try? (That you haven't tried before) *

Mark only one oval.

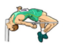

◯ Athletics

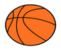

◯ Basketball

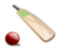

◯ Cricket

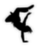

◯ Dance

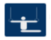

◯ Gymnastics

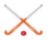

◯ Hockey

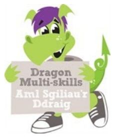

◯ Multi Skills

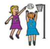

◯ Netball

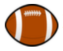

◯ Rugby

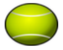

◯ Tennis

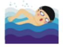

◯ Swimming

◯ I do not want to try anything-I don't like sport or activity

◯ Other: ____________

35. 16. Can you ride a bike WITHOUT STABILISERS? *

Mark only one oval.

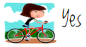

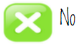

- Yes
- No

36. 17. Can you swim 25 metres WITHOUT A FLOAT OR ARMBANDS? (This is 1 length of a standard swimming pool) *

Mark only one oval.

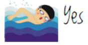

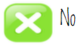

- Yes
- No

You and your feelings

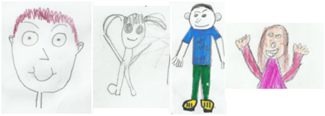

This part of the survey is going to ask you how you feel. There are no right or wrong answers. You should just pick the answer which is best for you.

37. 18. Tell us if you agree or disagree with the following: *

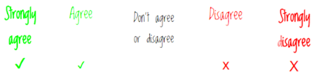

Mark only one oval per row.

	Strongly agree	Agree	Don't agree or disagree	Disagree	Strongly disagree
I am doing well at school					
I have lots of choice over things that are important to me					
There are lots of things I'm good at					

19. On a scale of 0 to 10 (0 being very unhappy and 10 being very happy, how do you feel about:

*Based on the Good Childhood Index by the Children's Society

38. Your Health *

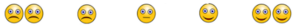

Mark only one oval.

	0	1	2	3	4	5	6	7	8	9	10	
Very unhappy												Very happy

39. Your School *

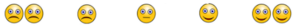

Mark only one oval.

	0	1	2	3	4	5	6	7	8	9	10	
Very unhappy												Very happy

40. Your Family *

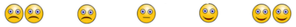

Mark only one oval.

	0	1	2	3	4	5	6	7	8	9	10	
Very unhappy												Very happy

41. Your Friends *

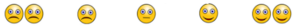

Mark only one oval.

	0	1	2	3	4	5	6	7	8	9	10	
Very unhappy												Very happy

42. Your Appearance (how you look) *

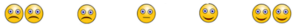

Mark only one oval.

	0	1	2	3	4	5	6	7	8	9	10	
Very unhappy												Very happy

43. Your Life *

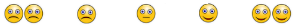

Mark only one oval.

	0	1	2	3	4	5	6	7	8	9	10	
Very unhappy												Very happy

You and your Feelings

Based on the Me and My Feelings Questionnaire (Deighton, Tymms, Vostanis, Belsky, Fonagy, Brown, Martin, Patalay, & Wolpert, 2012)

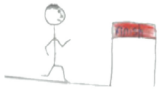

44. 20. Remember, there are no right or wrong answers, just pick which is right for you. *

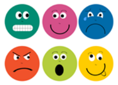

Mark only one oval per row.

	Never	Sometimes	Always
I feel lonely			
I cry a lot			
I am unhappy			
I feel nobody likes me			
I worry a lot			
I have problems sleeping			
I wake up in the night			
I am shy			
I feel scared			
I worry when I am at school			
I get very angry			
I lose my temper			
I hit out when I am angry			
I do things to hurt people			
I am calm			
I break things on purpose			

Your Local Area

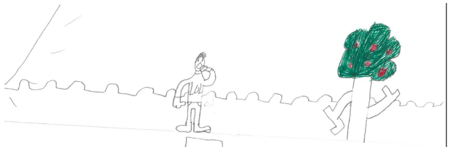

45. 21. On a scale of 0 to 10 (0 being not very safe and 10 being very safe), how safe do you feel playing in your area? *

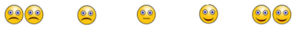

Mark only one oval.

	0	1	2	3	4	5	6	7	8	9	10	
Not very safe												Very safe

46. 22a. From your house, can you walk to school?

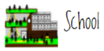

Mark only one oval.

- Yes
- No

47. 22b. From your house, can you easily walk to a park?

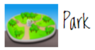

Mark only one oval.

- Yes
- No

48. 22c. From your house, can you easily walk to a leisure centre/sports centre?

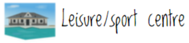

Mark only one oval.

- Yes
- No

49. 23. Are you happy with the area that you live in?

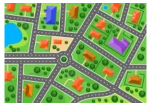

Mark only one oval.

- Yes
- No

24. If you could change something to make you and your friends healthier and happier, what would you change...

50. IN SCHOOL? *

51. OUT OF SCHOOL? *

Well done, you've completed the questionnaire.
Thank you!

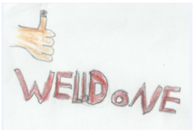

Don't forget to press submit below!

This content is neither created nor endorsed by Google.
Google Forms

For peer review only

Online supplemental appendix 4: HAPPEN survey variable codebook

Exposures	HAPPEN Survey item	Responses	Analyses coding
**Ate breakfast**	*13. What did you eat for breakfast yesterday?*	*Nothing*<br>*Cereal*<br>*Snacks*<br>*Fruit*<br>*Toast*<br>*Cooked breakfast*<br>*Yoghurt*	Binary:<br>*1 = Cereal; Snacks; Fruit; Toast; Cooked breakfast; Yoghurt*<br>*0 = Nothing*
**Active travel to school**	*14. How did you get to school yesterday morning?*	*On the bus*<br>*In the car/taxi*<br>*Walked*<br>*On bike*<br>*Ran/jogged*<br>*Scooter*<br>*Skateboarded/rollerbladed*	Binary:<br>*1 = Walked; On bike; Ran/jogged; Scooter; Skateboarded/rollerbladed*<br>*0 = On the bus; In the car/taxi*
**Active travel from school**	*18. How did you get home yesterday?*	*On the bus*<br>*In the car/taxi*<br>*Walked*<br>*On bike*<br>*Ran/jogged*<br>*Scooter*<br>*Skateboarded/rollerbladed*	Binary:<br>*1 = Walked; On bike; Ran/jogged; Scooter; Skateboarded/rollerbladed*<br>*0 = On the bus; In the car/taxi*
**Toothbrush 2+ per day**	*20. How many times did you brush your teeth yesterday?*	*0 – 3*	Continuous:<br>*0 – 3*
**5+ fruit and veg**	*19. How many portions of fruit and vegetables did you eat yesterday?*	*0 – 8*	Continuous:<br>*0 – 8*

For peer review only

Online supplemental appendix 4: HAPPEN survey variable codebook

**Sleep 9+ hours**	*21. What time did you fall asleep last night*	*(30 min intervals)*<br>*7:00pm – 4:00am*	Continuous:<br>*Sleep hours calculated from item 21 and 22*
	22. What time did you wake up this morning?	*(30 min intervals)*<br>*5:00am – 9:00am*	
**Physically active 60+ mins every day previous 7 days**	*23. In the last 7 days, how many days did you do sports or exercise for at least 1 hour in total (This includes doing any activities or playing sports where your heart beat faster, you breathed faster and you felt warmer*	*0 days*<br>*1 – 2 days*<br>*3 – 4 days*<br>*5 – 6 days*<br>*7 days*	Ordinal:<br>*0 days*<br>*1 – 2 days*<br>*3 – 4 days*<br>*5 – 6 days*<br>*7 days*
**Sedentary/screen time 2 hours every day previous 7 days**	*24. In the last 7 days, how many days did you watch TV/play online games/use the internet etc. for 2 or more hours a day (in total)?*	*0 days*<br>*1 – 2 days*<br>*3 – 4 days*<br>*5 – 6 days*<br>*7 days*	Ordinal:<br>*0 days*<br>*1 – 2 days*<br>*3 – 4 days*<br>*5 – 6 days*<br>*7 days*
**Tired 7 days**	*25. In the last 7 days, how many days did you feel tired?*	*0 days*<br>*1 – 2 days*<br>*3 – 4 days*<br>*5 – 6 days*<br>*7 days*	Ordinal:<br>*0 days*<br>*1 – 2 days*<br>*3 – 4 days*<br>*5 – 6 days*<br>*7 days*

For peer review only

Online supplemental appendix 4: HAPPEN survey variable codebook

**Sugary snack 7 days**	*28. In the last 7 days, how many days did you eat at least one sugary snack (e.g. chocolate bar, sweets)*	*0 days*<br>*1 – 2 days*<br>*3 – 4 days*<br>*5 – 6 days*<br>*7 days*	Ordinal:<br>*0 days*<br>*1 – 2 days*<br>*3 – 4 days*<br>*5 – 6 days*<br>*7 days*
Participate in at least 3 out of school clubs	*31. How many times do you take part in a sports club OUTSIDE OF SCHOOL each week?*	*0 - 10*	Continuous:<br>*0 - 10*
Can ride a bike	*35. Can you ride a bike without stabilisers?*	*No*<br>*Yes*	Binary:<br>*1 = Yes*<br>*0 = No*
Can swim 25m	*36. Can you swim 25 metres without a float or armbands (This is 1 length of a standard swimming pool)*	*No*<br>*Yes*	Binary:<br>*1 = Yes*<br>*0 = No*
Age on 01/03/2020	*Decimal age on 1 March 2020*	Continuous	Continuous
Sex	*Sex*	*Girl*<br>*Boy*	Binary:<br>*0 = Girl*<br>*1 = Boy*
WIMD	*Welsh Index of Multiple Deprivation 2019*		Coding framework from WIMD 2019[34]

For peer review only

Online supplemental appendix 5: Full descriptive statistics table by tested for SARS-CoV-2 and tested positive for SARS-CoV-2.

		Tested for SARS-CoV-2 n (%)	Not tested for SARS-CoV-2 n (%)	Tested positive for SARS-CoV-2 n (%)	No evidence of positive SARS-CoV-2 test n (%)
**Sample**		39.1% (2,764)	60.9% (4,298)	8.1% (569)	91.9% (6,498)
**Age at time of HAPPEN survey**		10.1 ± 0.8	9.9 ± 0.9	10.1 ± 0.8	9.9 ± 0.8
**Age on 01/03/2020 (start of period of interest)**		10.6 ± 0.9	10.3 ± 1.1	10.6 ± 1.0	10.4 ± 1.0
**Sex**	**Boy**	49.3% (1,363)	46.7% (2,005)	44.3% (252)	48.0% (3,116)
	**Girl**	48.9% (1,352)	51.8% (2,226)	54.5% (310)	50.3% (3,268)
	Missing	*1.8% (49)*	*1.5% (67)*	1.2% (7)	1.7% (109)
**WIMD 2019 quintiles**	**1 (most deprived)**	24.3% (672)	23.9% (1,025)	28.5% (162)	23.6% (1,535)
	**2**	19.9% (551)	19.02% (826)	19.7% (112)	19.5% (1,265)
	**3**	16.5% (455)	17.4% (748)	17.6% (100)	17.0% (1,103)
	**4**	15.6% (431)	15.8% (678)	14.1% (80)	15.9% (1,029)
	**5 (least deprived)**	18.0% (497)	16.8% (771)	16.5% (94)	17.3% (1,124)
	Missing	*5.7% (158)*	*7.0% (300)*	*3.7% (21)*	*6.7% (5437)*
		**Previous day**			
**Ate breakfast**	**Yes**	93.0% (2,571)	92.1% (3,797)	93.4% (538)	92% (6,012)
	**No**	7% (193)	7.3% (319)	5.6% (31)	7.3% (481)
	Missing	*0%*	*0%*	*0%*	*0%*
**Active travel to school**	**Yes**	38.5% (1,065)	39.8% (1,710)	37.6% (214)	39.4% (2,561)
	**No**	61.5% (1,699)	60.2% (2,588)	62.4% (355)	60.6% (3,932)

For peer review only

	Missing	*0%*	*0%*	*0%*	*0%*
**Active travel from school**	**Yes**	43.0% (1,187)	43.0% (1,846)	42.4% (241)	43.0% (2,792)
	**No**	57.0% (1,577)	57.0% (2,452)	57.6% (328)	57.0% (3,701)
	Missing	*0%*	*0%*	*0%*	*0%*
**Toothbrush continuous**	**0**	3.3% (91)	3.4% (146)	1.9% (11)	3.5% (227)
	**1**	20.0% (552)	21.0% (903)	18.6% (106)	20.6% (1,358)
	**2**	67.1% (1,854)	65.2% (2,802)	69.6% (396)	65.2% (4,294)
	**3**	9.6% (265)	10.3% (446)	9.5% (54)	10.0% (659)
	Missing	*0.1% (<5)*	*<0.1% (<5)*	*0.4% (<5)*	*<0.1%% (<5)*
**Fruit/veg portions (continuous)**	**0**	14.3% (395)	15.3% (657)	12.5% (71)	15.1% (981)
	**1**	16.1% (445)	17.4% (749)	15.8% (90)	17.0% (1,104)
	**2**	17.7% (489)	17.5% (754)	19.5% (111)	17.4% (1,132)
	**3**	17.5% (484)	16.5% (711)	16.7% (95)	16.9% (1,110)
	**4**	12.7% (351)	11.9% (510)	13.5% (77)	12.1% (784)
	**5**	10.5% (291)	10.6% (455)	11.8% (67)	10.4% (679)
	**6**	4.5% (123)	4.3% (186)	2.8% (16)	4.5% (293)
	**7**	2.3% (63)	2.1% (92)	4.2% (24)	2.0% (131)
	**8**	4.5% (123)	4.3% (184)	3.2% (18)	4.5% (289)
	Missing	0%	0%	*0%*	*0%*
**Sleep hours**		9.4 ± 1.6	9.4 ± 1.6	9.4 ± 1.6	9.4 ± 1.6
**Number of days physically active ≥ 60 minutes**	**0**	6.5% (179)	7.9% (339)	4.0% (23)	7.6% (495)
	**1-2 days**	27.9% (772)	29.0% (1,246)	27.8% (158)	28.7% (1,860)
	**3-4 days**	27.5% (761)	26.2% (1,128)	30.9% (176)	26.4% (1,712)
	**5-6 days**	18.3% (505)	17.0% (731)	18.1% (103)	17.5% (1,133)

For peer review only

	**7 days**	19.8% (557)	19.9% (854)	19.2% (109)	19.9% (1,292)
	Missing	*0%*	*0%*	*0%*	*0%*
**Number of days sedentary/screen time ≥ two hours**	**0**	5.2% (144)	6.1% (262)	5.5% (31)	5.8% (375)
	**1-2 days**	24.2% (674)	23.5% (1,011)	24.8% (141)	23.8% (1,544)
	**3-4 days**	21.7% (599)	20.6% (886)	21.1% (120)	21.0% (1,365)
	**5-6 days**	14.0% (386)	13.8% (593)	13.9% (79)	13.9% (900)
	**7 days**	34.8% (961)	36.0% (1,546)	34.8% (198)	35.6% (2,309)
	Missing	*0%*	*0%*	*0%*	*0%*
**Number of days tired**	**0**	21.0% (582)	21.0% (903)	19.2% (109)	21.2% (1,376)
	**1-2 days**	32.4% (895)	32.0% (1,377)	35.7% (203)	31.9% (2,069)
	**3-4 days**	17.6% (487)	17.5% (754)	18.8% (107)	17.5% (1,134)
	**5-6 days**	10.0% (276)	9.3% (399)	10.5% (60)	9.5% (615)
	**7 days**	19.0% (524)	20.1% (865)	15.8% (90)	20.0% (1,299)
	Missing	*0%*	*0%*	*0%*	*0%*
**Number of days sugary snack**	**0**	6.5% (179)	7.7% (332)	6.3% (36)	7.3% (475)
	**1-2 days**	34.9% (964)	32.7% (1,407)	35.0% (199)	33.5% (2,172)
	**3-4 days**	25.3% (698)	26.7% (1,146)	25.1% (143)	26.2% (1,701)
	**5-6 days**	13.4% (371)	12.0% (515)	15.3% (87)	12.3% (799)
	**7 days**	20.0% (552)	20.9% (898)	18.3% (104)	20.7% (1,346)
	Missing	*0%*	*0%*	*0%*	*0%*
			**General**		
**Number of out of school clubs**	**0**	27.7% (766)	32.3% (1,387)	25.1% (143)	31.0% (2,010)
	**1**	17.9% (495)	16.9% (726)	16.0% (91)	17.4% (1,130)
	**2**	16.0% (443)	15.1% (650)	14.9% (85)	15.5% (1,008)
	**3**	11.1% (308)	10.4% (446)	13.3% (76)	10.4% (678)

For peer review only

	**4**	7.4% (204)	7.3% (313)	7.6% (43)	7.3% (474)
	**5**	6.2% (171)	5.8% (251)	5.8% (33)	6.0% (389)
	**6**	3.4% (95)	2.5% (109)	5.1% (29)	2.7% (175)
	**7**	3.3% (91)	2.5% (107)	5.1% (29)	2.6% (169)
	**8**	1.1% (29)	0.8% (33)	1.8% (10)	0.8% (52)
	**9**	0.9% (24)	0.7% (32)	1.2% (7)	0.8% (49)
	**10**	3.9% (107)	4.0% (174)	3.3% (19)	4.0% (262)
	Missing	*1.1% (31)*	*1.6% (70)*	*0.7% (<5)*	*1.5% (97)*
**Can ride a bike**	**Yes**	88.8% (2,444)	86.0% (3,696)	91.4% (520)	86.7% (5,641)
	**No**	11.2% (309)	14.0% (602)	8.6% (49)	13.3% (862)
	Missing	*0%*	*0%*	*0%*	*0%*
**Can swim 25m**	**Yes**	78.9% (2,180)	72.9% (3,134)	80.3% (457)	74.8% (4,857)
	**No**	21.1% (584)	27.1% (1,164)	19.7% (112)	25.2% (1,636)
	Missing	*0%*	*0%*	*0%*	*0%*

For peer review only

Online supplemental appendix 6:
Multivariable logistic regression model of health behaviour markers and probability of PCR-test without confounders.

PCR tested for SARS-CoV-2 (n=6,958, R^2=0.01)	OR	*p* value	95% CI
**Ate breakfast**	1.05	0.632	0.87 – 1.27
Reference: did not eat breakfast	1.00		
**Active travel to school**	0.92	0.238	0.80 – 1.06
Reference: did not active travel to school	1.00		
**Active travel from school**	1.08	0.273	0.94 – 1.24
Reference: did not active travel from school	1.00		
**Number of fruit/vegetable portions**	1.00	0.941	0.98 – 1.03
Reference: 0 fruit/vegetable portions	1.00		
**Number of times teeth brushed**	0.97	0.474	0.90 – 1.05
Reference: did not brush teeth	1.00		
**Sleep hours**	0.99	0.654	0.96 – 1.02
Reference: 0 days physically active ≥60 mins (previous seven days)	1.00		
**1-2 days physically active ≥ 60 mins**	1.12	0.276	0.91 – 1.38
**3-4 days physically active ≥ 60 mins**	1.14	0.221	0.92 – 1.42
**5-6 days physically active ≥ 60 mins**	1.17	0.177	0.93 – 1.47
**7 days physically active ≥ 60 mins**	1.09	0.475	0.87 – 1.37
Reference: 0 days sedentary ≥two hours (previous seven days)	1.00		
**1-2 days sedentary ≥ two hours**	1.16	0.209	0.92 – 1.46
**3-4 days sedentary ≥ two hours**	1.18	0.166	0.93 – 1.49
**5-6 days sedentary ≥ two hours**	1.15	0.275	0.90 – 1.47
**7 days sedentary ≥ two hours**	1.14	0.256	0.91 – 1.44
Reference: 0 days felt tired (previous seven days)	1.00		
**1-2 days felt tired**	0.98	0.791	0.86 – 1.13
**3-4 days felt tired**	0.99	0.881	0.84 – 1.16
**5-6 days felt tired**	1.04	0.667	0.86 – 1.26
**7 days felt tired**	0.97	0.730	0.83 – 1.14
Reference: 0 days consumed sugary snack (previous seven days)			
**1-2 days consumed sugary snack**	1.21*	0.062	0.99 – 1.49
**3-4 days consumed sugary snack**	1.08	0.489	0.87 – 1.33
**5-6 days consumed sugary snack**	1.29**	0.034	1.02 – 1.63
**7 days consumed sugary snack**	1.12	0.314	0.90 – 1.39
**Number of out of school clubs participation**	1.02	0.121	1.00 to 1.04
**Can ride a bike**	1.16*	0.064	0.99 – 1.35

For peer review only

Reference: cannot ride a bike	1.00		
**Can swim 25m**	1.30**	0.000	1.15 – 1.46
Reference: cannot swim 25m	1.00		

OR: Odds Ratio; 95% CI: 95% confidence intervals; p<0.05**, p<0.1*. See online supplemental appendix 4 for variable codebook.

For peer review only

Multivariable logistic regression model of health behaviour markers and probability of PCR-test positive without confounders.

PCR test positive for SARS-CoV-2 (n=6,958, R^2=0.01)	OR	*p* value	95% CI
**Ate breakfast**	1.30	0.170	0.89 – 1.91
Reference: did not eat breakfast	1.00		
**Active travel to school**	0.91	0.451	0.71 – 1.17
Reference: did not active travel to school	1.00		
**Active travel from school**	1.07	0.614	0.83 – 1.36
Reference: did not active travel from school	1.00		
**Number of fruit/vegetable portions**	0.99	0.574	0.94 – 1.03
Reference: 0 fruit/vegetable portions	1.00		
**Number of times teeth brushed**	1.07	0.385	0.92 – 1.24
Reference: did not brush teeth	1.00		
**Sleep hours**	0.97	0.266	0.92 – 1.02
Reference: 0 days physically active ≥60 mins (previous seven days)	1.00		
**1-2 days physically active ≥ 60 mins**	1.71	0.023	1.08 – 2.73
**3-4 days physically active ≥ 60 mins**	1.87	0.009	1.17 – 2.99
**5-6 days physically active ≥ 60 mins**	1.61	0.059	0.98 – 2.63
**7 days physically active ≥ 60 mins**	1.49	0.117	0.91 – 2.43
Reference: 0 days sedentary ≥two hours (previous seven days)	1.00		
**1-2 days sedentary ≥ two hours**	1.03	0.877	0.68 – 1.57
**3-4 days sedentary ≥ two hours**	1.00	0.983	0.66 – 1.54
**5-6 days sedentary ≥ two hours**	1.01	0.958	0.65 – 1.59
**7 days sedentary ≥ two hours**	1.10	0.660	0.72 – 1.66
Reference: 0 days felt tired (previous seven days)	1.00		
**1-2 days felt tired**	1.21	0.125	0.95 – 1.55
**3-4 days felt tired**	1.17	0.278	0.88 – 1.55
**5-6 days felt tired**	1.21	0.273	0.86 – 1.69
**7 days felt tired**	0.92	0.600	0.69 – 1.24
Reference: 0 days consumed sugary snack (previous seven days)	1.00		
**1-2 days consumed sugary snack**	1.14	0.499	0.78 – 1.67
**3-4 days consumed sugary snack**	1.03	0.873	0.70 – 1.53
**5-6 days consumed sugary snack**	1.38	0.131	0.91 – 2.11
**7 days consumed sugary snack**	1.04	0.867	0.69 – 1.56
**Number of out of school clubs participation**	1.05	0.007	1.01 – 1.09
**Can ride a bike**	1.40	0.032	1.03 – 1.92

For peer review only

Reference: cannot ride a bike	1.00		
**Can swim 25m**	1.16	0.207	0.92 – 1.45
Reference: cannot swim 25m	1.00		

For peer review only

BMJ Open

Pre-COVID-19 pandemic health-related behaviours in children (2018-2020) and association with being tested for SARS-CoV-2 and testing positive for SARS-CoV-2 (2020-2021): a retrospective cohort study using survey data linked with routine health data in Wales, UK

Journal:	*BMJ Open*
Manuscript ID	bmjopen-2022-061344.R2
Article Type:	Original research
Date Submitted by the Author:	06-Jul-2022
Complete List of Authors:	Marchant, Emily ; Swansea University, Population Data Science, Medical School; Swansea University, Department of Education and Childhood Studies<br>Lowthian, Emily; Swansea University, Population Data Science, Medical School; Swansea University, Department of Education and Childhood Studies<br>Crick, Tom; Swansea University, Department of Education & Childhood Studies<br>Griffiths, Lucy ; Swansea University, Population Data Science, Medical School<br>Fry, Richard; Swansea University, Medical School; Swansea University, National Centre for Population Health and Wellbeing Research<br>Dadaczynski, Kevin; Fulda University of Applied Sciences, Department of Nursing and Health Science; Leuphana University Luneburg Center of Applied Health Sciences<br>Okan, Orkan; Technical University of Munich<br>James, M; Swansea University, Population Data Science, Medical School; Swansea University, National Centre for Population Health and Wellbeing Research<br>Cowley, Laura; Swansea University, Population Data Science, Medical School<br>Torabi, Fatemeh; Swansea University Medical School, Population Data Science<br>Kennedy, Jonathan; Swansea University, Population Data Science, Medical School; Swansea University, National Centre for Population Health and Wellbeing Research<br>Akbari, Ashley; Swansea University, Population Data Science, Medical School<br>Lyons, Ronan; Swansea University, Population Data Science, Medical School<br>Brophy, Sinead; Swansea University, Population Data Science, Medical School; Swansea University, National Centre for Population Health and Wellbeing Research
**Primary Subject Heading**:	Public health

Secondary Subject Heading:	Epidemiology, Infectious diseases, Paediatrics
Keywords:	Epidemiology < INFECTIOUS DISEASES, PUBLIC HEALTH, Public health < INFECTIOUS DISEASES, COVID-19, Community child health < PAEDIATRICS

SCHOLARONE™ Manuscripts

I, the Submitting Author has the right to grant and does grant on behalf of all authors of the Work (as defined in the below author licence), an exclusive licence and/or a non-exclusive licence for contributions from authors who are: i) UK Crown employees; ii) where BMJ has agreed a CC-BY licence shall apply, and/or iii) in accordance with the terms applicable for US Federal Government officers or employees acting as part of their official duties; on a worldwide, perpetual, irrevocable, royalty-free basis to BMJ Publishing Group Ltd ("BMJ") its licensees and where the relevant Journal is co-owned by BMJ to the co-owners of the Journal, to publish the Work in this journal and any other BMJ products and to exploit all rights, as set out in our licence.

The Submitting Author accepts and understands that any supply made under these terms is made by BMJ to the Submitting Author unless you are acting as an employee on behalf of your employer or a postgraduate student of an affiliated institution which is paying any applicable article publishing charge ("APC") for Open Access articles. Where the Submitting Author wishes to make the Work available on an Open Access basis (and intends to pay the relevant APC), the terms of reuse of such Open Access shall be governed by a Creative Commons licence – details of these licences and which Creative Commons licence will apply to this Work are set out in our licence referred to above.

Other than as permitted in any relevant BMJ Author's Self Archiving Policies, I confirm this Work has not been accepted for publication elsewhere, is not being considered for publication elsewhere and does not duplicate material already published. I confirm all authors consent to publication of this Work and authorise the granting of this licence.

For peer review only

Pre-COVID-19 pandemic health-related behaviours in children (2018-2020) and association with being tested for SARS-CoV-2 and testing positive for SARS-CoV-2 (2020-2021): a retrospective cohort study using survey data linked with routine health data in Wales, UK

Emily Marchant[1,2*], Emily Lowthian[1,2], Tom Crick[3], Lucy Griffiths[1], Richard Fry[1,3], Kevin Dadaczynski[4,5], Orkan Okan[6], Michaela James[1,3], Laura Cowley[3,7] Fatemeh Torabi[1], Jonathan Kennedy[1,3], Ashley Akbari[1], Ronan Lyons[1], Sinead Brophy[1,3]

[1] Population Data Science, Medical School, Swansea University, Swansea, SA2 8PP, United Kingdom

[2] Department of Education & Childhood Studies, Swansea University, Swansea, SA2 8PP, United Kingdom National Centre for Population Health and Wellbeing Research, Swansea University, Swansea, SA2 8PP, United Kingdom

[3] National Centre for Population Health and Wellbeing Research, Swansea University, Swansea, SA2 8PP, United Kingdom

[4] Department of Nursing and Health Science, Fulda University of Applied Sciences, Fulda, 36037, Germany

[5] Center for Applied Health Science, Leuphana University Lueneburg, Lueneburg, 21335, Germany

[6] Health Literacy, Department of Sport and Health Sciences, Technical University Munich, Uptown München-Campus D, 4. OG, OfficeL412, Georg-Brauchle-Ring 60/62, 80992 München, Germany

[7] Research and Evaluation Division, Public Health Knowledge and Research Directorate, Public Health Wales, 2 Capital Quarter, Cardiff, CF10 4BZ, United Kingdom

[*] Corresponding author: Emily Marchant [1,2], email: E.K.Marchant@swansea.ac.uk

For peer review only - http://bmjopen.bmj.com/site/about/guidelines.xhtml

ABSTRACT

Objectives

Examine if pre-COVID-19 pandemic (prior March 2020) health-related behaviours during primary school are associated with i) being tested for SARS-CoV-2 and ii) testing positive between 1 March 2020 to 31 August 2021.

Design

Retrospective cohort study using an online cohort survey (January 2018 to February 2020) linked to routine PCR SARS-CoV-2 test results.

Setting

Children attending primary schools in Wales (2018-2020), UK, who were part of the *HAPPEN* school network.

Participants

Complete linked records of eligible participants were obtained for n=7,062 individuals. 39.1% (n=2,764) were tested (age 10.6±0.9, 48.9% girls) and 8.1% (n=569) tested positive for SARS-CoV-2 (age 10.6±1.0, 54.5% girls).

For peer review only

Main outcome measures

Logistic regression of health-related behaviours and demographics were used to determine Odds Ratios (OR) of factors associated with i) being tested for SARS-CoV-2 and ii) testing positive for SARS-CoV-2.

Results

Consuming sugary snacks (1-2 days/week OR=1.24, 95% CI 1.04–1.49; 5-6 days/week 1.31, 1.07–1.61; reference 0 days) can swim 25m (1.21, 1.06–1.39) and age (1.25, 1.16–1.35) were associated with an increased likelihood of being tested for SARS-CoV-2. Eating breakfast (1.52, 1.01–2.27), weekly physical activity ≥ 60 mins (1-2 days 1.69, 1.04–2.74; 3-4 days 1.76, 1.10–2.82, reference 0 days), out of school club participation (1.06, 1.02–1.10), can ride a bike (1.39, 1.00–1.93), age (1.16, 1.05–1.28) and girls (1.21, 1.00–1.46) were associated with an increased likelihood of testing positive for SARS-CoV-2. Living in least deprived quintiles 4 (0.64, 0.46–0.90) and 5 (0.64, 0.46–0.89) compared to the most deprived quintile was associated with a decreased likelihood.

Conclusions

Associations may be related to parental health literacy and monitoring behaviours. Physically active behaviours may include co-participation with others, and exposure to SARS-CoV-2. A risk-versus-benefit approach must be considered in relation to promoting these health behaviours, given the importance of health-related behaviours such as childhood physical activity for development.

For peer review only

STRENGTHS AND LIMITATIONS

- Investigation of the association of pre-pandemic child health-related behaviour measures with subsequent SARS-CoV-2 testing and infection.
- Reporting of multiple child health behaviours linked at an individual-level to routine records of SARS-CoV-2 testing data through the SAIL Databank, using complete case analysis.
- Child-reported health behaviours were measured before the COVID-19 pandemic (1 January 2018 to 28 February 2020) which may not reflect behaviours during COVID-19.
- Health behaviours captured through the national-scale *HAPPEN* survey represent children attending schools that engaged with the *HAPPEN* Wales primary school network and may not be representative of the whole population of Wales.
- The period of study for PCR-testing includes a time frame with varying prevalence rates, approaches to testing children (targeted and mass testing) and restrictions which were not measured in this study.

For peer review only

For peer review only - http://bmjopen.bmj.com/site/about/guidelines.xhtml

BACKGROUND

The coronavirus disease 2019 (COVID-19) pandemic caused by severe acute respiratory syndrome coronavirus 2 (SARS-CoV-2) has resulted in widespread disruption to the lives of children across the world, and has contributed to widened inequalities in children's health, wellbeing and education[1,2]. Childhood is a critical developmental period during which health behaviours are established which transcend into adolescence and adulthood[3]. The Organisation for Economic Co-operation and Development (OECD) recognised current trends in children's health, highlighting typical health behaviours of school-aged children that warrant further research in order to better design policies that improve children's health outcomes[4,5]. These include nutrition-related behaviours such as fruit and vegetable intake, consumption of sugary foods and breakfast consumption, physical activity and sedentary behaviours and sleep. The establishment of these health behaviours during childhood are highly influenced by parental mechanisms and monitoring behaviours, particularly in children aged under 12[6–8].

Whilst evidence has demonstrated the negative impact of the COVID-19 pandemic on children's health-related behaviours including reduced physical activity, increased sedentary behaviour and poorer nutrition[1,9], it is unclear if this association is bidirectional. That is, whether these health behaviours are associated with likelihood of SARS-CoV-2 infection. Within the adult population, emerging evidence suggests a plausible relationship between pre-pandemic health risk behaviours such as physical inactivity and poor nutrition with SARS-CoV-2 infection and severity of disease[10–13], and increased risk of other infectious diseases[14]. This is attributed to the important role health behaviours play in shaping cardiometabolic health and immune system function. Indeed, research shows links to the

For peer review only

early years including critical early developmental stages with subsequent risk of developing chronic inflammation, which is associated with non-communicable disease risk and mortality during adulthood[15]. Health behaviours such as adequate nutrient intake[16] and physical activity[17] are required for the regulation and function of the immune system.

As a result, researchers have advocated for consideration to be placed on the role of these health behaviours in future endemic/pandemic scenarios[17]. However, research to date has concentrated on adults, explored single health behaviours or examined those with severe COVID-19 infection and hospitalisation[18,19]. The focus of research within the childhood population has principally been placed on clinical outcomes as opposed to lifestyle outcomes, including identifying the clinical characteristics of severe infection, the presence of comorbidities, common symptoms such as a cough and clinical biomarkers[20,21]. Whilst serious COVID-19 illness in children is relatively rare, mild or asymptomatic infection is common[22]. Positive SARS-CoV-2 tests require periods of self-isolation, impacting children's physical health and wellbeing, limiting opportunities for children to engage in health-promoting behaviours essential for optimal development such as regular physical activity[9,23]. Therefore, research examining the role of these health behaviours in a childhood population within the context of the COVID-19 pandemic is warranted.

Identifying the pre-pandemic health-related behavioural characteristics of children requiring a SARS-CoV-2 test or testing positive for SARS-CoV-2 infection and hypothesising potential mechanisms through which these may operate, including exposures, socio-demographic and parental influences could yield insight to inform the current COVID-19 pandemic and future pandemic/endemic scenarios. This can also allow targeted intervention to minimise transmission risk that complements national public health measures and

For peer review only - http://bmjopen.bmj.com/site/about/guidelines.xhtml

guidelines, and importantly, mitigates the disruption to children's lives and prevent further exacerbation of pre-existing inequalities, safeguarding their health, wellbeing and education.

In Wales (one of the four nations of the UK, with devolved health and social care policies), approaches to performing Polymerase Chain Reaction (PCR) tests on children during the period of study included the presence of COVID-19 symptoms, if identified as a close contact to a positive case (e.g. household contacts), or as a follow-up PCR test as encouraged in guidance at the time following a positive Lateral Flow Test (LFT) (e.g. showing symptoms or a close contact and having a positive LFT performed in the home)[24]. Uptake of testing within the childhood population requires parental monitoring behaviours; for example, providing transport to testing facilities and parental health literacy through identification of symptoms.

This study investigates the association of pre-pandemic (prior to 1 March 2020) health-related behaviours self-reported by children aged 8-11 years during primary school before the COVID-19 pandemic between 1 January 2018 and 28 February 2020, with two outcomes; the odds of ever having a SARS-CoV-2 PCR test and the odds of ever testing positive for SARS-CoV-2 during the period of study. We aim to examine whether these self-reported markers of health-related behaviours reported pre-pandemic are associated with the likelihood of; i) ever being tested for SARS-CoV-2 and ii) ever testing positive for SARS-CoV-2 between 1 March 2020 and 31 August 2021.

For peer review only - http://bmjopen.bmj.com/site/about/guidelines.xhtml

METHODS

Study design

This retrospective cohort study was conducted through the *HAPPEN* (Health and Attainment of Pupils in a Primary Education Network) primary school network[25]. HAPPEN was established in Wales, UK in 2014, following research with headteachers who advocated for increased collaboration to prioritise pupils' health and wellbeing[26,27], and is a platform for conducting school-based research[2,28–30]. The network brings together primary schools with research and runs up to the current date. School participation in *HAPPEN* is voluntary and is either once, annually or bi-annually (e.g. to evaluate school-based interventions). Through *HAPPEN*, children aged 8-11 (years 4 to 6) complete the *HAPPEN* survey, an online cohort survey that captures a range of validated self-reported health behaviours including physical activity, nutrition and sleep[31]. Retrospective health-related behaviour data were obtained from responses from the *HAPPEN* survey completed pre-pandemic between 1 January 2018 and 28 February 2020.

These retrospective survey responses were linked with PCR SARS-CoV-2 test results obtained from the Pathology COVID-19 Daily (PATD) routine dataset between 1 March 2020 and 31 August 2021. The PATD dataset contains pillar 1 (swab testing in Public Health England (PHE) labs, NHS Wales labs and NHS hospitals for those with a clinical need, and health and care workers) and pillar 2 (swab testing for the wider population, as set out in government guidance) individual results from PCR tests (negative (suspected), positive (confirmed) for SARS-CoV-2[32]. The period of interest (1 March 2020 to 31 August 2021) includes a time frame of varying approaches to testing children, documented in timeline format in online

For peer review only - http://bmjopen.bmj.com/site/about/guidelines.xhtml

supplemental appendix 1[32]. This includes targeted (i.e. symptomatic and suspected positive case, identified as a close contact of a positive case) and mass testing (i.e. between February 2021 and April 2021 the use of LFTs in the school setting for pupils aged 11 and above (secondary school age) to identify asymptomatic positive cases, with guidance for positive LFTs encouraging follow up PCR tests).

Linkage was performed using the SAIL (*Secure Anonymised Information Linkage*) Databank[33–35]. Data were linked at the individual level using an Anonymous Linkage Field (ALF) to identify participants and link SARS-CoV-2 test results (Figure 1). The RECORD checklist[36] for this study is presented in online supplemental appendix 2.

Ethics

Ethical approval was granted by the Swansea University Medical School Research Ethics Committee (2017-0033H). Electronic data (survey responses) were stored in secure files only accessible to the research team. The routine data used in this study are available in the SAIL Databank and are subject to review by an independent Information Governance Review Panel (IGRP), to ensure proper and appropriate use of SAIL data. Before any data can be accessed, approval must be received from the IGRP. When access has been approved, it is accessed through a privacy-protecting safe haven and remote access system referred to as the SAIL Gateway. SAIL has established an application process to be followed by anyone who would like to access data. This study has been approved by the SAIL IGRP (project reference: 0911).

For peer review only - http://bmjopen.bmj.com/site/about/guidelines.xhtml

The *HAPPEN* survey and linked SAIL data

All primary schools (n=1,203) in Wales, UK were invited to participate in the *HAPPEN* survey between 1 April 2014 and 28 February 2020 via a number of methods including email, social media promotion and through stakeholders in health and education (including local authority health and wellbeing teams, regional education consortia). Prior to 2018, *HAPPEN* was established in three of the local authorities (total n=22) in Wales. From 2018 to the period of interest, *HAPPEN* began its expansion to primary schools across Wales. Between 1 January 2018 and 28 February 2020, there were n=305 primary schools registered with *HAPPEN* (25% of primary schools in Wales). Participating in *HAPPEN* is voluntary and this study comprises of a convenience sample of children attending n=129 primary schools (representing a 42% response rate of registered *HAPPEN* primary schools) from 16 out of 22 local authorities that participated in the *HAPPEN* survey during the period of interest (1 January 2018 and 28 February 2020). Schools were invited to share details of the survey with parents/guardians (including information sheets). To participate in the *HAPPEN* survey and link data to routine records, child assent was required in addition to parental consent (between 2014 to 2018) and opt-out parental consent (2019 onwards).

The *HAPPEN* survey is completed by children aged 8-11 as a self-guided activity within the school setting as a classroom activity with supervision from a teacher/teaching assistant. The survey takes approximately 30 minutes to complete and includes validated self-report measures of typical health behaviours including physical activity, screen time, nutrition, sleep and wellbeing[31]. A full copy of the survey can be found in online supplemental appendix 3 and items, response categories and the coding framework included within analyses in online supplemental appendix 4.

For peer review only - http://bmjopen.bmj.com/site/about/guidelines.xhtml

The process of data coding involved two researchers. The first (MJ) cleaned the raw data (including checking for duplicate entries), removed identifiable information and generated a unique participant ID number to protect participants' anonymity. The second (EM) researcher coded the anonymised raw dataset using STATA (version 16) to produce a dataset for analyses. This *HAPPEN* dataset was uploaded to the SAIL Databank, a trusted research environment containing individual-level anonymised population-scale data sources about the population of Wales that enables secure data linkage and analysis for research, to be linked with SARS-CoV-2 testing data from the PATD dataset. To link the data, the person-based identifiable data are separated from the survey data and sent to a trusted third party, Digital Health and Care Wales (the national organisation that designs and builds digital services for health and social care in Wales). The survey data is sent to SAIL using a secure file upload. A unique Anonymous Linking Field (ALF) is assigned to the person-based record before it is joined to clinical data via a system linking field. The ALF was used to link records at the individual level between the *HAPPEN* dataset and PATD dataset containing PCR testing data. This dataset was accessible to authors listed from the Population Data Science group, Swansea University.

Quantitative analysis

The primary outcomes were i) whether the child was ever PCR-tested for the SARS-CoV-2 virus and ii) whether the child had any positive SARS-CoV-2 test between 1 March 2020 and 31 August 2021. Participants were assigned a binary code for any SARS-CoV-2 test during the period of interest (1: PCR-tested at least once for SARS-CoV-2 between 1 March 2020 and 31 August 2021; 0: no PCR SARS-CoV-2 test) and again for any positive SARS-CoV-2 test during

For peer review only - http://bmjopen.bmj.com/site/about/guidelines.xhtml

period of interest (1: any positive SARS-CoV-2 PCR test between 1 March 2020 and 31 August 2021; 0: negative PCR test for SARS-CoV-2; 0: not PCR-tested for SARS-CoV-2 (unknown)). In the case of multiple PCR tests, the first occurrence was used. Participants were assumed to have remained in Wales during the period of interest. Eligibility criteria (see cohort flow diagram, Figure 1) within final analyses models were any unique participant with complete linked survey and routine records. Inclusion dates of survey responses for analyses were between 1 January 2018 and 28 February 2020. Complete case multivariable logistic regression analyses, adjusting for confounding variables (sex, age on 1 March 2020, area-level deprivation using the Welsh Index of Multiple Deprivation (WIMD)[37] (version 2019) and clustered by school (using sandwich estimator to account for differences between schools), determined Odds Ratios (OR) for i) ever being PCR-tested for SARS-CoV-2 virus and ii) ever having a positive PCR SARS-CoV-2 test during the period of interest. Missing categories of data (sex and WIMD data obtained through the SAIL Databank) were tested to see if they significantly predicted any outcomes.

Independent variables as measures of typical pre-pandemic health-related behaviours included within analyses were obtained retrospectively from the *HAPPEN* survey, completed between 1 January 2018 and 28 February 2020 (online supplemental appendix 4). Health-related behaviour measures included in multivariable analyses are recognised by the OECD as typical health behaviour trends during childhood that warrant research[4,5]. These related to the behaviours from the previous day (ate breakfast, travel actively to and/or from school, number of fruit/vegetables portions consumed, number of times teeth brushed, hours of sleep), frequency of behaviours every day the previous seven days (physically active $\geq$ 60 minutes, sedentary/screen time $\geq$ two hours, felt tired, ate a sugary snack), and general items including participation in number of out of school clubs, can ride a bike and can swim 25

For peer review only - http://bmjopen.bmj.com/site/about/guidelines.xhtml

180 metres. A list of variables included in analyses, coding response categories and coding
181 framework is presented in online supplemental appendix 4. Independent variables were
182 entered concurrently and examined for association with the outcomes i) ever PCR-tested for
183 SARS-CoV-2 and ii) ever tested positive for SARS-CoV-2 between 1 March 2020 and 31 August
184 2021.

185

186 ***Figure 1: Cohort flow diagram***

187

188 Patient and public involvement

189 The SAIL Databank has a Consumer Panel that provides the public's perspective on
190 data linkage research. The Panel members are involved in all elements of the SAIL Databank
191 process, from developing ideas, advising on bids through approval processes (via the
192 independent Information Governance Review Panel), to disseminating research findings. For
193 more information visit https://saildatabank.com/about-us/public-engagement/.

194 RESULTS

195 Survey responses were obtained from n=11,339 participants (Figure 1). Survey
196 responses outside the period of interest (before 1 January 2018 and after 28 February 2020)
197 were excluded (n=3,698), followed by duplicate participants (occasions of multiple survey
198 entries, n=579). In the case of duplicates, the most recent instance of survey participation
199 was used. Complete linked unique records of participants meeting eligibility criteria were
200 obtained for n=7,062 individuals. Table 1 presents the descriptive statistics of the study

For peer review only - http://bmjopen.bmj.com/site/about/guidelines.xhtml

sample by ever PCR-tested for SARS-CoV-2 and ever tested positive for SARS-CoV-2 between 1 March 2020 and 31 August 2021. Of the total sample, 39.1% (n=2,764) were PCR-tested for SARS-CoV-2 and 8.1% (n=569) tested positive for SARS-CoV-2. The mean age on 1 March 2020 (start of period of interest) was 10.6 (± 0.9) for those PCR-tested (Table 1) and 10.6 (± 1.0) for those tested positive for SARS-CoV-2 (Table 2). The time between the *HAPPEN* survey date and SARS-CoV-2 PCR test date (median number of days (interquartile range)) was 588 (385 – 685) days for being PCR-tested and 672 (599 – 715) days for PCR testing positive for SARS-CoV-2. Complete case analyses are presented. The maximum missing data was 7% (see Table 1). We tested if missing categories of data (sex and WIMD obtained through the SAIL Databank) significantly predicted any outcomes and found that no missing categories significantly predicted the outcomes. Therefore, missing data were assumed to be at random through data linkage[38]. Unadjusted multivariable logistic regression analyses are presented in online supplemental appendix 5.

Table 1: Descriptive statistics of study sample by PCR-tested for SARS-CoV-2 and PCR test positive for SARS-CoV-2 between 1 March 2020 and 31 August 2021.

	**Tested for SARS-CoV-2 % (n)**	**Not tested for SARS-CoV-2 % (n)**	**Tested positive for SARS-CoV-2 % (n)**	**Tested negative/not tested (unknown) for SARS-CoV-2 % (n)**
**Sample**	39.1% (2,764)	60.9% (4,298)	8.1% (569)	91.9% (6,493)
**Age at time of *HAPPEN* survey**	10.1 ± 0.8	9.9 ± 0.9	10.1 ± 0.8	9.9 ± 0.8
**Age on 01/03/2020 (start of period of interest)**	10.6 ± 0.9	10.3 ± 1.1	10.6 ± 1.0	10.4 ± 1.0
**Number of days between *HAPPEN***	588 (385 – 685)		672 (599 – 715)	

For peer review only

For peer review only - http://bmjopen.bmj.com/site/about/guidelines.xhtml

**survey and SARS-CoV-2 test (median (IQR))**					
**Sex**	**Boy**	49.3% (1,363)	46.7% (2,005)	44.3% (252)	48.0% (3,116)
	**Girl**	48.9% (1,352)	51.8% (2,226)	54.5% (310)	50.3% (3,268)
	Missing	*1.8% (49)*	*1.5% (67)*	1.2% (7)	1.7% (109)
**WIMD 2019 quintiles**	**1 (most deprived)**	24.3% (672)	23.9% (1,025)	28.5% (162)	23.6% (1,535)
	**2**	19.9% (551)	19.02% (826)	19.7% (112)	19.5% (1,265)
	**3**	16.5% (455)	17.4% (748)	17.6% (100)	17.0% (1,103)
	**4**	15.6% (431)	15.8% (678)	14.1% (80)	15.9% (1,029)
	**5 (least deprived)**	18.0% (497)	16.8% (771)	16.5% (94)	17.3% (1,124)
	Missing	*5.7% (158)*	*7.0% (300)*	*3.7% (21)*	*6.7% (437)*

See online supplemental appendix 4 for variable codebook. Full descriptive statistics table presented in online supplemental appendix 5. IQR (interquartile range).

Table 2 presents the multivariable logistic regression for children ever PCR-tested for SARS-CoV-2 between 1 March 2020 and 31 August 2021. The model showed a low goodness-of-fit (R^2=0.02). Children that reported to eat breakfast (OR=1.16, 95% CI 0.99 – 1.36, reference: did not eat breakfast, p<0.1), consume sugary snacks on 1-2 days (1.24, 1.04 – 1.49) and 5-6 days (1.31, 1.07 – 1.61) compared to 0 days, participate in more out of school clubs (1.02, 1.00 – 1.04), able to ride a bike (1.15, 0.98 – 11.35, reference: cannot ride a bike, p<0.1) and able to swim 25m (1.21, 1.06 – 1.39, reference: cannot swim 25m) were more likely to be PCR-tested for SARS-CoV-2. Older children (1.25, 1.16 – 1.35) were also more likely to be PCR-tested for SARS-CoV-2, and compared to quintile 1 (most deprived) those in WIMD quintiles 3 (0.85, 0.70 – 1.03, p<0.1) and 5 (0.85, 0.72 – 1.02, p<0.1) were less likely to be PCR-tested for SARS-CoV-2. Unadjusted multivariable logistic regression analyses are presented in online supplemental appendix 6.

Table 2: Multivariable logistic regression model of significant health behaviour markers and probability of ever being PCR-tested for SARS-CoV-2 between 1 March 2020 and 31 August 2021, accounting for baseline age, sex and deprivation, and clustered by school.

For peer review only - http://bmjopen.bmj.com/site/about/guidelines.xhtml

PCR-tested for SARS-CoV-2 (n=6,403, R^2=0.02)	OR	*p* value	95% CI
**Ate breakfast**	1.16*	0.067	0.99 – 1.36
Reference: did not eat breakfast	1.00		
**Active travel to school**	0.93	0.339	0.80 – 1.08
Reference: did not active travel to school	1.00		
**Active travel from school**	1.01	0.901	0.86 – 1.19
Reference: did not active travel from school	1.00		
**Number of fruit/vegetable portions**	1.00	0.959	0.97 – 1.03
**Number of times teeth brushed**	0.94	0.229	0.86 – 1.04
**Sleep hours**	1.01	0.682	0.97 – 1.04
Reference: 0 days physically active ≥ 60 mins (previous seven days)			
**1-2 days physically active ≥ 60 mins**	1.14	0.250	0.91 – 1.41
**3-4 days physically active ≥ 60 mins**	1.13	0.257	0.91 – 1.39
**5-6 days physically active ≥ 60 mins**	1.16	0.217	0.92 – 1.45
**7 days physically active ≥ 60 mins**	1.10	0.451	0.86 – 1.39
Reference: 0 days sedentary ≥ two hours (previous seven days)	1.00		
**1-2 days sedentary ≥ two hours**	1.20	0.141	0.94 – 1.54
**3-4 days sedentary ≥ two hours**	1.18	0.198	0.92 – 1.52
**5-6 days sedentary ≥ two hours**	1.16	0.333	0.86 – 1.56
**7 days sedentary ≥ two hours**	1.16	0.243	0.90 – 1.48
Reference: 0 days felt tired (previous seven days)	1.00		
**1-2 days felt tired**	0.97	0.686	0.85 – 1.12
**3-4 days felt tired**	1.00	0.963	0.85 – 1.16
**5-6 days felt tired**	1.07	0.528	0.86 – 1.33
**7 days felt tired**	0.97	0.728	0.93 – 1.14
Reference: 0 days consumed sugary snack (previous seven days)	1.00		
**1-2 days consumed sugary snack**	1.24**	0.018	1.04 – 1.49
**3-4 days consumed sugary snack**	1.12	0.301	0.91 – 1.37
**5-6 days consumed sugary snack**	1.31**	0.008	1.07 – 1.61
**7 days consumed sugary snack**	1.16	0.170	0.94 – 1.43
**Number of out of school clubs participation**	1.02*	0.099	1.00 – 1.04
**Can ride a bike**	1.15*	0.086	0.98 – 1.35
Reference: cannot ride a bike	1.00		
**Can swim 25m**	1.21**	0.006	1.06 – 1.39
Reference: cannot swim 25m	1.00		
**Age 01/03/2020**	1.25**	< 0.001	1.16 – 1.35
**Sex (girl)**	0.92	0.161	0.81 – 1.04
Reference: sex (boy)	1.00		

For peer review only

Reference: WIMD 2019 quintile 1 (most deprived)	1.00		
**WIMD 2019 quintile 2**	0.95	0.600	0.80 – 1.14
**WIMD 2019 quintile 3**	0.85*	0.090	0.70 – 1.03
**WIMD 2019 quintile 4**	0.87	0.131	0.73 – 1.04
**WIMD 2019 quintile 5 (least deprived)**	0.85*	0.078	0.72 – 1.02

OR: Odds Ratio; 95% CI: 95% confidence intervals; **p <0.05, *p<0.1. See online supplemental appendix 4 for variable codebook. Low to moderate correlation between variables (coefficients -0.19 to 0.71). Complete case analysis.

Table 3 presents the multivariable logistic regression for children ever PCR-tested positive for SARS-CoV-2 between 1 March 2020 and 31 August 2021. Children were more likely to test positive for SARS-CoV-2 if reporting to eat breakfast (OR=1.52, 95% CI 1.01 – 2.27, reference: did not eat breakfast), be physically active for ≥ 60 mins on 1-2 days (1.69, 1.04 – 2.74), 3-4 days (1.76, 1.10 – 2.82) and 5-6 days (1.59, 0.93 – 2.73, p<0.1) compared to 0 days, participate in more out of school clubs (1.06, 1.02 – 1.10) and able to ride a bike (1.39, 1.00 – 1.93, reference: cannot ride a bike). Older children (1.16, 1.05 – 1.28) were more likely to test positive for SARS-CoV-2. Compared to boys, girls were more likely to test positive (1.21, 1.00 – 1.46), and compared to the most deprived quintile 1, those living in the least deprived quintiles 4 (0.64, 0.46 – 0.90) and 5 (0.64, 0.46 – 0.89) were less likely to test positive for SARS-CoV-2. The model showed a low goodness-of-fit (R^2=0.02). Unadjusted multivariable logistic regression analyses are presented in online supplemental appendix 6.

For peer review only

Table 3: Multivariable logistic regression model of significant health behaviour markers and probability of ever PCR-testing positive for SARS-CoV-2 between 1 March 2020 and 31 August 2021, accounting for baseline age, sex and deprivation, and clustered by school.

**PCR test positive for SARS-CoV-2 (n=6,403, R^2=0.02)**	**OR**	***p* value**	**95% CI**
**Ate breakfast**	1.52**	0.043	1.01 – 2.27
Reference: did not eat breakfast	1.00		
**Active travel to school**	0.91	0.481	0.70 – 1.18
Reference: did not active travel to school	1.00		
**Active travel from school**	0.98	0.910	0.72 – 1.33
Reference: did not active travel from school	1.00		

**Number of fruit/vegetable portions**	0.98	0.461	0.94 – 1.03
**Number of times teeth brushed**	1.05	0.542	0.90 – 1.21
**Sleep hours**	0.97	0.345	0.92 – 1.03
Reference: 0 days physically active ≥ 60 mins (previous seven days)	1.00		
**1-2 days physically active ≥ 60 mins**	1.69**	0.035	1.04 – 2.74
**3-4 days physically active ≥ 60 mins**	1.76**	0.018	1.10 – 2.82
**5-6 days physically active ≥ 60 mins**	1.59*	0.091	0.93 – 2.73
**7 days physically active ≥ 60 mins**	1.50	0.158	0.85 – 2.65
Reference: 0 days sedentary ≥ two hours (previous seven days)	1.00		
**1-2 days sedentary ≥ two hours**	0.96	0.847	0.63 – 1.47
**3-4 days sedentary ≥ two hours**	0.94	0.789	0.59 – 1.50
**5-6 days sedentary ≥ two hours**	0.93	0.803	0.51 – 1.68
**7 days sedentary ≥ two hours**	1.02	0.946	0.63 – 1.65
Reference: 0 days felt tired (previous seven days)	1.00		
**1-2 days felt tired**	1.17	0.207	0.91 – 1.51
**3-4 days felt tired**	1.17	0.232	0.91 – 1.50
**5-6 days felt tired**	1.19	0.243	0.89 – 1.60
**7 days felt tired**	0.89	0.390	0.68 – 1.16
Reference: 0 days consumed sugary snack (previous seven days)	1.00		
**1-2 days consumed sugary snack**	1.13	0.523	0.77 – 1.65
**3-4 days consumed sugary snack**	1.06	0.783	0.70 – 1.61
**5-6 days consumed sugary snack**	1.36	0.159	0.89 – 2.08
**7 days consumed sugary snack**	1.08	0.727	0.71 – 1.63
**Number of out of school clubs participation**	1.06**	0.002	1.02 – 1.10
**Can ride a bike**	1.39**	0.049	1.00 – 1.93
Reference: cannot ride a bike	1.00		
**Can swim 25m**	1.14	0.324	0.88 – 1.48
Reference: cannot swim 25m			
**Age 01/03/2020**	1.16**	0.003	1.05 – 1.28
**Sex (girl)**	1.21**	0.046	1.00 – 1.46
Reference: sex (boy)	1.00		
Reference: WIMD 2019 quintile 1 (most deprived)	1.00		
**WIMD 2019 quintile 2**	0.79	0.113	0.59 – 1.06
**WIMD 2019 quintile 3**	0.79	0.128	0.59 – 1.07
**WIMD 2019 quintile 4**	0.64**	0.009	0.46 – 0.90
**WIMD 2019 quintile 5**	0.64**	0.007	0.46 – 0.89

For peer review only - http://bmjopen.bmj.com/site/about/guidelines.xhtml

OR: Odds Ratio; 95% CI: 95% confidence intervals; **p<0.05, *p<0.1. See online supplemental appendix 4 for variable codebook. Low to moderate correlation between variables (coefficients -0.19 to 0.71). Complete case analysis.

DISCUSSION

This study examines whether markers of health-related behaviours reported by primary school-aged children between January 2018 and February 2020 are associated with the likelihood of ever being PCR-tested for SARS-CoV-2 and ever testing positive between 1 March 2020 and 31 August 2021. Findings suggest that reporting to eat breakfast, weekly sugary snack consumption (both low and high), participating in more out of school clubs, being able to ride a bike and being able to swim 25 metres were associated with an increased likelihood of being tested for SARS-CoV-2. Health behaviours associated with an increased likelihood of testing positive for SARS-CoV-2 were eating breakfast, engaging in higher weekly physical activity, participating in more out of school clubs and riding a bike. Boys were more likely to test positive for SARS-CoV-2 than girls, and those living in a less deprived area less likely to test positive that those residing in the most deprived area.

This study encompasses a period of both targeted and mass PCR testing, and detecting child positive cases using routine PCR testing data in this study requires a parent/guardian to take the child for testing. We find associations between child-reported health-related behaviours with both PCR-testing for SARS-CoV-2 and testing positive for SARS-CoV-2. Through this, we theorise that because health behaviours are largely guided and facilitated by parents, our associations are likely to be reflecting health literacy among parents, along with monitoring behaviours. In the case of symptomatic testing, the detection of positive child cases relies on parents recognising symptoms and communication with their child. For

For peer review only

For peer review only - http://bmjopen.bmj.com/site/about/guidelines.xhtml

asymptomatic testing through the use of LFT (e.g. asymptomatic school testing between February and April 2021), guidance encouraged positive LFTs to be followed up with PCR testing, requiring knowledge of how to access testing services and ability to access services (e.g. transport). These behaviours form a level of health literacy, recognised as the ability to access, understand, interpret and apply medical information and make informed decisions regarding medical advice, issues or guidelines[39]. Parental health literacy impacts the decision a parent makes relating to their child[40] and is correlated with a number of health indicators including knowledge of health and health services, and the parent and child engaging in health-promoting behaviours[8,39].

Parenting is an important contributor to promoting positive health behaviours in children, and is represented by a constellation of attitudes, behaviours and values for the child. The presence of multiple physically active behaviours represented by the association of being able to swim, ride a bike and participation in more out of school clubs may represent underlying parental involvement and modelling behaviour, including involvement in leisure-time activities, providing financial and transport provision to attend organised activities such as access to swimming lessons and the provision of equipment[7]. This may also have a socioeconomic component, building on the ideas of Bourdieu in terms of social capital, and accessing health-enhancing material items[41].

Diet-related findings of eating breakfast and restrictive weekly sugary snack consumption (1-2 days per week) may indicate higher parental monitoring, supporting our theory. In comparison, higher weekly sugary snack consumption (5-6 days per week) may represent less restrictive parental monitoring and more autonomy and choice for the child. We posit that as parental behaviours are often driven by underlying styles of parenting[42], the associations could be depicting varying levels of control; for instance, those snacking 1-2

For peer review only - http://bmjopen.bmj.com/site/about/guidelines.xhtml

times perhaps have parents with greater control, versus those snacking 5-6 times with parents with less controlling styles. This theory may well transcend into other behaviours, including limits and freedom in socialising with others, placing a greater likelihood of infection of illness – including COVID-19.

While evidence recognises the importance of adequate nutrition[16] and physical activity[17] for cardiometabolic health and immune system function, the findings in the current study draw attention to another potential mechanism of increased contacts and exposure to SARS-CoV-2. Engagement in physically active behaviours such as out of school clubs, higher frequency of physically active days in a week and riding a bike may increase the number of social contacts of the child. Indeed, there is a wealth of evidence demonstrating that childhood physical activity participation is highly influenced by their social environment and co-participation with peers[43]. It is therefore possible that physically active children had increased social contacts and exposure to SARS-CoV-2 through co-participation of activity and play opportunities.

However, it is important to note that physical activity is an essential health behaviour required for optimal development and a range of health and wellbeing outcomes. These findings must be considered in balance with the importance of encouraging these behaviours and providing physically active opportunities during childhood. This viewpoint was also reflected in Government guidance and risk assessments during the COVID-19 pandemic through the reopening of children's playgrounds and outdoor play spaces, with explicit reference to outdoor play and physical activity as fundamental for children's development and wellbeing[44].

Contact patterns may also explain sex differences observed in this study, as we found girls are more likely to test positive for SARS-CoV-2. In addition to age assortative mixing

For peer review only

For peer review only - http://bmjopen.bmj.com/site/about/guidelines.xhtml

patterns of children, there is a developmental tendency by children to socially interact with members of the same sex and engage in gender-typed activity[45]. For girls, the location of play preferences are more likely to be indoors and in contact with supervising adults, where exposure to SARS-CoV-2 is possibly greater[46]. The findings of association between increasing age and likelihood of testing positive for SARS-CoV-2 in this study are supported by wider literature which suggests increasing susceptibility of infection in the adolescent age group compared to younger than 10 to 14 years[47].

Our findings also show an area-level social gradient. Those living in the least deprived WIMD quintiles 4 and 5 were less likely to test positive for SARS-CoV-2 compared to the most deprived quintile, which may reflect deprivation-related exposure patterns to SARS-CoV-2. Indeed, research conducted using the WIMD and English area-level deprivation indictors found adults living in the most deprived areas demonstrated differential exposures to SARS-CoV-2[48]. This included patterns of public activities such as attending work or education outside of the household, using public transport and car sharing with non-household members. This, and considerations of the deprivation-related disparities in the built environment including access to open spaces highlight the inequalities that persist in SARS-CoV-2 infection. Furthermore, whilst it is likely that children mix with others from similar demographic areas, the finding in our study may also reflect community prevalence which was not captured.

CONCLUSION

We theorise that health-promoting behaviours associated with a child being tested for SARS-CoV-2 and being identified as positive may be a proxy of higher parental health

For peer review only

literacy and monitoring behaviours. Furthermore, co-participation in physically active behaviours with peers may increase exposure to SARS-CoV-2. This must be considered from a risk-versus-benefit approach in relation to promoting these health behaviours, given the importance of health-related behaviours such as physical activity during childhood for development and wellbeing. This national-level case study using survey data linked with routine health data in Wales provide insight into these issues from a devolved policymaking context, with the potential for replicability and portability to other jurisdictions.

Acknowledgements

The authors would like to thank all participating primary schools and pupils that took part in this study. This work was supported by the National Centre for Population Health and Wellbeing Research through the *HAPPEN* network. This study makes use of anonymised data held in the Secure Anonymised Information Linkage (SAIL) Databank. We would like to acknowledge all the data providers who make anonymised data available for research. We would also like to thank Dr Annemarie Docherty and Dr Olivia Swann from The University of Edinburgh for providing informal peer review input to the final draft.

Availability for data and materials

The routine data used in this study are available in the SAIL Databank at Swansea University, Swansea, UK. All proposals to use SAIL data are subject to review by an IGRP. Before any data can be accessed, approval must be given by the IGRP. The IGRP gives careful consideration to

For peer review only

For peer review only - http://bmjopen.bmj.com/site/about/guidelines.xhtml

each project to ensure proper and appropriate use of SAIL data. When access has been approved, it is gained through a privacy-protecting safe haven and remote access system referred to as the SAIL Gateway. SAIL has established an application process to be followed by anyone who would like to access data via SAIL https://www.saildatabank.com/application-process. This study has been approved by the IGRP as project 0911.

Funding

The Economic and Social Research Council (ESRC) funded the development of the *HAPPEN* network (grant number: ES/J500197/1) which this research was conducted through. The National Centre for Population Health and Wellbeing Research (NCPHWR) funded by Health and Care Research Wales provided infrastructural support for this work. This work was supported by the Con-COV team funded by the Medical Research Council (grant number: MR/V028367/1). This work was supported by Health Data Research UK, which receives its funding from HDR UK Ltd (HDR-9006) funded by the UK Medical Research Council, Engineering and Physical Sciences Research Council, Economic and Social Research Council, Department of Health and Social Care (England), Chief Scientist Office of the Scottish Government Health and Social Care Directorates, Health and Social Care Research and Development Division (Welsh Government), Public Health Agency (Northern Ireland), British Heart Foundation (BHF) and the Wellcome Trust. This work was a collaboration with the ADR Wales programme of work. ADR Wales is part of the Economic and Social Research Council (part of UK Research and Innovation) funded ADR UK (grant ES/S007393/1). This work was supported by the Wales COVID-19 Evidence Centre, funded by Health and Care Research

For peer review only

Wales and by the COVID-19 Longitudinal Health and Wellbeing National Core Study funded by the Medical Research Council (MC_PC_20030).

Transparency statement

The lead author affirms that the manuscript is an honest, accurate, and transparent account of the study being reported; that no important aspects of the study have been omitted; and that any discrepancies from the study as originally planned have been explained.

Competing interests

The authors declare that they have no competing interests.

Contributorship statement

EM and SB conceptualised the study design. EM and MJ acquired the data, and EM and JK were responsible for data curation. EM performed the statistical analysis, undertook the initial interpretation of the data and wrote the initial draft. EL and SB contributed to the writing of the manuscript and provided statistical guidance. EL, JK, SB, LC and RL provided critical interpretation of the data. The manuscript was critically reviewed and edited by EL, TC, LG, RF, KD, OO, MJ, LC, FT, JK, AA, RL and SB. SB provided supervision and TC and LG provided mentorship. EM is the guarantor. AD and OS critically reviewed the initial manuscript. EM, EL, TC, LG, RF, KD, OO, MJ, LC, FT, JK, AA, RL and SB approved the final manuscript and agreed to be accountable for all aspects of the work.

For peer review only

References

1 James M, Marchant E, Defeyter MA, *et al.* Impact of school closures on the health and well-being of primary school children in Wales UK: a routine data linkage study using the HAPPEN Survey (2018-2020). *BMJ Open* 2021;**11**. doi:10.1136/BMJOPEN-2021-051574

2 Marchant E, Todd C, James M, *et al.* Primary school staff perspectives of school closures due to COVID-19, experiences of schools reopening and recommendations for the future: A qualitative survey in Wales. *PLoS One* 2021;**16**:e0260396. doi:10.1371/JOURNAL.PONE.0260396

3 Craigie AM, Lake AA, Kelly SA, *et al.* Tracking of obesity-related behaviours from childhood to adulthood: A systematic review. *Maturitas* 2011;**70**:266–84. doi:10.1016/j.maturitas.2011.08.005

4 OECD. 'Are children active and physically healthy?',. In: *Measuring What Matters for Child Well-Being and Policies*. Paris: : OECD Publishing 2021. 1–295. doi:10.1787/e82fded1-en

5 OECD. Education in the Digital Age. In: Burns T, Gottschalk F, eds. *Education in the Digital Age*. OECD 2020. 1–218. doi:10.1787/1209166A-EN

6 Pyper E, Harrington D, Manson H. The impact of different types of parental support behaviours on child physical activity, healthy eating, and screen time: a cross-sectional study. Published Online First: 2016. doi:10.1186/s12889-016-3245-0

7 Edwardson CL, Gorely T. Parental influences on different types and intensities of physical activity in youth: A systematic review. *Psychol Sport Exerc* 2010;**11**:522–35.

For peer review only - http://bmjopen.bmj.com/site/about/guidelines.xhtml

doi:10.1016/j.psychsport.2010.05.001

8 Pawellek M, Kopf FM, Egger N, *et al.* Pathways linking parental health literacy with health behaviours directed at the child: a scoping review. *Health Promot Int* 2021;**2021**:1–29. doi:10.1093/HEAPRO/DAAB154

9 Moore SA, Faulkner G, Rhodes RE, *et al.* Impact of the COVID-19 virus outbreak on movement and play behaviours of Canadian children and youth: A national survey. *Int J Behav Nutr Phys Act* 2020;**17**. doi:10.1186/s12966-020-00987-8

10 Chowdhury R, Van Daalen KR, Franco OH. Cardiometabolic Health: Key in Reducing Adverse COVID-19 Outcomes. *Glob Heart* 2020;**15**:58. doi:10.5334/gh.879

11 Koch R, Jordan S, Starker A, *et al.* Health behaviour and COVID-19: Initial findings on the pandemic Journal of Health Monitoring 2. *J Heal Monit* 2020;**2020**:1–14. doi:10.25646/7055

12 Childs CE, Calder PC, Miles EA. Diet and Immune Function. *Nutrients* 2019;**11**. doi:10.3390/nu11081933

13 Lange KW, Nakamura Y. Lifestyle factors in the prevention of COVID-19. *Glob Heal J* 2020;**4**:146–52. doi:10.1016/J.GLOHJ.2020.11.002

14 Chastin SFM, Abaraogu U, Bourgois JG, *et al.* Effects of Regular Physical Activity on the Immune System, Vaccination and Risk of Community-Acquired Infectious Disease in the General Population: Systematic Review and Meta-Analysis. *Sports Med* 2021;**51**:1673. doi:10.1007/S40279-021-01466-1

15 Furman D, Campisi J, Verdin E, *et al.* Chronic inflammation in the etiology of disease across the life span. *Nat Med 2019 2512* 2019;**25**:1822–32. doi:10.1038/s41591-019-0675-0

16 Wintergerst ES, Maggini S, Hornig DH. Contribution of selected vitamins and trace

For peer review only

For peer review only - http://bmjopen.bmj.com/site/about/guidelines.xhtml

elements to immune function. *Ann Nutr Metab* 2007;**51**:301–23. doi:10.1159/000107673

17 Laddu DR, Lavie CJ, Phillips SA, *et al.* Physical activity for immunity protection: Inoculating populations with healthy living medicine in preparation for the next pandemic. *Prog Cardiovasc Dis* 2021;**64**:102–4. doi:10.1016/J.PCAD.2020.04.006

18 Hamer M, Kivimäki M, Gale C, *et al.* Lifestyle risk factors, inflammatory mechanisms, and COVID-19 hospitalization: A community-based cohort study of 387,109 adults in UK. *Brain Behav Immun* 2020;**87**:184–7. doi:10.1016/J.BBI.2020.05.059

19 Lee SW, Lee J, Moon SY, *et al.* Physical activity and the risk of SARS-CoV-2 infection, severe COVID-19 illness and COVID-19 related mortality in South Korea: a nationwide cohort study. *Br J Sports Med* 2021;**0**:18. doi:10.1136/BJSPORTS-2021-104203

20 Zhou B, Yuan Y, Wang S, *et al.* Risk profiles of severe illness in children with COVID-19: a meta-analysis of individual patients. *Pediatr Res 2021 902* 2021;**90**:347–52. doi:10.1038/s41390-021-01429-2

21 Shekerdemian LS, Mahmood NR, Wolfe KK, *et al.* Characteristics and Outcomes of Children With Coronavirus Disease 2019 (COVID-19) Infection Admitted to US and Canadian Pediatric Intensive Care Units. *JAMA Pediatr* 2020;**174**:868–73. doi:10.1001/JAMAPEDIATRICS.2020.1948

22 Castagnoli R, Votto M, Licari A, *et al.* Severe Acute Respiratory Syndrome Coronavirus 2 (SARS-CoV-2) Infection in Children and Adolescents: A Systematic Review. *JAMA Pediatr* 2020;**174**:882–9. doi:10.1001/JAMAPEDIATRICS.2020.1467

23 OECD. Combatting COVID-19's effect on children - OECD. 2020.https://www.oecd.org/coronavirus/policy-responses/combatting-covid-19-s-effect-on-children-2e1f3b2f/ (accessed 1 Oct 2021).

For peer review only

24 Senedd Research. Coronavirus timeline: Welsh and UK governments' response – IN BRIEF. 2020.https://seneddresearch.blog/2020/03/19/covid-19-timeline-welsh-and-uk-governments-response/ (accessed 30 Mar 2022).

25 Todd, C., Christian, D., Tyler, R., Stratton, G., Brophy S. Developing HAPPEN (Health and Attainment of Pupils involved in a Primary Education Network): working in partnership to improve child health and education. *Perspect Public Health* 2016;**136**:115–6.

26 Christian D, Todd C, Davies H, *et al.* Community led active schools programme (CLASP) exploring the implementation of health interventions in primary schools: headteachers' perspectives. *BMC Public Health* 2015;**15**:238. doi:10.1186/s12889-015-1557-0

27 Todd C, Christian D, Davies H, *et al.* Headteachers' prior beliefs on child health and their engagement in school based health interventions: a qualitative study. *BMC Res Notes* 2015;**18**:161. doi:10.1186/s13104-015-1091-2

28 Marchant E, Todd C, Cooksey R, *et al.* Curriculum-based outdoor learning for children aged 9-11: A qualitative analysis of pupils' and teachers' views. *PLoS One* 2019;**14**. doi:10.1371/journal.pone.0212242

29 Marchant E, Todd C, Stratton G, *et al.* The Daily Mile: Whole-school recommendations for implementation and sustainability. A mixed-methods study. *PLoS One* 2020;**15**. doi:10.1371/journal.pone.0228149

30 Marchant E, Griffiths L, Crick T, *et al.* COVID-19 mitigation measures in primary schools and association with infection and school staff wellbeing: An observational survey linked with routine data in Wales, UK. *PLoS One* 2022;**17**:e0264023. doi:10.1371/JOURNAL.PONE.0264023

31 Everson B, Mackintosh KA, McNarry MA, *et al.* Can Wearable Cameras be Used to

For peer review only

For peer review only - http://bmjopen.bmj.com/site/about/guidelines.xhtml

Validate School-Aged Children’s Lifestyle Behaviours? *Children* 2019;**6**:20. doi:10.3390/children6020020

32 Department of Health & Social Care. COVID-19 testing data: methodology note - GOV.UK. 2020.https://www.gov.uk/government/publications/coronavirus-covid-19-testing-data-methodology/covid-19-testing-data-methodology-note (accessed 30 Mar 2022).

33 Ford D V., Jones KH, Verplancke JP, *et al.* The SAIL Databank: Building a national architecture for e-health research and evaluation. *BMC Health Serv Res* 2009;**9**. doi:10.1186/1472-6963-9-157

34 Jones KH, Ford DV, Thompson S, *et al.* A Profile of the SAIL Databank on the UK Secure Research Platform. *Int J Popul Data Sci* 2019;**4**. doi:10.23889/ijpds.v4i2.1134

35 Lyons J, Akbari A, Torabi F, *et al.* Understanding and responding to COVID-19 in Wales: protocol for a privacy-protecting data platform for enhanced epidemiology and evaluation of interventions. *BMJ Open* 2020;**10**:43010. doi:10.1136/bmjopen-2020-043010

36 Benchimol EI, Smeeth L, Guttmann A, *et al.* The REporting of studies Conducted using Observational Routinely-collected health Data (RECORD) Statement. *PLOS Med* 2015;**12**:e1001885. doi:10.1371/JOURNAL.PMED.1001885

37 Welsh Government. Welsh Index of Multiple Deprivation. 2019.https://gov.wales/welsh-index-multiple-deprivation-full-index-update-ranks-2019

38 Dong Y, Peng CYJ. Principled missing data methods for researchers. *Springerplus* 2013;**2**:1–17. doi:10.1186/2193-1801-2-222

39 Sørensen K, Van den Broucke S, Fullam J, *et al.* Health literacy and public health: A

For peer review only

For peer review only - http://bmjopen.bmj.com/site/about/guidelines.xhtml

systematic review and integration of definitions and models. Published Online First: 2012. doi:10.1186/1471-2458-12-80

40 Morrison AK, Glick A, Yin HS. Health Literacy: Implications for Child Health. *Pediatr Rev* 2019;**40**:263–77. doi:10.1542/PIR.2018-0027

41 Bourdieu P. The Forms of Capital. In: *Handbook of Theory and Research for the Sociology of Education*. New York: : Greenwood 1986. 241–58.

42 Darling N, Steinberg L. Parenting Style as Context: An Integrative Model. *Psychol Bull* 1993;**113**:487–96. doi:10.1037/0033-2909.113.3.487

43 Li L, Moosbrugger ME. Correlations between Physical Activity Participation and the Environment in Children and Adolescents: A Systematic Review and Meta-Analysis Using Ecological Frameworks. *Int J Environ Res Public Heal 2021, Vol 18, Page 9080* 2021;**18**:9080. doi:10.3390/IJERPH18179080

44 Welsh Government. Reopening children's playgrounds and outdoor play areas: coronavirus. Guidance for those in charge of playgrounds or outdoor play areas to keep children safe during the coronavirus pandemic. 2020. https://gov.wales/reopening-childrens-playgrounds-and-outdoor-play-areas-coronavirus-html (accessed 25 Apr 2022).

45 Mehta CM, Strough JN. Sex segregation in friendships and normative contexts across the life span. *Dev Rev* 2009;**29**:201–20. doi:10.1016/J.DR.2009.06.001

46 Edwards CP, Knoche L, Kumru A. Play Patterns and Gender. *Encycl women Gend Sex similarities Differ impact Soc Gend* 2001;:809–15.http://digitalcommons.unl.edu/psychfacpub/607/ (accessed 25 Apr 2022).

47 Viner RM, Mytton OT, Bonell C, *et al.* Susceptibility to SARS-CoV-2 Infection among Children and Adolescents Compared with Adults: A Systematic Review and Meta-

For peer review only - http://bmjopen.bmj.com/site/about/guidelines.xhtml

analysis. JAMA Pediatr. 2020. doi:10.1001/jamapediatrics.2020.4573

48 Beale S, Braithwaite I, Navaratnam AM, *et al.* Deprivation and exposure to public activities during the COVID-19 pandemic in England and Wales. *J Epidemiol Community Heal* 2022;**76**:319–26. doi:10.1136/jech-2021-217076

Figure 1: Cohort flow diagram

For peer review only

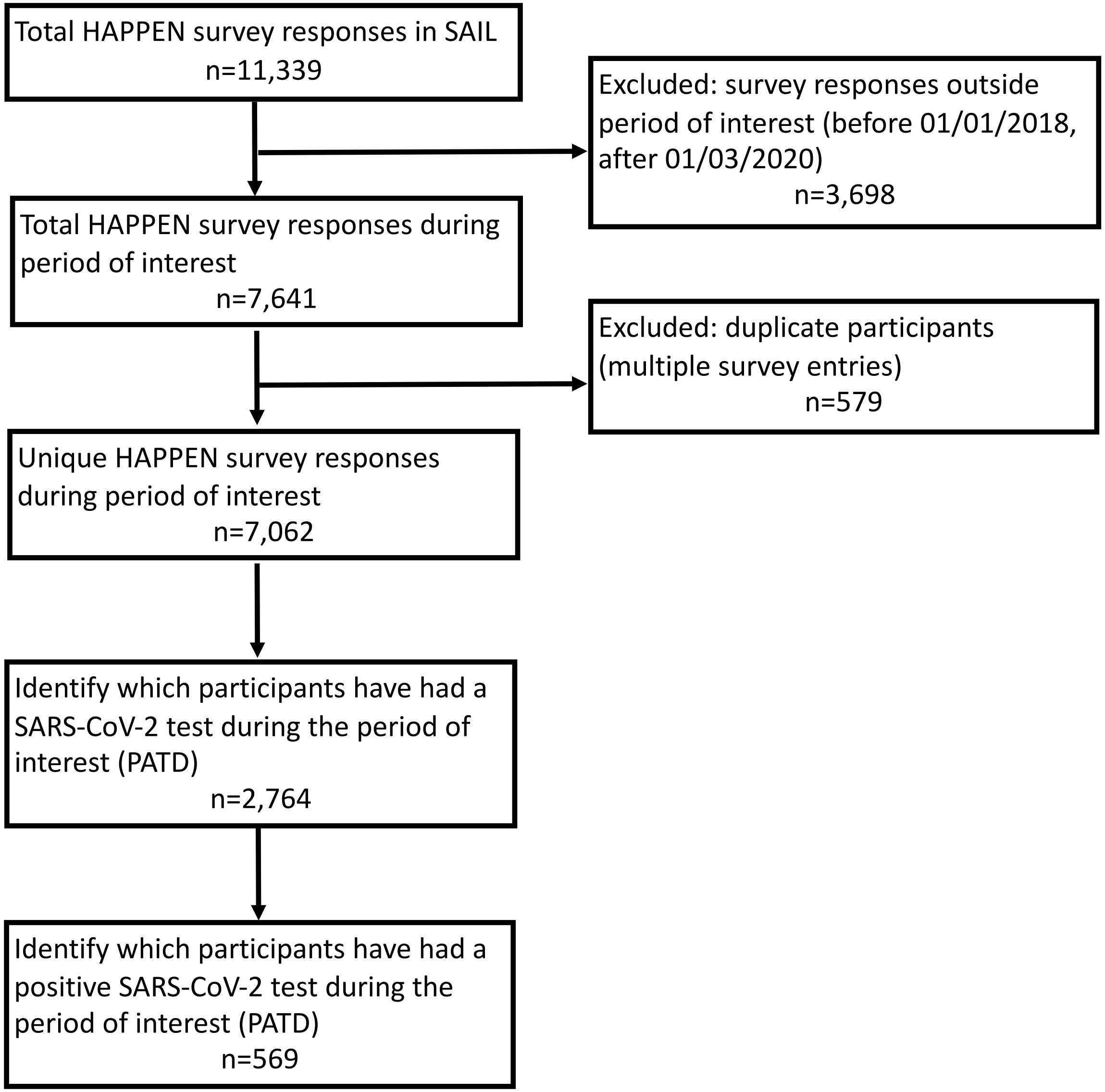
Total HAPPEN survey responses in SAIL
n=11,339
Excluded: survey responses outside period of interest (before 01/01/2018, after 01/03/2020)
n=3,698
Total HAPPEN survey responses during period of interest
n=7,641
Excluded: duplicate participants (multiple survey entries)
n=579
Unique HAPPEN survey responses during period of interest
n=7,062
Identify which participants have had a SARS-CoV-2 test during the period of interest (PATD)
n=2,764
Identify which participants have had a positive SARS-CoV-2 test during the period of interest (PATD)
n=569

Online supplemental appendix 1: see [24]

1 March 2020	Start of study period.
20 March 2020	All schools across Wales closed, with the exception of provision of vulnerable children and children of critical workers.
28 April 2020	More drive-through testing centres.
13 May 2020	Test, Trace, Protect published including testing strategy to expand from testing workers in hospitals and care comes to symptomatic community testing.
18 May 2020	Home testing rolled out, enabling symptomatic people to request home coronavirus test.
1 June 2020	Contract tracing began in Wales. Anyone who tested positive for coronavirus contacted by contact tracer and asked to provide details of everyone they had been in close contact with. Those close contacts contacted and asked to self-isolate for 14 days.
29 June 2020	Schools opened to pupils from all year groups for limited periods during the week, with only a third of pupils in school at any one time.
15 July 2020	New Wales coronavirus testing strategy released.
17–24 July 2020	All schools in Wales closed for the summer holidays.
18 August 2020	Further investment in testing. Welsh Government announced £32 million funding to improve coronavirus testing including speed of processing tests and ensuring robust testing and contract tracing system for anticipated second wave.
1 September 2020	Some schools operated a phased return with flexibility to priority groups.
14 September 2020	Schools opened to all pupils in Wales.
17 September 2020	Testing update. The Health Minister provided an update on Wales' response to current challenges with coronavirus testing, including a significant increase in demand for testing.
24 September 2020	NHS COVID-19 app launched for people aged 16 and over, including increased capacity for identifying contacts of those tested positive for coronavirus.
29 September 2020	Wales' Minister for Health and Social Care sets out the prioritisation for Covid-19 testing in Wales as the Welsh Government move into a new phase of its response. The Minster set out six priorities for testing, with those working in education or childcare settings the fifth priority group and all symptomatic individuals being sixth.
19–30 October 2020	Autumn half term holiday (three local authorities had 2 week half term)
2 November 2020	Pupils in year 9 and above not expected to be present in school due to firebreak in Wales.
16 November 2020	New financial support scheme launched for people who need to self-isolate due to a positive coronavirus test result or those asked to do so by NHS Wales Test, Trace, Protect.
18 November 2020	Merthyr Tydfil (one of 22 local authorities) to be first whole area testing pilot in Wales. Everyone offered Covid-19 testing, whether symptomatic or asymptomatic. The mass testing programme used LFTs.
27 November 2020	Mass testing extended (use of LFTs).
14 December 2020	All secondary schools in Wales moved to online remove learning for last week of term before Christmas (14-18 December 2020). Many primary schools also closed.
14 December 2020	Plans for serial testing in schools. The Welsh Government announces plans to roll out serial testing in schools and colleges from January 2021.
18 December 2020	Testing infrastructure developments to support mass testing of symptomatic people across the Welsh population. In addition to increasing testing infrastructure for people with symptoms, also developing approach and support for people without symptoms to access lateral flow tests (LFT).

For peer review only

Online supplemental appendix 1: see [24]

4 January 2021	All schools across Wales closed and moved to online remote learning, with the exception of provision for vulnerable children and children of critical workers.
28 January 2021	Publication of an updated coronavirus testing strategy including to continue to test symptomatic individuals.
1 February 2021	Users of NHS Covid-19 app could apply for self-isolation payment. People on low incomes who are asked to self-isolate via the NHS Covid-19 are eligible to apply for the £500 self-isolation support payment, alongside those who have been asked to self-isolate by the Test, Trace, Protect service and parents/carers whose child has been asked to self-isolate by their education setting.
5 February 2021	Twice weekly testing in schools. The Health Minister announces that daily contact testing in schools and colleges will be paused and instead, twice weekly testing using LFTs. Guidance for detection of positive LFT encourages follow up PCR test.
27 February 2021	Testing offer extended to upper secondary and college learners. Welsh Government Ministers announce that they are extending the offer of regular, twice weekly, LFTs at home to all those of upper secondary age. This will start with offering tests to years 11 to 13, and to all further education college learners and those on work-based apprenticeship and traineeship programmes.
10 March 2021	Testing for close contacts of positive cases. Wales' Health Minister, announces that people who are close contacts of someone who has tested positive for coronavirus and have been asked to isolate by contact tracers will now be offered a coronavirus test. Also extra £50 million to allow health boards to extend contact tracing over the summer.
15 March 2021	All remaining primary school pupils and secondary pupils in qualification years 11 and 13 able to return to learning on site. Other secondary years were able to return for check ins.
15 March 2021	Voluntary asymptomatic testing offer available to secondary age pupils in years 10 and above.
22 March 2021	Community asymptomatic testing programme extended to end of September 2021.
30 March 2021	Updated testing strategy published.
12 April 2021	All remaining pupils were able to return to learning on site.
13 April 2021	Voluntary asymptomatic testing offer extended to all secondary school age years (years 7 and above).
26 May 2021	Covid tests (LFTs) encouraged for people holidaying in Wales.
31 August 2021	End of study period.

For peer review only

Online supplemental appendix 2: RECORD statement

The RECORD statement – checklist of items, extended from the STROBE statement, that should be reported in observational studies using routinely collected health data.

	Item No.	STROBE items	Location in manuscript where items are reported	RECORD items	Location in manuscript where items are reported
**Title and abstract**					
	1	(a) Indicate the study's design with a commonly used term in the title or the abstract (b) Provide in the abstract an informative and balanced summary of what was done and what was found		RECORD 1.1: The type of data used should be specified in the title or abstract. When possible, the name of the databases used should be included.<br><br>RECORD 1.2: If applicable, the geographic region and timeframe within which the study took place should be reported in the title or abstract.<br><br>RECORD 1.3: If linkage between databases was conducted for the study, this should be clearly stated in the title or abstract.	1.1: Title and abstract<br><br>1.2: Title and abstract
**Introduction**					
Background rationale	2	Explain the scientific background and rationale for the investigation being reported			Background
Objectives	3	State specific objectives, including any prespecified hypotheses			Background
**Methods**					
Study Design	4	Present key elements of study design early in the paper			Methods - Study design
Setting	5	Describe the setting, locations, and relevant dates, including			Methods - Study design

For peer review only

Online supplemental appendix 2: RECORD statement

		periods of recruitment, exposure, follow-up, and data collection			
Participants	6	*(a) Cohort study* - Give the eligibility criteria, and the sources and methods of selection of participants. Describe methods of follow-up<br>*Case-control study* - Give the eligibility criteria, and the sources and methods of case ascertainment and control selection. Give the rationale for the choice of cases and controls<br>*Cross-sectional study* - Give the eligibility criteria, and the sources and methods of selection of participants<br><br>*(b) Cohort study* - For matched studies, give matching criteria and number of exposed and unexposed<br>*Case-control study* - For matched studies, give matching criteria and the number of controls per case		RECORD 6.1: The methods of study population selection (such as codes or algorithms used to identify subjects) should be listed in detail. If this is not possible, an explanation should be provided.<br><br>RECORD 6.2: Any validation studies of the codes or algorithms used to select the population should be referenced. If validation was conducted for this study and not published elsewhere, detailed methods and results should be provided.<br><br>RECORD 6.3: If the study involved linkage of databases, consider use of a flow diagram or other graphical display to demonstrate the data linkage process, including the number of individuals with linked data at each stage.	6.1: Figure 1: Cohort Flow Diagram<br><br>6.3: Figure 1: Cohort Flow Diagram
Variables	7	Clearly define all outcomes, exposures, predictors, potential confounders, and effect modifiers. Give diagnostic criteria, if applicable.		RECORD 7.1: A complete list of codes and algorithms used to classify exposures, outcomes, confounders, and effect modifiers should be provided. If these cannot be reported, an explanation should be provided.	7.1: Supplemental appendix 4: HAPPEN survey variable codebook
Data sources/ measurement	8	For each variable of interest, give sources of data and details of methods of assessment (measurement).			Methods - The HAPPEN survey and linked SAIL data

For peer review only

Online supplemental appendix 2: RECORD statement

		Describe comparability of assessment methods if there is more than one group			
Bias	9	Describe any efforts to address potential sources of bias			Methods - Quantitative analysis
Study size	10	Explain how the study size was arrived at			Figure 1: Cohort flow diagram
Quantitative variables	11	Explain how quantitative variables were handled in the analyses. If applicable, describe which groupings were chosen, and why			Methods - Quantitative analysis
Statistical methods	12	(a) Describe all statistical methods, including those used to control for confounding<br>(b) Describe any methods used to examine subgroups and interactions<br>(c) Explain how missing data were addressed<br>(d) *Cohort study* - If applicable, explain how loss to follow-up was addressed<br>*Case-control study* - If applicable, explain how matching of cases and controls was addressed<br>*Cross-sectional study* - If applicable, describe analytical methods taking account of sampling strategy<br>(e) Describe any sensitivity analyses			Methods - Quantitative analysis
Data access and cleaning methods		..		RECORD 12.1: Authors should describe the extent to which the	12.1: Methods - The HAPPEN

For peer review only

Online supplemental appendix 2: RECORD statement

				investigators had access to the database population used to create the study population.<br><br>RECORD 12.2: Authors should provide information on the data cleaning methods used in the study.	survey and linked SAIL data<br><br>12.2: Figure 1 – Cohort flow diagram
Linkage		..		RECORD 12.3: State whether the study included person-level, institutional-level, or other data linkage across two or more databases. The methods of linkage and methods of linkage quality evaluation should be provided.	12.3: Methods - Study design<br><br>The HAPPEN survey and linked SAIL data
**Results**					
Participants	13	(a) Report the numbers of individuals at each stage of the study (*e.g.*, numbers potentially eligible, examined for eligibility, confirmed eligible, included in the study, completing follow-up, and analysed)<br>(b) Give reasons for non-participation at each stage.<br>(c) Consider use of a flow diagram		RECORD 13.1: Describe in detail the selection of the persons included in the study (*i.e.*, study population selection) including filtering based on data quality, data availability and linkage. The selection of included persons can be described in the text and/or by means of the study flow diagram.	13.1: Methods - Quantitative analysis<br><br>Figure 1: Cohort flow diagram
Descriptive data	14	(a) Give characteristics of study participants (*e.g.*, demographic, clinical, social) and information on exposures and potential confounders<br>(b) Indicate the number of participants with missing data for each variable of interest<br>(c) *Cohort study* - summarise follow-up time (*e.g.*, average and total amount)			Results - Table 1 Descriptive statistics<br>Full descriptive statistics table: Online supplemental appendix 5

For peer review only

Online supplemental appendix 2: RECORD statement

Outcome data	15	*Cohort study* - Report numbers of outcome events or summary measures over time<br>*Case-control study* - Report numbers in each exposure category, or summary measures of exposure<br>*Cross-sectional study* - Report numbers of outcome events or summary measures			Results - Table 1 Descriptive statistics<br>Full descriptive statistics table: Online supplemental appendix 6
Main results	16	(a) Give unadjusted estimates and, if applicable, confounder-adjusted estimates and their precision (e.g., 95% confidence interval). Make clear which confounders were adjusted for and why they were included<br>(b) Report category boundaries when continuous variables were categorized<br>(c) If relevant, consider translating estimates of relative risk into absolute risk for a meaningful time period			Results – Table 3 and table 4<br><br>Online supplemental appendix 6: Unadjusted multivariable logistic regression analyses
Other analyses	17	Report other analyses done—e.g., analyses of subgroups and interactions, and sensitivity analyses			Results
**Discussion**					
Key results	18	Summarise key results with reference to study objectives			Results
Limitations	19	Discuss limitations of the study, taking into account sources of potential bias or imprecision. Discuss both direction and magnitude of any potential bias		RECORD 19.1: Discuss the implications of using data that were not created or collected to answer the specific research question(s). Include discussion of misclassification bias,	Strengths and limitations

Online supplemental appendix 2: RECORD statement

				unmeasured confounding, missing data, and changing eligibility over time, as they pertain to the study being reported.	
Interpretation	20	Give a cautious overall interpretation of results considering objectives, limitations, multiplicity of analyses, results from similar studies, and other relevant evidence			Discussion Conclusion
Generalisability	21	Discuss the generalisability (external validity) of the study results			Discussion Conclusion Strengths and limitations
**Other Information**					
Funding	22	Give the source of funding and the role of the funders for the present study and, if applicable, for the original study on which the present article is based			Funding
Accessibility of protocol, raw data, and programming code		..		RECORD 22.1: Authors should provide information on how to access any supplemental information such as the study protocol, raw data, or programming code.	Availability for data and materials

*Reference: Benchimol EI, Smeeth L, Guttmann A, Harron K, Moher D, Petersen I, Sørensen HT, von Elm E, Langan SM, the RECORD Working Committee. The REporting of studies Conducted using Observational Routinely-collected health Data (RECORD) Statement. *PLoS Medicine* 2015; in press.

*Checklist is protected under Creative Commons Attribution (CC BY) license.

THE HAPPEN SURVEY

* Required

Consent Form

Before you start please click this link to read the information sheet -> https://happen-wales.co.uk/wp-content/uploads/2019/02/Child-Consent-2019.pdf

1. I have read the child information sheet -> https://happen-wales.co.uk/wp-content/uploads/2019/02/Child-Consent-2019.pdf (click the link if you haven't read it) and understand that if I take part I can change my mind at any time, and this will not be a problem at all. *

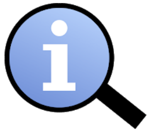

Mark only one oval.

- Yes
- No

2. I am happy for you to use my questionnaire for research. Only the researchers in the team will know my name and will not tell anyone else my answers *

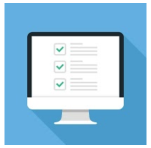

Mark only one oval.

- Yes
- No do not use my questionnaire

3. I am happy for you to look at my school and health records to see how my school is doing (as a group). This is anonymous which means I cannot be identified *

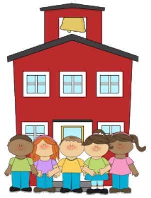

Mark only one oval.

- Yes
- No

If you do not wish to take part in the questionnaire please do not continue.

Please click next to start the questionnaire!

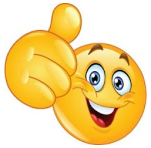

About You

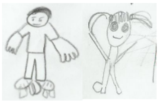

4. First Name *

5. Last Name *

6. Home Post Code *

7. What school do you go to? *

8. What year are you in? *

Mark only one oval.

- Year 4
- Year 5
- Year 6
- Year 7

9. Gender *

Mark only one oval.

- Boy
- Girl
- Prefer not to say

Date of Birth

10. Year *

Mark only one oval.

- 2007
- 2008
- 2009
- 2010
- 2011
- 2012

11. Month *

Mark only one oval.

- ( ) January
- ( ) February
- ( ) March
- ( ) April
- ( ) May
- ( ) June
- ( ) July
- ( ) August
- ( ) September
- ( ) October
- ( ) November
- ( ) December

12. Day *

Mark only one oval.

- ( ) 1
- ( ) 2
- ( ) 3
- ( ) 4
- ( ) 5
- ( ) 6
- ( ) 7
- ( ) 8
- ( ) 9
- ( ) 10
- ( ) 11
- ( ) 12
- ( ) 13
- ( ) 14
- ( ) 15
- ( ) 16
- ( ) 17
- ( ) 18
- ( ) 19
- ( ) 20
- ( ) 21
- ( ) 22
- ( ) 23
- ( ) 24
- ( ) 25
- ( ) 26
- ( ) 27
- ( ) 28
- ( ) 29
- ( ) 30
- ( ) 31

YESTERDAY

Child Health and Activity Tool

Firstly, think carefully about what you did YESTERDAY and then answer the following questions....

13. 1. What did you eat for breakfast YESTERDAY? *

Check all that apply.

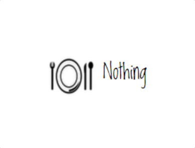

- [ ] Nothing

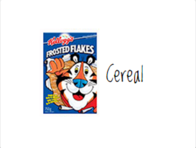

- [ ] Sugary cereal e.g. cocopops, frosties, sugar puffs, chocolate cereals

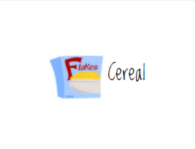

- [ ] Healthy cereal e.g. porridge, weetabix, readybrek, muesli, branflakes, cornflakes

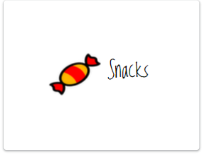

- [ ] Snacks

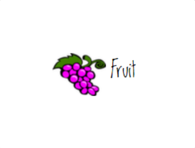

- [ ] Fruit

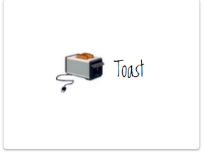

- [ ] Toast

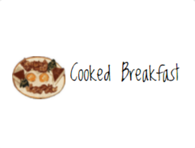

- [ ] Cooked breakfast

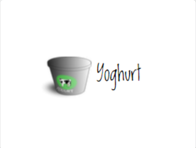

- [ ] Yoghurt

Other: [ ] ____________

14. 2. How did you get to school YESTERDAY morning? *

Mark only one oval.

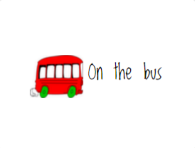

◯ On the bus

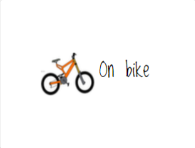

◯ On bike

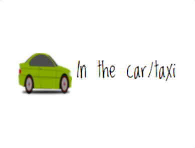

◯ In the car/taxi

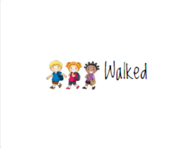

◯ Walked

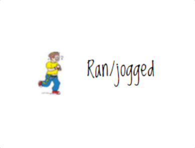

◯ Ran/jogged

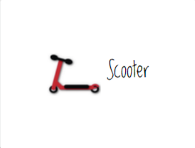

◯ Scooter

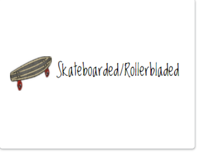

◯ Skateboarded/Rollerbladed

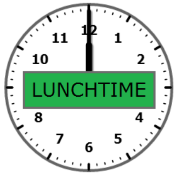

15. 3. What did you have to eat for lunch YESTERDAY? *

Mark only one oval.

◯ School dinner
◯ Packed lunch
◯ Nothing

16. 4. What did you do for MOST of your break-times YESTERDAY? (This includes lunchtime) *

Mark only one oval.

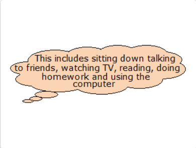

◯ Sat around inside or outside

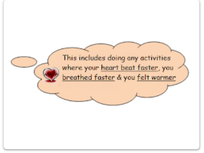

◯ Ran around

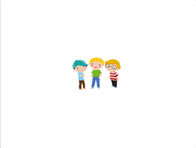

◯ Stood around

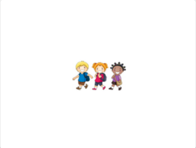

◯ Walked around

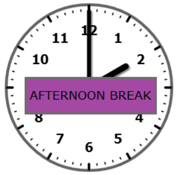

17. 5. Do you have an afternoon break at school? *

Mark only one oval.

◯ YES
◯ NO

For peer review only

18. 6. How did you get home YESTERDAY? *

Mark only one oval.

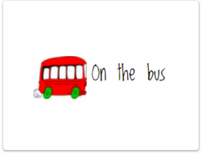

- On the bus

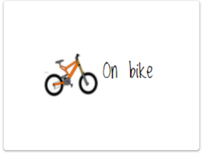

- On bike

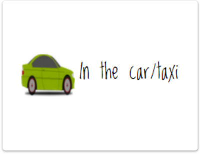

- In the car/taxi

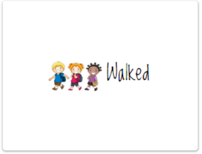

- Walked

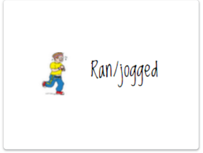

- Ran/jogged

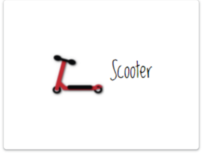

- Scooter

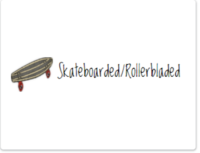

- Skateboarded/Rollerbladed

AFTER SCHOOL

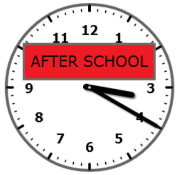

19. 7. How many portions of fruit and vegetables did you eat YESTERDAY? *

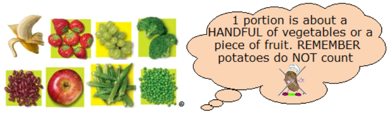

Mark only one oval.

- 0
- 1
- 2
- 3
- 4
- 5
- 6
- 7
- 8

20. 8. How many times did you brush your teeth YESTERDAY? *

Mark only one oval.

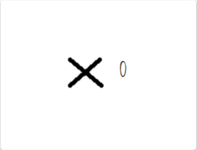

- 0

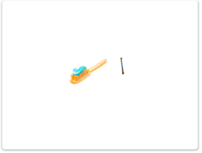

- 1

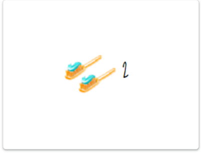

- 2

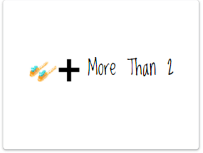

- 3

21. 9. What time did you fall asleep YESTERDAY (to the nearest half hour)? *

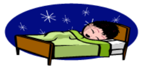

Mark only one oval.

- 7:00pm
- 7:30pm
- 8:00pm
- 8:30pm
- 9:00pm
- 9:30pm
- 10:00pm
- 10:30pm
- 11:00pm
- 11:30pm
- 12:00am
- 12:30am
- 1:00am
- 1:30am
- 2:00am
- 3:00am
- 3:30am
- 4:00am

For peer review only - http://bmjopen.bmj.com/site/about/guidelines.xhtml

22. 10. What time did you wake up TODAY (to the nearest half hour)? *

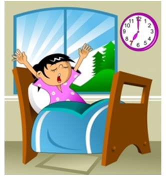

Mark only one oval.

- 5:00am
- 5:30am
- 6:00am
- 6:30am
- 7:00am
- 7:30am
- 8:00am
- 8:30am
- 9:00am

THE LAST WEEK

NOW think about what you did in the last 7 days...

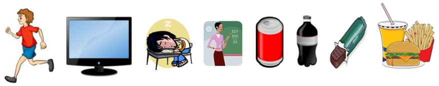

23. 11a. In the last 7 days, how many days did you do sports or exercise for at least 1 hour in total (This includes doing any activities or playing sports where your heart beat faster, you breathed faster and you felt warmer? *

Mark only one oval.

- 0 days
- 1-2 days
- 3-4 days
- 5-6 days
- 7 days

24. 11b. In the last 7 days, how many days did you watch TV/play online games/use the internet etc. for 2 or more hours a day (in total)? *

Mark only one oval.

- 0 days
- 1-2 days
- 3-4 days
- 5-6 days
- 7 days

25. 11c. In the last 7 days, how many days did you feel tired? *

Mark only one oval.

- 0 days
- 1-2 days
- 3-4 days
- 5-6 days
- 7 days

26. 11d. In the last 7 days, how many days did you feel like you could concentrate/pay attention well in class? *

Mark only one oval.

- 0 days
- 1-2 days
- 3-4 days
- 5-6 days
- 7 days

27. 11e. In the last 7 days, how many days did you drink at least one fizzy drink (e.g. coke, fanta, sprite) *

Mark only one oval.

- 0 days
- 1-2 days
- 3-4 days
- 5-6 days
- 7 days

28. 11f. In the last 7 days, how many days did you eat at least one sugary snack (e.g. chocolate bar, sweets) *

Mark only one oval.

- 0 days
- 1-2 days
- 3-4 days
- 5-6 days
- 7 days

29. 11g. In the last 7 days, how many days did you eat take away foods (e.g. McDonalds, KFC, chinese) *

Mark only one oval.

- 0 days
- 1-2 days
- 3-4 days
- 5-6 days
- 7 days

Sport and Activity

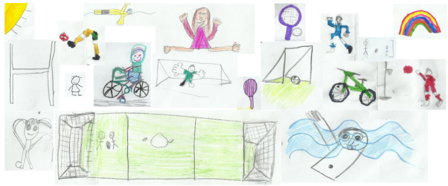

30. 12. These questions are going to ask you how you feel about physical activity (This includes any activity where your heart beats faster, you breathe faster and you feel warmer) *

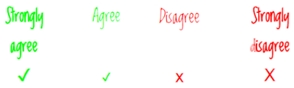

Mark only one oval per row.

	Strongly agree	Agree	Disagree	Strongly disagree
I want to take part in physical activity				
I feel confident to take part in lots of different physical activities				
I am good at lots of different physical activities				
I understand why taking part in physical activity is good for me				

For peer review only - http://bmjopen.bmj.com/site/about/guidelines.xhtml

31. 13a. How many times do you take part in a sports club OUTSIDE OF SCHOOL each week?

Mark only one oval.

0	1	2	3	4	5	6	7	8	9	10
⬭	⬭	⬭	⬭	⬭	⬭	⬭	⬭	⬭	⬭	⬭

32. 13b. If you take part in a sports club OUTSIDE of school, what is the name of the sports club? (For example Swansea Rugby Club Under 11's)

____

33. 14. Are you a member of cubs, brownies, scouts or guides? *

Mark only one oval.

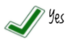

⬭ Yes

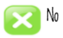

⬭ No

34. 15. Which of these sports or physical activities would you MOST like to try? (That you haven't tried before) *

Mark only one oval.

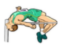

⬭ Athletics

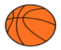

⬭ Basketball

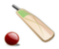

⬭ Cricket

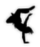

⬭ Dance

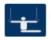

⬭ Gymnastics

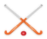

⬭ Hockey

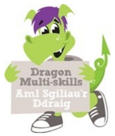

⬭ Multi Skills

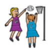

⬭ Netball

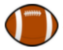

⬭ Rugby

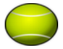

⬭ Tennis

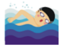

⬭ Swimming

⬭ I do not want to try anything-I don't like sport or activity

⬭ Other: __________

35. 16. Can you ride a bike WITHOUT STABILISERS? *

Mark only one oval.

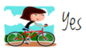

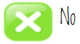

( ) Yes ( ) No

36. 17. Can you swim 25 metres WITHOUT A FLOAT OR ARMBANDS? (This is 1 length of a standard swimming pool) *

Mark only one oval.

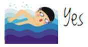

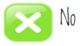

( ) Yes ( ) No

You and your feelings

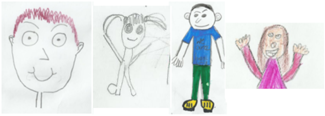

This part of the survey is going to ask you how you feel. There are no right or wrong answers. You should just pick the answer which is best for you.

37. 18. Tell us if you agree or disagree with the following: *

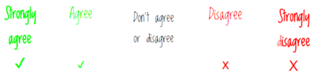

Mark only one oval per row.

	Strongly agree	Agree	Don't agree or disagree	Disagree	Strongly disagree
I am doing well at school	( )	( )	( )	( )	( )
I have lots of choice over things that are important to me	( )	( )	( )	( )	( )
There are lots of things I'm good at	( )	( )	( )	( )	( )

19. On a scale of 0 to 10 (0 being very unhappy and 10 being very happy, how do you feel about:

*Based on the Good Childhood Index by the Children's Society

38. Your Health *

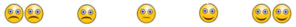

Mark only one oval.

	0	1	2	3	4	5	6	7	8	9	10	
Very unhappy	( )	( )	( )	( )	( )	( )	( )	( )	( )	( )	( )	Very happy

39. Your School *

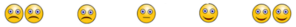

Mark only one oval.

	0	1	2	3	4	5	6	7	8	9	10	
Very unhappy	( )	( )	( )	( )	( )	( )	( )	( )	( )	( )	( )	Very happy

40. Your Family *

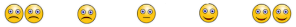

Mark only one oval.

	0	1	2	3	4	5	6	7	8	9	10	
Very unhappy	( )	( )	( )	( )	( )	( )	( )	( )	( )	( )	( )	Very happy

41. Your Friends *

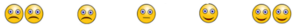

Mark only one oval.

	0	1	2	3	4	5	6	7	8	9	10	
Very unhappy	( )	( )	( )	( )	( )	( )	( )	( )	( )	( )	( )	Very happy

42. Your Appearance (how you look) *

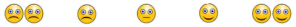

Mark only one oval.

	0	1	2	3	4	5	6	7	8	9	10	
Very unhappy	( )	( )	( )	( )	( )	( )	( )	( )	( )	( )	( )	Very happy

43. Your Life *

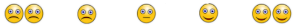

Mark only one oval.

	0	1	2	3	4	5	6	7	8	9	10	
Very unhappy	( )	( )	( )	( )	( )	( )	( )	( )	( )	( )	( )	Very happy

You and your Feelings

Based on the Me and My Feelings Questionnaire (Deighton, Tymms, Vostanis, Belsky, Fonagy, Brown, Martin, Patalay, & Wolpert, 2012)

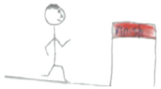

44. 20. Remember, there are no right or wrong answers, just pick which is right for you. *

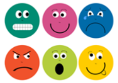

Mark only one oval per row.

	Never	Sometimes	Always
I feel lonely	◯	◯	◯
I cry a lot	◯	◯	◯
I am unhappy	◯	◯	◯
I feel nobody likes me	◯	◯	◯
I worry a lot	◯	◯	◯
I have problems sleeping	◯	◯	◯
I wake up in the night	◯	◯	◯
I am shy	◯	◯	◯
I feel scared	◯	◯	◯
I worry when I am at school	◯	◯	◯
I get very angry	◯	◯	◯
I lose my temper	◯	◯	◯
I hit out when I am angry	◯	◯	◯
I do things to hurt people	◯	◯	◯
I am calm	◯	◯	◯
I break things on purpose	◯	◯	◯

Your Local Area

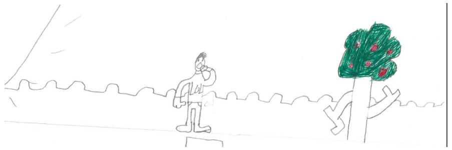

45. 21. On a scale of 0 to 10 (0 being not very safe and 10 being very safe), how safe do you feel playing in your area? *

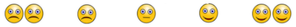

Mark only one oval.

	0	1	2	3	4	5	6	7	8	9	10	
Not very safe	◯	◯	◯	◯	◯	◯	◯	◯	◯	◯	◯	Very safe

46. 22a. From your house, can you walk to school?

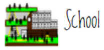

Mark only one oval.

◯ Yes
◯ No

47. 22b. From your house, can you easily walk to a park?

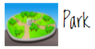

Mark only one oval.

◯ Yes
◯ No

48. 22c. From your house, can you easily walk to a leisure centre/sports centre?

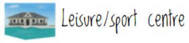

Mark only one oval.

◯ Yes
◯ No

49. 23. Are you happy with the area that you live in?

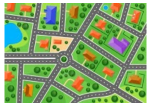

Mark only one oval.

◯ Yes
◯ No

24. If you could change something to make you and your friends healthier and happier, what would you change...

50. IN SCHOOL? *

____
____
____
____
____

51. OUT OF SCHOOL? *

____
____
____
____
____

Well done, you've completed the questionnaire.
Thank you!

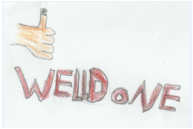

Don't forget to press submit below!

This content is neither created nor endorsed by Google.

Google Forms

For peer review only

Online supplemental appendix 4: HAPPEN survey variable codebook

Exposures	HAPPEN Survey item	Responses	Analyses coding
**Ate breakfast**	*13. What did you eat for breakfast yesterday?*	*Nothing*<br>*Cereal*<br>*Snacks*<br>*Fruit*<br>*Toast*<br>*Cooked breakfast*<br>*Yoghurt*	Binary:<br>*1 = Cereal; Snacks;*<br>*Fruit; Toast; Cooked breakfast; Yoghurt*<br>*0 = Nothing*
**Active travel to school**	*14. How did you get to school yesterday morning?*	*On the bus*<br>*In the car/taxi*<br>*Walked*<br>*On bike*<br>*Ran/jogged*<br>*Scooter*<br>*Skateboarded/rollerbladed*	Binary:<br>*1 = Walked; On bike; Ran/jogged; Scooter; Skateboarded/rollerbladed*<br>*0 = On the bus; In the car/taxi*
**Active travel from school**	*18. How did you get home yesterday?*	*On the bus*<br>*In the car/taxi*<br>*Walked*<br>*On bike*<br>*Ran/jogged*<br>*Scooter*<br>*Skateboarded/rollerbladed*	Binary:<br>*1 = Walked; On bike; Ran/jogged; Scooter; Skateboarded/rollerbladed*<br>*0 = On the bus; In the car/taxi*
**Toothbrush 2+ per day**	*20. How many times did you brush your teeth yesterday?*	*0 – 3*	Continuous:<br>*0 – 3*
**5+ fruit and veg**	*19. How many portions of fruit and vegetables did you eat yesterday?*	*0 – 8*	Continuous:<br>*0 – 8*

For peer review only

Online supplemental appendix 4: HAPPEN survey variable codebook

**Sleep 9+ hours**	*21. What time did you fall asleep last night*	*(30 min intervals)*<br>*7:00pm – 4:00am*	Continuous:<br>*Sleep hours calculated from item 21 and 22*
	22. What time did you wake up this morning?	*(30 min intervals)*<br>*5:00am – 9:00am*	
**Physically active 60+ mins every day previous 7 days**	*23. In the last 7 days, how many days did you do sports or exercise for at least 1 hour in total (This includes doing any activities or playing sports where your heart beat faster, you breathed faster and you felt warmer*	*0 days*<br>*1 – 2 days*<br>*3 – 4 days*<br>*5 – 6 days*<br>*7 days*	Ordinal:<br>*0 days*<br>*1 – 2 days*<br>*3 – 4 days*<br>*5 – 6 days*<br>*7 days*
**Sedentary/screen time 2 hours every day previous 7 days**	*24. In the last 7 days, how many days did you watch TV/play online games/use the internet etc. for 2 or more hours a day (in total)?*	*0 days*<br>*1 – 2 days*<br>*3 – 4 days*<br>*5 – 6 days*<br>*7 days*	Ordinal:<br>*0 days*<br>*1 – 2 days*<br>*3 – 4 days*<br>*5 – 6 days*<br>*7 days*
**Tired 7 days**	*25. In the last 7 days, how many days did you feel tired?*	*0 days*<br>*1 – 2 days*<br>*3 – 4 days*<br>*5 – 6 days*<br>*7 days*	Ordinal:<br>*0 days*<br>*1 – 2 days*<br>*3 – 4 days*<br>*5 – 6 days*<br>*7 days*

For peer review only

Online supplemental appendix 4: HAPPEN survey variable codebook

**Sugary snack 7 days**	*28. In the last 7 days, how many days did you eat at least one sugary snack (e.g. chocolate bar, sweets)*	*0 days*<br>*1 – 2 days*<br>*3 – 4 days*<br>*5 – 6 days*<br>*7 days*	Ordinal:<br>*0 days*<br>*1 – 2 days*<br>*3 – 4 days*<br>*5 – 6 days*<br>*7 days*
Participate in at least 3 out of school clubs	*31. How many times do you take part in a sports club OUTSIDE OF SCHOOL each week?*	*0 - 10*	Continuous:<br>*0 - 10*
Can ride a bike	*35. Can you ride a bike without stabilisers?*	*No*<br>*Yes*	Binary:<br>*1 = Yes*<br>*0 = No*
Can swim 25m	*36. Can you swim 25 metres without a float or armbands (This is 1 length of a standard swimming pool)*	*No*<br>*Yes*	Binary:<br>*1 = Yes*<br>*0 = No*
Age on 01/03/2020	*Decimal age on 1 March 2020*	Continuous	Continuous
Sex	*Sex*	*Girl*<br>*Boy*	Binary:<br>*0 = Girl*<br>*1 = Boy*
WIMD	*Welsh Index of Multiple Deprivation 2019*		Coding framework from WIMD 2019[34]

For peer review only

Online supplemental appendix 5: Full descriptive statistics table by tested for SARS-CoV-2 and tested positive for SARS-CoV-2.

		Tested for SARS-CoV-2 % (n)	Not tested for SARS-CoV-2 % (n)	Tested positive for SARS-CoV-2 % (n)	Tested negative/not tested (unknown) for SARS-CoV-2 % (n)
		**LINKED DATA**			
**Sample**		39.1% (2,764)	60.9% (4,298)	8.1% (569)	91.9% (6,493)
**Age at time of HAPPEN survey**		10.1 ± 0.8	9.9 ± 0.9	10.1 ± 0.8	9.9 ± 0.8
**Age on 01/03/2020 (start of period of interest)**		10.6 ± 0.9	10.3 ± 1.1	10.6 ± 1.0	10.4 ± 1.0
**Sex**	**Boy**	49.3% (1,363)	46.7% (2,005)	44.3% (252)	48.0% (3,116)
	**Girl**	48.9% (1,352)	51.8% (2,226)	54.5% (310)	50.3% (3,268)
	Missing	*1.8% (49)*	*1.5% (67)*	1.2% (7)	1.7% (109)
**WIMD 2019 quintiles**	**1 (most deprived)**	24.3% (672)	23.9% (1,025)	28.5% (162)	23.6% (1,535)
	**2**	19.9% (551)	19.02% (826)	19.7% (112)	19.5% (1,265)
	**3**	16.5% (455)	17.4% (748)	17.6% (100)	17.0% (1,103)
	**4**	15.6% (431)	15.8% (678)	14.1% (80)	15.9% (1,029)
	**5 (least deprived)**	18.0% (497)	16.8% (771)	16.5% (94)	17.3% (1,124)
	Missing	*5.7% (158)*	*7.0% (300)*	*3.7% (21)*	*6.7% (437)*
		**HAPPEN SURVEY**			

For peer review only

		**Previous day**			
**Ate breakfast**	**Yes**	93.0% (2,571)	92.1% (3,797)	93.4% (538)	92% (6,012)
	**No**	7% (193)	7.3% (319)	5.6% (31)	7.3% (481)
	Missing	*0%*	*0%*	*0%*	*0%*
**Active travel to school**	**Yes**	38.5% (1,065)	39.8% (1,710)	37.6% (214)	39.4% (2,561)
	**No**	61.5% (1,699)	60.2% (2,588)	62.4% (355)	60.6% (3,932)
	Missing	*0%*	*0%*	*0%*	*0%*
**Active travel from school**	**Yes**	43.0% (1,187)	43.0% (1,846)	42.4% (241)	43.0% (2,792)
	**No**	57.0% (1,577)	57.0% (2,452)	57.6% (328)	57.0% (3,701)
	Missing	*0%*	*0%*	*0%*	*0%*
**Toothbrush continuous**	**0**	3.3% (91)	3.4% (146)	1.9% (11)	3.5% (227)
	**1**	20.0% (552)	21.0% (903)	18.6% (106)	20.6% (1,358)
	**2**	67.1% (1,854)	65.2% (2,802)	69.6% (396)	65.2% (4,294)
	**3**	9.6% (265)	10.3% (446)	9.5% (54)	10.0% (659)
	Missing	*0.1% (<5)*	*<0.1% (<5)*	*0.4% (<5)*	*<0.1%% (<5)*
**Fruit/veg portions (continuous)**	**0**	14.3% (395)	15.3% (657)	12.5% (71)	15.1% (981)
	**1**	16.1% (445)	17.4% (749)	15.8% (90)	17.0% (1,104)
	**2**	17.7% (489)	17.5% (754)	19.5% (111)	17.4% (1,132)
	**3**	17.5% (484)	16.5% (711)	16.7% (95)	16.9% (1,110)
	**4**	12.7% (351)	11.9% (510)	13.5% (77)	12.1% (784)
	**5**	10.5% (291)	10.6% (455)	11.8% (67)	10.4% (679)
	**6**	4.5% (123)	4.3% (186)	2.8% (16)	4.5% (293)
	**7**	2.3% (63)	2.1% (92)	4.2% (24)	2.0% (131)
	**8**	4.5% (123)	4.3% (184)	3.2% (18)	4.5% (289)
	Missing	0%	0%	*0%*	*0%*

For peer review only

**Sleep hours**		9.4 ± 1.6	9.4 ± 1.6	9.4 ± 1.6	9.4 ± 1.6
**Number of days physically active ≥ 60 minutes**	**0**	6.5% (179)	7.9% (339)	4.0% (23)	7.6% (495)
	**1-2 days**	27.9% (772)	29.0% (1,246)	27.8% (158)	28.7% (1,860)
	**3-4 days**	27.5% (761)	26.2% (1,128)	30.9% (176)	26.4% (1,712)
	**5-6 days**	18.3% (505)	17.0% (731)	18.1% (103)	17.5% (1,133)
	**7 days**	19.8% (557)	19.9% (854)	19.2% (109)	19.9% (1,292)
	Missing	*0%*	*0%*	*0%*	*0%*
**Number of days sedentary/screen time ≥ two hours**	**0**	5.2% (144)	6.1% (262)	5.5% (31)	5.8% (375)
	**1-2 days**	24.2% (674)	23.5% (1,011)	24.8% (141)	23.8% (1,544)
	**3-4 days**	21.7% (599)	20.6% (886)	21.1% (120)	21.0% (1,365)
	**5-6 days**	14.0% (386)	13.8% (593)	13.9% (79)	13.9% (900)
	**7 days**	34.8% (961)	36.0% (1,546)	34.8% (198)	35.6% (2,309)
	Missing	*0%*	*0%*	*0%*	*0%*
**Number of days tired**	**0**	21.0% (582)	21.0% (903)	19.2% (109)	21.2% (1,376)
	**1-2 days**	32.4% (895)	32.0% (1,377)	35.7% (203)	31.9% (2,069)
	**3-4 days**	17.6% (487)	17.5% (754)	18.8% (107)	17.5% (1,134)
	**5-6 days**	10.0% (276)	9.3% (399)	10.5% (60)	9.5% (615)
	**7 days**	19.0% (524)	20.1% (865)	15.8% (90)	20.0% (1,299)
	Missing	*0%*	*0%*	*0%*	*0%*
**Number of days sugary snack**	**0**	6.5% (179)	7.7% (332)	6.3% (36)	7.3% (475)
	**1-2 days**	34.9% (964)	32.7% (1,407)	35.0% (199)	33.5% (2,172)
	**3-4 days**	25.3% (698)	26.7% (1,146)	25.1% (143)	26.2% (1,701)
	**5-6 days**	13.4% (371)	12.0% (515)	15.3% (87)	12.3% (799)
	**7 days**	20.0% (552)	20.9% (898)	18.3% (104)	20.7% (1,346)

For peer review only

	Missing	*0%*	*0%*	*0%*	*0%*
		**General**			
**Number of out of school clubs**	**0**	27.7% (766)	32.3% (1,387)	25.1% (143)	31.0% (2,010)
	**1**	17.9% (495)	16.9% (726)	16.0% (91)	17.4% (1,130)
	**2**	16.0% (443)	15.1% (650)	14.9% (85)	15.5% (1,008)
	**3**	11.1% (308)	10.4% (446)	13.3% (76)	10.4% (678)
	**4**	7.4% (204)	7.3% (313)	7.6% (43)	7.3% (474)
	**5**	6.2% (171)	5.8% (251)	5.8% (33)	6.0% (389)
	**6**	3.4% (95)	2.5% (109)	5.1% (29)	2.7% (175)
	**7**	3.3% (91)	2.5% (107)	5.1% (29)	2.6% (169)
	**8**	1.1% (29)	0.8% (33)	1.8% (10)	0.8% (52)
	**9**	0.9% (24)	0.7% (32)	1.2% (7)	0.8% (49)
	**10**	3.9% (107)	4.0% (174)	3.3% (19)	4.0% (262)
	Missing	*1.1% (31)*	*1.6% (70)*	*0.7% (<5)*	*1.5% (97)*
**Can ride a bike**	**Yes**	88.8% (2,444)	86.0% (3,696)	91.4% (520)	86.7% (5,641)
	**No**	11.2% (309)	14.0% (602)	8.6% (49)	13.3% (862)
	Missing	*0%*	*0%*	*0%*	*0%*
**Can swim 25m**	**Yes**	78.9% (2,180)	72.9% (3,134)	80.3% (457)	74.8% (4,857)
	**No**	21.1% (584)	27.1% (1,164)	19.7% (112)	25.2% (1,636)
	Missing	*0%*	*0%*	*0%*	*0%*

For peer review only

Online supplemental appendix 6:
Multivariable logistic regression model of health behaviour markers and probability of PCR-test without confounders.

PCR tested for SARS-CoV-2 (n=6,958, R^2=0.01)	OR	*p* value	95% CI
**Ate breakfast**	1.05	0.632	0.87 – 1.27
Reference: did not eat breakfast	1.00		
**Active travel to school**	0.92	0.238	0.80 – 1.06
Reference: did not active travel to school	1.00		
**Active travel from school**	1.08	0.273	0.94 – 1.24
Reference: did not active travel from school	1.00		
**Number of fruit/vegetable portions**	1.00	0.941	0.98 – 1.03
**Number of times teeth brushed**	0.97	0.474	0.90 – 1.05
**Sleep hours**	0.99	0.654	0.96 – 1.02
Reference: 0 days physically active ≥60 mins (previous seven days)	1.00		
**1-2 days physically active ≥ 60 mins**	1.12	0.276	0.91 – 1.38
**3-4 days physically active ≥ 60 mins**	1.14	0.221	0.92 – 1.42
**5-6 days physically active ≥ 60 mins**	1.17	0.177	0.93 – 1.47
**7 days physically active ≥ 60 mins**	1.09	0.475	0.87 – 1.37
Reference: 0 days sedentary ≥two hours (previous seven days)	1.00		
**1-2 days sedentary ≥ two hours**	1.16	0.209	0.92 – 1.46
**3-4 days sedentary ≥ two hours**	1.18	0.166	0.93 – 1.49
**5-6 days sedentary ≥ two hours**	1.15	0.275	0.90 – 1.47
**7 days sedentary ≥ two hours**	1.14	0.256	0.91 – 1.44
Reference: 0 days felt tired (previous seven days)	1.00		
**1-2 days felt tired**	0.98	0.791	0.86 – 1.13
**3-4 days felt tired**	0.99	0.881	0.84 – 1.16
**5-6 days felt tired**	1.04	0.667	0.86 – 1.26
**7 days felt tired**	0.97	0.730	0.83 – 1.14
Reference: 0 days consumed sugary snack (previous seven days)			
**1-2 days consumed sugary snack**	1.21*	0.062	0.99 – 1.49
**3-4 days consumed sugary snack**	1.08	0.489	0.87 – 1.33
**5-6 days consumed sugary snack**	1.29**	0.034	1.02 – 1.63
**7 days consumed sugary snack**	1.12	0.314	0.90 – 1.39
**Number of out of school clubs participation**	1.02	0.121	1.00 to 1.04
**Can ride a bike**	1.16*	0.064	0.99 – 1.35
Reference: cannot ride a bike	1.00		
**Can swim 25m**	1.30**	< 0.001	1.15 – 1.46

For peer review only

Reference: cannot swim 25m	1.00

OR: Odds Ratio; 95% CI: 95% confidence intervals; p<0.05**, p<0.1*. See online supplemental appendix 4 for variable codebook.

For peer review only

Multivariable logistic regression model of health behaviour markers and probability of PCR-test positive without confounders.

**PCR test positive for SARS-CoV-2 (n=6,958, R^2=0.01)**	**OR**	***p* value**	**95% CI**
**Ate breakfast**	1.30	0.170	0.89 – 1.91
Reference: did not eat breakfast	1.00		
**Active travel to school**	0.91	0.451	0.71 – 1.17
Reference: did not active travel to school	1.00		
**Active travel from school**	1.07	0.614	0.83 – 1.36
Reference: did not active travel from school	1.00		
**Number of fruit/vegetable portions**	0.99	0.574	0.94 – 1.03
Reference: 0 fruit/vegetable portions	1.00		
**Number of times teeth brushed**	1.07	0.385	0.92 – 1.24
Reference: did not brush teeth	1.00		
**Sleep hours**	0.97	0.266	0.92 – 1.02
Reference: 0 days physically active ≥60 mins (previous seven days)	1.00		
**1-2 days physically active ≥ 60 mins**	1.71	0.023	1.08 – 2.73
**3-4 days physically active ≥ 60 mins**	1.87	0.009	1.17 – 2.99
**5-6 days physically active ≥ 60 mins**	1.61	0.059	0.98 – 2.63
**7 days physically active ≥ 60 mins**	1.49	0.117	0.91 – 2.43
Reference: 0 days sedentary ≥two hours (previous seven days)	1.00		
**1-2 days sedentary ≥ two hours**	1.03	0.877	0.68 – 1.57
**3-4 days sedentary ≥ two hours**	1.00	0.983	0.66 – 1.54
**5-6 days sedentary ≥ two hours**	1.01	0.958	0.65 – 1.59
**7 days sedentary ≥ two hours**	1.10	0.660	0.72 – 1.66
Reference: 0 days felt tired (previous seven days)	1.00		
**1-2 days felt tired**	1.21	0.125	0.95 – 1.55
**3-4 days felt tired**	1.17	0.278	0.88 – 1.55
**5-6 days felt tired**	1.21	0.273	0.86 – 1.69
**7 days felt tired**	0.92	0.600	0.69 – 1.24
Reference: 0 days consumed sugary snack (previous seven days)	1.00		
**1-2 days consumed sugary snack**	1.14	0.499	0.78 – 1.67
**3-4 days consumed sugary snack**	1.03	0.873	0.70 – 1.53
**5-6 days consumed sugary snack**	1.38	0.131	0.91 – 2.11
**7 days consumed sugary snack**	1.04	0.867	0.69 – 1.56
**Number of out of school clubs participation**	1.05	0.007	1.01 – 1.09
**Can ride a bike**	1.40	0.032	1.03 – 1.92

Reference: cannot ride a bike	1.00		
**Can swim 25m**	1.16	0.207	0.92 – 1.45
Reference: cannot swim 25m	1.00		

For peer review only
